# Joint congress between the Pan-African Society of Cardiology and the Cardiovascular Society of Mauritius

**Published:** 2015

## Background

Facilities for Holter electrocardiography (ECG) monitoring in many Nigerian hospitals are limited. There are few published works in Nigeria on the use of 24-hour Holter ECG in cardiac arrhythmic evaluation of patients with cardiovascular diseases.

## Objective

To study the clinical indications, arrhythmic pattern and heart rate variability (HRV) among subjects referred for 24-Holter ECG at our Cardiac Care Unit.

## Methods

A total of 310 patients were studied consecutively over a 48-month period using a Schiller-type (MT-101) Holter ECG machine.

## Results

Out of the 310 patients reviewed, 134 were males (43.2%) and 176 were females (56.8%).The commonest indication for Holter ECG was palpitation, followed by syncope in 71 (23%) and 49 (15.8%) of subjects, respectively. Premature ventricular complex and premature atrial complex were the commonest type of arrhythmia in 51.5 and 15% of subjects, respectively. Ventricular arrhythmia was more prevalent in dilated cardiomyopathy patients (85.7%). The HRV of subjects with palpitation, stroke and DM with autonomic neuropathy, using SDNN average (ms), were 107.32 ± 49.61, 79.15 ± 49.15 and 66.50 ± 15.54, respectively. The HRV, using SDANN average (ms), of patients with palpitation, stroke and DM with autonomic neuropathy were 77.39 ± 62.34, 57.82 ± 37.05 and 55.50 ± 12.71, respectively.

## Conclusion

Palpitation and syncope were the commonest indications for Holter ECG among our subjects. The commonest arrhythmic patterns were premature ventricular complex and premature atrial complex, with ventricular arrhythmia being more prevalent in dilated cardiomyopathy. There was a reduction in HRV in patients with stroke and diabetic autonomic neuropathy.

