# Supplementary material for: Joint congress between the Pan-African Society of Cardiology and the Cardiovascular Society of Mauritius
Source: Cardiovasc J Afr. 2015 Sep-Oct;26(5):9–52. (PMC4683337)
Supplement: Related texts

# Abstract: Short-term outcome and determinants of outcome in heart failure in a cohort of African patients in Port Harcourt, Nigeria

**Authors:** Maclean Akpa, Okechukwu Iheji

**Affiliations:** University of Port Harcourt, Teaching Hospital, Port Harcourt, Nigeria; University of Port Harcourt, Teaching Hospital, Port Harcourt, Nigeria

## Background

Heart failure (HF) has assumed an important public health burden in Nigeria. Unfortunately there is paucity of data on the outcome pattern in patients with HF in our environment. The study aimed to determine the short-term outcome and the factors that determine these outcomes in patients treated for HF in the University of Port Harcourt Teaching Hospital (UPTH).

## Methods

It was a hospital-based prospective study. Subjects were consecutive patients with HF admitted into the medical wards of the UPTH who gave informed consent. All the subjects had full physical examinations and transthoracic echocardiography to confirm HF and determine left ventricular function. Patients were followed up for six months and reassessed for outcome/endpoint, which were rehospitalisation or death.

## Results

A total of 160 patients were studied over a one-year period, constituting 84 females and 76 males. The ages of the subjects ranged between 20 and 87 years with a mean of 52.49 ± 13.89 years. Follow-up period was six months and at the end, each patient was re-evaluated. A total of 16 subjects (10%) were lost to follow up, 66 subjects (41.3%) showed improvement clinically and continued their regular out-patient clinic attendance, 57 subjects (35.6%) were rehospitalised for HF exacerbations, while 21 subjects (13.1%) died.

The sociodemographic profile of the patients did not have any significant effect on rehospitalisation and mortality. There was a significant association between rehospitalisation and NYHA class, type of HF, body mass index, haemoglobin level, LVEF and the estimated glomerular filtration rate (eGFR). However when the effects of confounding variables were removed, the real determinants of rehospitalisation were the NYHA class, type of heart failure, haemoglobin level and the eGFR.

There was a significant association between mortality and NYHA class, haemoglobin level and LVEF. However after the effects of confounding variables were removed, the effect of LVEF disappeared, leaving only NYHA class and haemoglobin level as the real determinants of mortality.

## Conclusion

HF remains a major public health problem and the rehospitalisation rate of 35.6% and mortality rate of 13.1% in this study was high. There is therefore need to search for and control the identified determinants of these adverse outcomes.

# Abstract: Gender disparities in clinical characteristics and outcome of patients with pulmonary hypertension in Douala, Cameroon

**Authors:** Leopold Aminde, Anastase Dzudie, Sylvester Mapoh, Noah Takah, Jules Ndjebet, Xavier Kuelang, Ngu Kathleen Blackett Ngu, Karen Sliwa, Andre Pascal Kengne

**Affiliations:** Faculty of Health Sciences, University of Buea, Buea, Cameroon and Clinical Research Education, Networking and Consultancy, Douala, Cameroon; Faculty of Health Sciences, University of Buea, Buea, Cameroon; Clinical Research Education, Networking & Consultancy, Douala, Cameroon; Cardiology Unit, Department of Internal Medicine, General Hospital Douala, Douala, Cameroon; Faculty of Health Sciences, University of Cape Town, Cape Town, South Africa; Faculty of Health Sciences, University of Buea, Buea, Cameroon and Clinical Research Education, Networking and Consultancy, Douala, Cameroon; Global Health Systems Solutions, Limbe, Cameroon; Douala Cardiovascular Centre, Bonapriso, Douala, Cameroon; Universite de Montagne, Bangante, Cameroon; Department of Cardiology, University Teaching Hospital, Yaounde, Cameroon; Hatter Institute for Cardiovascular Research in Africa, Cape Town and Faculty of Health Sciences, University of Cape Town, Cape Town, South Africa; Clinical Research Education, Networking and Consultancy, Douala, Cameroon; Non-Communicable Disease Research Unit, South African Medical Research Council, Cape Town, South Africa

## Introduction

Pulmonary hypertension (PH) is an ominous prognostic feature of heart failure and has scanty epidemiological data in Africa. We explored differences in clinical and echocardiographic parameters, and outcome with regard to gender in patients with PH in Douala, Cameroon.

## Methods

This was a prospective study from March 2012 to December 2013 in which PH patients were consecutively recruited following echocardiography (RVSP ≥ 35 mmHg) from two cardiovascular centres in Douala. Patients were followed up for 12 months. Group comparisons were done using chi-square and *t*-tests while survival was estimated using Kaplan–Meier plots.

## Results

In all, 130 patients (54.6% women) with PH (78%, PH group 2) were recruited. Overall mean age was 58.7 ± 17.6 years (59.2 ± 16.1 for men and 58.3 ± 18.9 for women). While cigarette smoking and alcohol abuse were more common in men than women (both *p* < 0.001), women had greater exposure to log smoke than men (*p* = 0.00). Previous TB infection and S3 gallop rhythm were more common in women (*p* = 0.03 and *p* = 0.01, respectively). Women had a significantly higher mean SBP (*p* < 0.05) and pulse pressure (53.8 vs 44.9 mmHg, *p* = 0.01), and lower haemoglobin levels (*p* < 0.05). Echocardiographic left ventricular systolic dysfunction was found more often in men (mean LVEF: 42.6 vs 51.5%, *p* = 0.01 and mean FS: 21.4 vs 28.6, *p* = 0.01). Overall mortality rate was 39.2% and survival was better in females.

## Conclusion

Though women are more affected by PH and had high blood pressure, conventional cardiovascular risk factors were more common in men with PH. Interventions targeting control of CV risk factors should be strengthened to reduce the overall burden of PH.

# Abstract: Ischaemic gangrenous lower limb revealing a type-B aortic dissection

**Authors:** Fatimata Gatta Ba, Mohamed Issa Kharchi, Houleymata Ba, Sirakh Camara

**Affiliations:** Centre National de Cardiologie, Nouakchott, Mauritania; Centre National de Cardiologie, Nouakchott, Mauritania; Centre National de Cardiologie, Nouakchott, Mauritania; Centre National de Cardiologie, Nouakchott, Mauritania

## Introduction

Aortic dissection is a serious condition that is as a result of longitudinal tearing of the medial layer of the aorta, emanating from an intimal tear. It exposes the patient to multiple complications, including acute ischaemia of the lower limb, but this is rarely described as the main clinical manifestation of the disease.

## Case report

We describe the case of a 70-year-old man with a known history of dilated ischaemic cardiomyopathy, who was seen at our department for assessment of an acute right leg ischaemia which was gangrenous and was subsequently amputated at the mid-thigh level. On admission, he was haemodynamically stable and presented with a left basal pleural effusion and the right femoral pulse was not detected. ECG determined an incomplete left bundle branch block, an inferior necrosis and an atrial extrasystole. The chest X-ray showed cardiomegaly with a cardiothoracic ratio of 0.86 and a widened mediastinum. Transthoracic Doppler echocardiography showed a dilated descending aorta at 67 mm with an intimal flap, and a moderate circumferential pericardial effusion. The left chambers were dilated with wall motion abnormalities and a LVEF of 29%. Furthermore, the abdominal aorta was dilated at 49 mm with an intimal flap. The thoraco-abdominal CT angiography confirmed a type-B aortic dissection. The aortic isthmus was dilated with a partially thrombosed false lumen. A bilateral pleural effusion was visualised. The dissection extended to the abdominal aorta and the right iliac artery with dilatation of the right and common iliac arteries and a nearly totally thrombosed false lumen. Medical treatment was given but the patient died.

## Conclusion

The diagnosis of aortic dissection is sometimes difficult. The absence of severe chest or abdominal pain and an emboligenic heart disease, a cause of acute lower-limb ischaemia, could mask the diagnosis. In this case, it was highly suspected after transthoracic echocardiography. Endovascular treatment was not discussed due to its unavailability locally.

# Abstract: Prevalence and predictive value of the electrocardiogram in pulmonary hypertension: evidence from the Pan-African Pulmonary Hypertension Cohort (PAPUCO) study

**Authors:** Irina Balieva, Anastase Dzudie, Friedrich Thienemann, Ana O Mocumbi, Lori Blauwet, Kamilu Karaye, Mahmoud U Sani, Okechukwu S Ogah, Adriaan Voors, Andre Pascal Kengne, Karen Sliwa

**Affiliations:** Department of Medicine, Faculty of Health Sciences, University of Cape Town, South Africa; Hatter Institute for Cardiovascular Research in Africa, University of Cape Town, South Africa; University of Groningen, Groningen, the Netherlands; Department of Medicine, Faculty of Health Sciences, University of Cape Town, South Africa; Cardiology Unit, Douala General Hospital, Cameroon; Faculty of Health sciences, University of Buea, Cameroon; Department of Medicine, Faculty of Health Sciences, University of Cape Town, South Africa; Clinical Infectious Diseases Research Initiative, IDM, University of Cape Town, South Africa; Integerafrica research & development, Cape Town, South Africa; Faculty of Medicine, Eduardo Mondlane University, Maputo, Mozambique; Division of Cardiovascular Diseases, Mayo Clinic, Rochester, Minnesota, USA; Department of Medicine, Bayero University, Kano, Nigeria; Department of Medicine, Faculty of Health Sciences, University of Cape Town, South Africa; Department of Medicine, Bayero University, Kano, Nigeria; Department of Medicine, University College Hospital Ibadan, Ibadan, Nigeria; Ministry of Health, Umuahia, Nigeria; University of Groningen, Groningen, the Netherlands; Department of Medicine, Faculty of Health Sciences, University of Cape Town, South Africa; Non-Communicable Diseases Unit, South African Medical Research Council, South Africa; Department of Medicine, Faculty of Health Sciences, University of Cape Town, South Africa; Hatter Institute for Cardiovascular Research in Africa, University of Cape Town, South Africa

## Background

Pulmonary hypertension (PH) is a major public health issue worldwide. Despite improvements in understanding PH and the development of novel therapies, the condition is still diagnosed at an advanced stage, leading to a poor quality of life and survival. We assessed the diagnostic utility of a standard 12-lead ECG for PH in resource-limited settings.

## Methods

Sixty-five patients diagnosed with PH from the multicentre Pan-African Pulmonary Hypertension Cohort (PAPUCO) study were compared to 285 heart disease-free subjects from the Heart of Soweto study. Prevalence, sensitivity, specificity, and positive and negative predictive values of ECG parameters indicative of PH and right heart strain were calculated.

## Results

Of the 65 patients, 46% had PH due to left heart disease, 31% had pulmonary arterial hypertension and 22% had PH due to lung disease and/or hypoxia. Compared to the control group, all abnormalities were more frequent in the PH cohort. The most prevalent ECG abnormalities (cases vs control) were: pathological Q wave (47.7 vs 6.7%, *p* < 0.05), left ventricular hypertrophy (38.5 vs 9.8%, *p* < 0.05) and p-pulmonale (36.9 vs 20.7%, *p* < 0.05). No patient had a completely normal ECG, as opposed to 15% in the control group. The sensitivity of ECG criteria for right heart strain ranged between 6.2 and 47.7%, while specificity ranged between 79.3 and 100%. The negative predictive value ranged between 81.5 and 88.9%. The positive predictive value was lowest at 25% for RBBB and QRS rightaxis deviation (≥ 100°), and highest at 100% for QRS axis ≥ +100° combined with R/S ratio ≥ 1 or R in V1 > 7 mm

## Conclusion

ECG abnormalities were common among patients with PH but were not specific, and furthermore, those relating to right heart strain were rather less frequent. ECG patterns focusing on the R and S amplitude in the V1 and right-axis deviation had good negative predictive values for PH, but due to a high prevalence of ECG abnormalities, this should be interpreted with caution. Until further studies, other tools are needed for screening PH.

# Abstract: Left ventricular diastolic function in Nigerian children with homozygous sickle cell anaemia using echocardigraphy

**Authors:** Olukemi Tolulope Bamigboye-Taiwo, Okeniyi John Akintunde, Samuel Omokhodion

**Affiliations:** Federal Teaching Hospital, Ido-Ekiti; Department of Paediatrics, FETHI, Ido-Ekiti, Ekiti, Nigeria; Department of Paediatrics and Child Health, Obafemi Awolowo University Teaching Hospitals Complex, Ile- Ife, Osun State, Nigeria; Department of Paediatrics, University College Hospital, Oyo State, Ibadan, Nigeria

## Introduction

Sickle cell anaemia (SCA) remains the most prevalent and arduous inherited disease in Nigeria. Various adverse cardiovascular impacts have been attributed; however, a dearth of literature persists about its impact on diastolic functioning among children.

## Objective

Determination of left ventricular (LV) diastolic function in children with SCA in steady state and comparison with apparently healthy haemoglobin type AA controls.

## Methods

Observational, case–control, cross-sectional study of 50 subjects aged ≤ 15 years and 50 age- and gender-matched controls to determine and compare E-wave and A-wave velocities (m/s), E/A ratio and deceleration time (ms) using 2-D guided Doppler echocardiography. Body surface areas (BSA) and haematocrits were also assessed.

## Results

Subjects had a significantly higher E-wave velocity (1.08 vs 0.94 m/s, p = 0.0011) and A-wave velocity (0.58 vs 0.52 m/s), *p* = 0.0000135) than controls. Slightly higher E/A ratio and longer deceleration times were observed in subjects but without statistical significance (*p* = 0.481 and *p* = 0.334, respectively). E- and A-wave velocities in subjects did not correlate with age or BSA unlike in controls, which showed negative correlations. There was however a significant negative correlation between haematocrit and the A-wave velocity and E/A ratio of subjects.

## Conclusion

Even in steady state, children with SCA had relatively early as well as late diastolic filling. These indices did not correlate with age or BSA. Haematocrit showed significant correlation with late diastolic filling.

# Abstract: A decrease in invasive and non-invasive Group A streptococcal infection in South Africa, 2003–2013

**Authors:** Dylan Barth, Mark Engel, Andrew Whitelaw, Bongani Mayosi

**Affiliations:** University of Cape Town, Groote Schuur Hospital, Cape Town, South Africa; dylan.barth@uct.ac.za; University of Cape Town, Groote Schuur Hospital, Cape Town, South Africa; dylan.barth@uct.ac.za; Stellenbosch University; University of Cape Town, Groote Schuur Hospital, Cape Town, South Africa; dylan.barth@uct.ac.za

## Background

Group A *Streptococcus* (GAS) is of enormous burden in developing countries and continues to be a major cause of premature death and morbidity. The aim of this populationbased study is to describe the incidence and epidemiological characteristics of Group A streptococcal infection in South Africa.

## Methods

We performed a retrospective study of cases of invasive and non-invasive GAS isolates from January 2003 to December 2013 from the National Health Laboratory Service (NHLS) corporate data warehouse electronic database. Invasive GAS was defined as GAS isolated in culture from a sterile site, e.g. blood and cerebrospinal fluid. We abstracted demographic information, laboratory findings and microbiological data including the site of isolation. We calculated overall and agespecific rates of invasive and non-invasive GAS isolation, using annual census population estimates for South Africa (reported per 100 000 persons per year).

## Results

From 2003 to 2013, 7 453 GAS isolates were recovered from South African patients. Thirty-two per cent were from patients under 18 years of age; 63% from those 18 to 64 years old, and 6% from those 65 years or older. The mean age was 29 years (range 0–102 years). Over the 11-year period, the annual mean incidence rate (IR) for GAS isolates was 1.38 cases per 100 000 person-years (py). Seven hundred and five cases of these isolates (9.46%) met the case definition of invasive GAS, with a mean annual IR of 0.13 cases per 100 000 py. Over the study period, both invasive and non-invasive GAS isolation showed a bimodal-shaped curve.

## Conclusion

Laboratory data showed a fall in the incidence rate of GAS isolation from South African patients over the last 11 years. Within the invasive GAS isolates, IRs showed significant fluctuations over the study period.

# Abstract: Statistics on the use of cardiac electronic devices and electrophysiological procedures from 2011 to 2014 in 27 African countries: first report from the Pan-African Society of Cardiology (PASCAR) arrhythmia study group

**Authors:** Aimé Bonny, Mphammed A Talle, Kamilu M Karaye, Anastase Dzudie, Adama Kane, Mohamed Salah, Bundhoo Kaviraj, Karooa Aswinee Kumar, Ashley Chin, Dereje Ketema Belayneh

**Affiliations:** Douala Cardiovascular Research, University of Douala, Douala, Cameroon; University Hospital of Maiduguri, Nigeria; University Hospital of Kano, Nigeria; General Hospital of Douala, Cameroon; CHU Le Dantec, Dakar, Senegal; University Hospital, Khartoum, Sudan; Hospital of Rose Hill, Mauritius; Hospital of Quatre-Bornes, Mauritius; University Hospital of Cape Town, South Africa; University Hospital of Addis Ababa, Ethiopia

## Background

Lack of data on cardiac electronic devices and electrophysiological (EP) procedures in Africa is impeding the formulation of appropriate health policies on managing cardiac arrhythmias. We conducted a survey on pacing and EP activities throughout Africa.

## Methods

A questionnaire regarding activities from 2011 to 2014 was sent to EP physicians. Additional information was obtained through manufacturers or local distributors.

## Results

Twenty-seven countries were surveyed, out of which six (22%) did not report their data and five (19%) did not have any cardiac EP services. Twenty-four centres were included, of which 20 (83%) were from the public sector. No country had a centralised national registry. Among the 16 countries (76%) with facilities for implanting cardiac devices, cardiac resynchronisation therapy (CRT) was performed in nine (56%), implantable cardioverter-defibrillator (ICD) in 11 (68.7%), and EP procedures in six (37.5%) countries. Only four (25%) countries offered the full complement of EP services (pacemaker, CRT, ICD and simple/complex ablations), with none from West, Central and East Africa. Per million inhabitants, median number of centres was three (1–60) and implanting physicians was nine (2–173). The implant rates per million habitants were 36.7 (0.2–218). Re-used devices were implanted in six (37.5%) countries, accounting for up to 11% of all procedures, with a median rate of 4%. The patient charges for dual-chamber DDD) pacemaker implantation ranged from $0.00 (in countries with reimbursement policies) to $5 556 (in private clinics), with a median cost of $2 570. Wide variations in cost were observed across the countries, with a high inter-centre variability. An inverse correlation between implant rates per million inhabitants and procedure fees standardised to gross domestic product per inhabitant (correlation coefficient *r²* = –0.17) was found.

## Conclusion

Although increasing in most countries, pacemaker implantations are still sub-optimal in sub-Saharan Africa, and EP procedures are in their embryonic stages. The high cost of procedures in this setting of pay-out-of-pocket policies, underuse of recycled devices, lack of national registries, and the deficit of trained specialists limit expanding the management of arrhythmia diseases in Africa. South–south and north–south cooperation is needed.

# Abstract: Incidence of inappropriate ICD shocks and other complications in asymptomatic versus symptomatic Brugada syndrome

**Authors:** Aimé Bonny, Marcus Ngantcha, Abdeslam Bouzeman

**Affiliations:** Douala Cardiovascular Research Centre, University of Douala, Cameroon; and Pitié-Salpêtrière Hospital, Paris, France;; Statprest, Paris, France; CMC Parly 2, Le Chesnay, France

## Background

Brugada syndrome (BrS) requires implantation of a cardioverter–defibrillator (ICD) to prevent sudden cardiac death. However, the ICD indications in asymptomatic patients remain conflicting.

## Methods and Results

We compared the rate of ICD complications in asymptomatic versus symptomatic BrS patients. ICD interrogations were done every three to six months. Given the low prevalence of BrS in the general population, 10% of the risk α for the bilateral statistical test significance was chosen. We studied 51 patients, 86.5% male, mean age 47 ± 11 years at diagnosis. At diagnosis, 18 patients (35%) were asymptomatic, 25 patients (49%) experienced syncope, and eight (16%) had been resuscitated from ventricular fibrillation. During a mean follow up of 78 ± 46 months, none of asymptomatic patients experienced appropriate therapy, whereas 21.6% of symptomatic patients had one or more shocks. Overall complication rate was 27.4%. Inappropriate shocks (IS) occurred in seven patients (13.7%; mean 6.57 ± 6.94 shocks per patient), 16.14 ± 10.38 months after ICD implantation, and lead fracture was the primary cause (*n* = 4, 57.1%). The incidence of IS was higher in asymptomatic patients (*p* = 0.09). Device-related complications were similar in both groups (*p* = 1). A total of 14 patients (27.4%) had one or more complications. The mean interval from implantation to a complication was 13.91 ± 12.98 months. The most frequent complication was lead failure in nine patients (17.6%). The risk of IS and device complications at three years was 13.7 and 21.6%, respectively, and eventually remained constant over the time.

## Conclusion

This study demonstrated that ICD implantation has a high risk of complications, mainly during the early period after device implantation. A higher rate of IS as well as a very low risk of arrhythmic events in asymptomatic BrS patients advocates us to carefully evaluate this young and otherwise ‘healthy’ population in the decision-making.

# Abstract: Incidence of sudden cardiac death in sub-Saharan Africa: the Douala SCD registry

**Authors:** Aimé Bonny, Marcus Ngantcha, WA Jonas, Rene Fonga, Cecile Saka, Amougou Sylvie Ndongo

**Affiliations:** Douala Cardiovascular Research Centre, University of Douala, Cameroon; Statprest, Paris, France; Hopital de district Bonassama; Hopital de district de New-Bell; Hopital Laquintinie de Douala, Cameroun; Centre Hospitalier, Universitaire de Yaoundé, Cameroun

## Background

A population-based incidence estimate of sudden cardiac death (SCD) in sub-Saharan Africa (SSA) is unknown. We sought to determine the epidemiology of SCD in Douala, Cameroon.

## Methods and Results

During 12 months, four districts were randomly chosen in which to monitor all deaths. The COSA (‘comité de santé’) of each area registered every death, and a postgraduate fellow recorded detailed demographic and medical data for victims of natural death. A senior physician studied every case of suspected sudden death, either cardiac or extra-cardiac. For optimal thoroughness, surrounding mortuaries were checked simultaneously to match identities of victims. Established SCD was defined as a rapid, witnessed collapse leading to death within one hour of onset of symptoms, and probable SCD was defined as unexpected death within 24 hours without obvious extracardiac cause. Our registry numbered 240 000 people with 2 304 deaths. The overall mortality rate was 9.6/1 000 inhabitants/year. Four (0.3%) were sudden extra-cardiac deaths, and 58 (2.5%) were SCDs, of which 41.4% were established and 48.3% were in under 40-year-olds. The incidence rate of SCD was 11.7/100 000 personyears. Coronary artery disease or dilated cardiomyopathy was diagnosed in 13.8% cases. Out-of-hospital cardiac arrest (OHCA) occurred in 58.6% of the victims, of which 35.3% occurred at home, and 58.8% in a taxicab to hospital. Witnessed cardiac arrest was reported in 86.2% of cases, but only 7.4% of victims experienced cardiopulmonary resuscitation attempts

## Conclusion

The SCD incidence in this city of a SSA country was similar to some reports from Western populations. However, the absence of CPR attempts raises the question of developing basic life support programmes in SSA to tackle this potentially reversible lethal cardiovascular condition.

# Abstract: Blackouts and sudden death in the apparently well and young. The case of long QT syndrome: missed opportunities for diagnosis and treatment

**Authors:** Paul Brink, Althea Goosen, Marshall Heradien

**Affiliations:** Division of Medicine, University of Stellenbosch and Tygerberg Hospital, Cape Town, South Africa; Division of Medicine, University of Stellenbosch and Tygerberg Hospital, Cape Town, South Africa; Cardiologist, Cape Town, South Africa

## Background

The long QT syndrome (LQTS) is a pro-arrhythmic cardiac disorder associated with blackouts (transient loss of consciousness; TLOC). It is necessary to recognise that these events are syncope and not epilepsy or another type of attack. Sometimes the syncope-causing ventricular tachycardia may degenerate into ventricular fibrillation and death. LQTS is autosomal dominantly inherited and causal mutations (1 000+) have been identified in 13 genes.

## Methods

Through cascade screening of relatives of 26 LQTS index cases, we identified 203 living persons with the same potassium channel mutation, namely KCNQ1 A341V. Clinical histories have been collected on all of them. We also collected information on deaths of close relatives. Many symptomatic individuals with a history of blackouts were not diagnosed with LQTS. We set out to quantitate these missed diagnoses.

## Results

Of the mutation carriers, 160 (79%) experienced blackouts. Only 26% were diagnosed as LQTS and appropriately treated. Epilepsy was the diagnosis in 40%. Another 34% had layman’s explanations, or a medical diagnosis such as ‘vasovagal’ or ‘sick sinus syndrome’. A number of ‘near-drowning’ events were documented. Historic sudden deaths before age 20 years were 23. Half of these were drownings, all in able swimmers. Other examples are a girl, aged 13 years, dying on a skating rink while under treatment for epilepsy, and a boy, aged five, who ‘choked on water’.

## Conclusion

Our experience shows gross disparities in diagnosis and the consequent management in a treatable risk for sudden death. Missed opportunities for diagnoses ranged from prior events to medical encounters, and at and after the first presentation. The most common misdiagnosis was epilepsy in the living, and drowning in the dead. Missed diagnosis and misdiagnosis may be prevented by changing how both lay persons and medical professionals perceive TLOC and also near drowning and drowning events. As said by a teacher of mine: ‘You only recognise what you know’.

# Abstract: Identification of two *TNNI3* gene mutations, one novel and one arising *de novo*, in South African patients with restrictive cardiomyopathy and focal ventricular hypertrophy

**Authors:** Paul Brink, Jomien Mouton, Adriano Pellizzon, Althea Goosen, Craig Kinnear, Phillip Herbst, Hanlie Moolman-Smook

**Affiliations:** Division of General Internal Medicine, Department of Medicine, University of Stellenbosch and Tygerberg Hospital, Stellenbosch, South Africa; US/MRC Centre for TB Research, University of Stellenbosch, South Africa; Division of General Internal Medicine, Department of Medicine, University of Stellenbosch and Tygerberg Hospital, Stellenbosch, South Africa; Division of General Internal Medicine, Department of Medicine, University of Stellenbosch and Tygerberg Hospital, Stellenbosch, South Africa; US/MRC Centre for TB Research, University of Stellenbosch, South Africa; Division of General Internal Medicine, Department of Medicine, University of Stellenbosch and Tygerberg Hospital, Stellenbosch, South Africa; US/MRC Centre for TB Research, University of Stellenbosch, South Africa

## Background

The minimum criterion for the diagnosis of hypertrophic cardiomyopathy (HCM) is thickening of the left ventricular wall, typically in an asymmetrical or focal fashion, and it requires no functional deficit. Using this criterion, we identified a family with four affected individuals and a single unrelated individual essentially with restrictive cardiomyopathy (RCM). Mutations in genes coding for the thin filaments of cardiac muscle have been described in RCM and HCM with ‘restrictive features’. One such gene encodes for cardiac troponin I (*TNNI3*), a sub-unit of the troponin complex involved in the regulation of striated muscle contraction. We hypothesised that mutations in *TNNI3* could underlie this particular phenotype, and we therefore screened *TNNI3* for mutations in 115 HCM probands.

## Methods

Clinical investigation involved examination, echocardiography, chest X-ray and an electrocardiogram of both the index cases and close relatives. The study cohort consisted of 113 South African HCM probands, with and without known founder HCM mutations, and 100 ethnically matched control individuals. Mutation screening of *TNNI3* for disease-causing mutations were performed using high-resolution melt (HRM) analysis.

## Results

HRM analyses identified three previously described HCM-causing mutations (p.Pro82Ser, p.Arg162Gln, p.Arg170Gln) and a novel exonic variant (p.Leu144His). A previous study involving the same amino acid identified a p.Leu144Gln mutation in a patient presenting with RCM, with clinical features of HCM. We observed the novel p.Leu144His mutation in three siblings with clinical RCM and varying degrees of ventricular hypertrophy. The isolated index case with the *de novo* p.Arg170Gln mutation presented with a similar phenotype. Both mutations were absent in a healthy control group.

## Conclusion

We have identified a novel disease-causing p.Leu144His mutation and a *de novo* p.Arg170Gln mutation associated with RCM and focal ventricular hypertrophy, often below the typical diagnostic threshold for HCM. Our study provides information regarding *TNNI3* mutations underlying RCM in contrast to other causes of a similar presentation, such as constrictive pericarditis or infiltration of cardiac muscle, all with marked right-sided cardiac manifestations. This study therefore highlights the need for extensive mutation screening of genes encoding for sarcomeric proteins, such as *TNNI3* to identify the underlying cause of this particular phenotype.

# Abstract: Pulmonary hypertension in a rural area: prevalence, correlates and clinical features from the Shisong Cardiac Centre, Cameroon

**Authors:** Bonaventure Suiru Dzekem, Anastase Dzudie, Tchoumi Cabral Tantchou, Leopold Aminde, Ana O Mocumbi, Martin Abanda, Andre Pascal Kengne, Friedrich Thienemann, Karen Sliwa

**Affiliations:** Faculty of Health Sciences, University of Buea, Cameroon; Clinical Research Education, Networking and Consultancy, Douala, Cameroon; Faculty of Health Sciences, University of Buea, Cameroon; Clinical Research Education, Networking and Consultancy, Douala, Cameroon; Cardiology Unit, Douala General Hospital, Douala, Cameroon; Department of Medicine, Faculty of Health Sciences, University of Cape Town, Cape Town, South Africa; Shisong Cardiac Centre, Kumbo, Cameroon; Clinical Research Education, Networking and Consultancy, Douala, Cameroon; Faculty of Medicine, Eduardo Mondlane University, Maputo, Mozambique; Faculty of Health Sciences, University of Buea, Cameroon; Clinical Research Education, Networking and Consultancy, Douala, Cameroon; Department of Medicine, Faculty of Health Sciences, University of Cape Town, Cape Town, South Africa; Non-communicable Diseases Unit, South African Medical Research Council, South Africa; Department of Medicine, Faculty of Health Sciences, University of Cape Town, Cape Town, South Africa; Clinical Infectious Diseases Research Initiative, Institute of Infectious Diseases and Molecular Medicine, Faculty of Health Science, University of Cape Town, Cape Town, South Africa; Department of Medicine, Faculty of Health Sciences, University of Cape Town, Cape Town, South Africa; Hatter Institute for Cardiovascular Research in Africa, Faculty of Health Sciences, University of Cape Town, Cape Town, South Africa

## Introduction

Pulmonary hypertension (PH) is a devastating, progressive disease with increasingly debilitating symptoms and eventually death due to narrowing of the pulmonary vasculature and consecutive right heart failure. The epidemiology of PH in low- to middle-income countries is unknown, but recent data suggest a high prevalence on the African continent. We assessed the prevalence and characteristics of PH in patients attending the rural cardiac centre of Shisong, Cameroon.

## Methods

The current study forms part of the ongoing Pan-African Pulmonary Hypertension Cohort (PAPUCO) study. From September 2013 to December 2014, all consecutive patients with newly diagnosed PH were prospectively recruited. PH was diagnosed in patients with clinical suspicion and echographically measured right ventricular systolic pressure (RVSP) > 35 mmHg. PH was classified as mild (RVSP: 36–50 mmHg), moderate (RVSP: 51–60 mmHg) and severe (RVSP: > 60 mmHg).

## Results

Out of 2 194 patients who had echocardiograms done, 343 had PH (15.6%). In all, 150 (mean age 62.7 ± 18.7 years, 54.7% women) were included in these analyses. Overall mean RVSP was 68.6 mmHg, and 7.3, 29.3 and 63.3% presented with mild, moderate and severe PH, respectively. PH due to left heart disease (PHLHD) was the commonest type of PH (64.7%), with rheumatic valvular heart disease accounting for 36.1%. Body mass index, respiratory rate, left ventricular end-systolic and -diastolic diameters positively correlated with RVSP, while oxygen saturation negatively correlated with RVSP. Co-morbidities at presentation included indoor smoke from cooking (80.7%), hypertension (52.0%), family history of cardiovascular disease (50.0%), diabetes (31.3%), alcohol abuse (21.3%) and HIV (8.7%). Clinical features at presentation were dyspnoea (78.7%), fatigue (76.7%), palpitation (57.3%), cough (56.7%), jugular venous distension (68%) and peripheral oedema (66.7%). In all, 70% of patients presented in the World Health Organisation functional class III/IV.

## Conclusion

PH was common among patients in this rural cardiac centre, with PHLHD being the most frequent type. High prevalence of indoor smoking, rheumatic heart diseases and late presentation to hospital may reflect poor a socio-economic context. These findings should increase awareness of PH among physicians in this setting, thus leading to early diagnosis and management.

# Abstract: Pulmonary hypertension due to left heart disease: data from the Pan-African Pulmonary Hypertension Cohort (PAPUCO) study

**Authors:** Anastase Dzudie, Friedrich Thienemann, Ana O Mocumbi, Lori Blauwet, Kamilu M Karaye, Mahmoud U Sani, Okechukwu S Ogah, Irina Mbanze, Amam Mbakwem, Kemi Tibazarwa, Leopold Aminde, Jules Ndjebet, Tchoumi Cabral Tantchou, Albertino Damasceno, Andre Pascal Kengne, Karen Sliwa

**Affiliations:** Department of Medicine, Faculty of Health Sciences, University of Cape Town, Cape Town, South Africa; Cardiology Unit, Douala General Hospital, Douala, Cameroon; Faculty of Health Sciences, University of Buea, Cameroon; Department of Medicine, Faculty of Health Sciences, University of Cape Town, Cape Town, South Africa; Clinical Infectious Diseases Research Initiative, Institute of Infectious Diseases and Molecular Medicine, Faculty of Health Science, University of Cape Town, Cape Town, South Africa; IntegerAfrica Research & Development, Cape Town, South Africa; Faculty of Medicine, Eduardo Mondlane University, Maputo, Mozambique; Department of Medicine, Division of Cardiovascular Diseases, Mayo Clinic, Rochester, Minnesota, USA; Department of Medicine, Bayero University and Aminu Kano Teaching Hospital, Kano, Nigeria; Department of Medicine, Bayero University and Aminu Kano Teaching Hospital, Kano, Nigeria; Department of Medicine, University College Hospital Ibadan, Ibadan, Nigeria; Ministry of Health, Umuahia, Nigeria; Faculty of Medicine, Eduardo Mondlane University, Maputo, Mozambique; Department of Medicine, College of Medicine, University of Lagos, Nigeria; Hatter Institute for Cardiovascular Research in Africa, Faculty of Health Sciences, University of Cape Town, Cape Town, South Africa; Faculty of Health Sciences, University of Buea, Cameroon; Clinical Research Education, Networking and Consultancy, Douala, Cameroon; Douala Cardiovascular Centre, Douala, Cameroon; Shisong Cardiac Centre, Kumbo, Cameroon; Faculty of Medicine, Eduardo Mondlane University, Maputo, Mozambique; Department of Medicine, Faculty of Health Sciences, University of Cape Town, Cape Town, South Africa; Non-communicable Diseases Unit, South African Medical Research Council, South Africa; Hatter Institute for Cardiovascular Research in Africa, Faculty of Health Sciences, University of Cape Town, Cape Town, South Africa

## Background

Little is known about pulmonary hypertension (PH) due to left heart disease (PHLHD) in sub-Saharan Africa (SSA). We investigated the clinical profile and shortterm outcomes of patients with PHLHD from the multicentre Pan-African Pulmonary hypertension Cohort (PAPUCO) study.

## Methods

Suspected cases of PH underwent echocardiography with measurement of right ventricular systolic pressure (RVSP) and tricuspid annular plane excursion (TAPSE). PH was classified as mild (RVSP: 35–50 mmHg), moderate (RVSP: 51–60 mmHg) and severe (RVSP: > 60 mmHg). Cox models were used to relate baseline characteristics with admission and mortality during follow up.

## Results

Of 209 patients diagnosed with any PH, 144 (mean age 53.3 ± 18.5 years, 40.4% men) had PHLHD. Mean RVSP was 60.4 ± 16.7 mmHg overall, and 41.6 ± 3.4, 51.9 ± 4.4, 78.1 ± 12.5 mmHg, respectively for mild (*n* = 47), moderate (*n* = 32), and severe PH (*n* = 62). Of all patients, 72 (50.0%) had hypertension, 16 (11.1%) had diabetes, and 16 (11.1%) were HIV infected. Patients presented at advanced stage [97 (67.4%) in WHO functional class III–IV]. Left atrial diameter (β = 0.55; *p* < 0.001) and TAPSE (β = –0.99; *p* < 0.001) were independent predictors of RVSP. A total of 35 (24.3%) deaths and 43 (29.9%) hospital admissions were recorded during a median follow up of 202 days. There was a positive association between RVSP and admissions (*p* = 0.03) but none with mortality.

## Conclusion

PHLHD was the commonest cause of PH in this multi-country cohort. Left atrium size and TAPSE were predictors of RVSP in those with PHLHD, and RVSP predicted shortterm hospitalisation but not mortality. Extended follow up of a larger sample will help refine these observations.

# Abstract: Right heart remodelling in patients with pulmonary hypertension due to left heart disease at the Douala General Hospital, Cameroon: an echocardiographic study

**Authors:** Anastase Dzudie, Martin Abanda, Leopold Aminde, Félicité Kamdem, Bonaventure Dzekem

**Affiliations:** Faculty of Health Sciences, University of Buea, Cameroon; Clinical Research Education, Networking and Consultancy, Douala, Cameroon; Cardiology Unit, Douala General Hospital, Douala, Cameroon; Department of Medicine, Faculty of Health Sciences, University of Cape Town, Cape Town, South Africa; Faculty of Health Sciences, University of Buea, Cameroon; Clinical Research Education, Networking and Consultancy, Douala, Cameroon; Clinical Research Education, Networking and Consultancy, Douala, Cameroon; Cardiology Unit, Douala General Hospital, Douala, Cameroon; Clinical Research Education, Networking and Consultancy, Douala, Cameroon

## Background

Right ventricular (RV) dysfunction has been shown to occur early and has major prognostic implications for patients with group 1 pulmonary hypertension (PH), but in patients with PH due to left heart disease (LHD), data are scarce. We aimed to compare echocardiographic RV changes in patients with LHD with PH (PHLHD) and LHD without PH (LHDnPH) in Douala, Cameroon.

## Methods

This was a cross-sectional study. All participants had a detailed echocardiographic study with structural and functional RV assessment. PH was defined as an estimated right ventricular systolic pressure > 35 mmHg. Thirty-four patients with PHLHD were compared to 65 patients with LHDnPH. Comparison was done using the chi-squared test and one-way ANOVA. Data were analysed using SPSS v. 22.

## Results

Overall, mean age was 59.1 years and 56.6% were female with no age or gender difference between the LHDnPH and PHLHD groups. Patients with PHLHD had a longer duration of hypertension (9.6 vs 4.8 years, *p* = 0.001) and presented with more dyspnoea, cough, fatigue, pedal oedema and lower systolic and diastolic blood pressure. RV free wall thickness (0.8 ± 0.2 vs 0.6 ± 0.2cm, *p* = 0.014), RV basal diameter (4.1 ± 0.7 vs 3.6 ± 0.6 cm, *p* < 0.001), and RA area (21.6 ± 8.1 vs 13.9 ± 3.4 cm^2^, *p* < 0.001) were higher in those with PHLHD compared to those with LHDnPH, while tricuspid annular plane systolic excursion (TAPSE) (1.9 ± 0.5 vs 2.3 ± 0.5 cm, *p* < 0.001) was lower in those with PHLHD. Tricuspid annular tissue Doppler imaging systolic velocity was similar between the two groups.

## Conclusion

Our findings suggest that right heart changes occur in parallel with LHD and probably worsen with superimposition of PH. A multicentre study of a larger sample is warranted to confirm these findings.

# Abstract: Rationale and design of the African group A streptococcal infection registry: the AFROStrep study

**Authors:** Mark Engel, Dylan D Barth, Andrew Whitelaw, James Dale, Bongani Mayosi

**Affiliations:** Department of Medicine, Faculty of Health Sciences, Groote Schuur Hospital, University of Cape Town, South Africa; Department of Medicine, Faculty of Health Sciences, Groote Schuur Hospital, University of Cape Town, South Africa; Stellenbosch University, South Africa; University of Tennessee Health Science Center,USA; Department of Medicine, Faculty of Health Sciences, Groote Schuur Hospital, University of Cape Town, South Africa

## Introduction

Group A β-haemolytic *Streptococcus* (GAS), a gram-positive bacterium also known as *Streptococcus pyogenes*, causes skin, mucosal, systemic and autoimmune diseases. Repeated pharyngeal and skin infections with GAS may lead to serious autoimmune diseases such as acute post-streptococcal glomerulonephritis, acute rheumatic fever (ARF) and rheumatic heart disease (RHD). Invasive GAS disease (iGAS) is associated with significant morbidity and mortality in children and young adults worldwide. Increases in the number of cases of both invasive and non-invasive GAS diseases have been observed globally since the 1980s. The reasons for these observations are not well understood and have subsequently, caused many countries to commence active surveillance systems for iGAS, to closely document the epidemiology of the disease.

A patient disease registry is a powerful surveillance tool in epidemiology. Guided by research questions, registries are developed to serve multiple purposes and provide a platform to study the natural history of disease, clinical features, cost effectiveness of treatment strategies and care, to assess safety and harm, and to provide measures of improved quality of care. Registries for streptococcal surveillance have been established in some developed countries, for example Canada, England and USA, where iGAS is a notifiable disease. Currently, there is no registry for the documenting of GAS-related disease in Africa, despite the importance of GAS infections in this region.

## Methods

The African group A streptococcal infection registry (the AFROStrep Study) is a collaborative multicentre study of clinical, microbiological, epidemiological and molecular characteristics for GAS infection in Africa. The AFROStrep registry comprises two components: (1) active surveillance of GAS pharyngitis cases from sentinel primary care centres (noniGAS), and (2) passive surveillance of invasive GAS disease (iGAS) from microbiology laboratories. Isolates will also be subjected to DNA isolation to allow for characterisation by molecular methods and cryo-preservation for long-term storage.

## Discussion and Conclusion

Given that systematically collected data are essential for an effective disease-control programme, we have established the AFROStrep Registry as an essential first step towards understanding the prevalence of laboratoryconfirmed GAS disease in African countries. The AFROStrep study is a collaborative study that aims to establish the first registry and biorepository of laboratory-confirmed GAS isolates in Africa, with one of its main objectives being to collect comprehensive epidemiological, clinical, microbiological and molecular data for GAS infections on the continent. AFROStrep will serve as a platform for further investigations, including molecular characterisation of isolates in order to contribute to the growing body of knowledge informing vaccine development.

# Abstract: The prevalence and types of electrocardiographic abnormalities in patients with dilated cardiomyopathy at the Kenyatta National Hospital

**Authors:** Bernard Gituma, Elijah Ogola

**Affiliations:** University of Nairobi, Kenya; University of Nairobi, Kenya

## Background

Electrocardiographic abnormalities are common in dilated cardiomyopathy (DCM) and portend adverse prognosis, for example heart failure, embolic stroke and sudden death. We studied the pattern of electrocardiographic abnormalities in DCM at the Kenyatta National Hospital (KNH).

## Methods

This was a hospital-based, cross-sectional study of ECG abnormalities in patients with echocardiographic diagnosis of DCM, from the cardiac clinic and the medical wards in KNH, carried out between March and August 2013. Patients had a focused clinical evaluation and New York Heart Association (NYHA) class determined. Patients then had a standard resting 12-lead ECG recorded as per the British Cardiovascular Society clinical guidelines. The prevalence of ECG abnormalities was analysed using descriptive statistics and presented as proportions with 95% confidence interval (CI). The association between various ECG abnormalities and NYHA class was determined using the chi-square test.

## Results

In total, 216 patients were studied, mean age 53.3 years, and 52.3% were female. NYHA distribution was: I: 16.7%, II: 54.6%, III: 24.5% and IV: 3.7%. The prevalence of ECG abnormalities was 100% of the patients. The most common ECG abnormality was atrial fibrillation (31.5%), followed by left bundle branch block (LBBB) (30.6%). Other common abnormalities included first-degree AV block (17.6%), long QT interval (9.7%), second-degree AV block (9.3%), LVH (8.8%), third-degree AV block (3.7%), and SVT (2.3%). The prevalence of ECG abnormalities that were shown to have therapeutic and prognostic significance, i.e. atrial fibrillation, LBBB, secondand third-degree AV block, atrial flutter and SVT, was 83.6%. There was a statistically significant correlation between NYHA class and atrial fibrillation and LBBB.

## Conclusion

The high prevalence of ECG abnormalities makes ECG an important rule-out tool in diagnostic work-up, especially in resource-poor settings. There is a high prevalence of ECG abnormalities with implications for therapeutic interventions, such as anticoagulation in atrial fibrillation and resynchronisation therapy in LBBB. The ECG therefore is an important and cheap tool in the management of DCM.

# Abstract: An isolated single coronary ostium in a patient with dilated cardiomyopathy

**Authors:** Eren Gunertem, Serkan Mola, Ibrahim Erkengel, Ferhat Afsar, Salim Urcun

**Affiliations:** Department of Cardiovascular Surgery, Turkey Yuksek Ihtisas Education and Research Hospital, Cankaya, Ankara, Turkey; Department of Cardiovascular Surgery, Turkey Yuksek Ihtisas Education and Research Hospital, Cankaya, Ankara, Turkey; Department of Cardiovascular Surgery, Turkey Yuksek Ihtisas Education and Research Hospital, Cankaya, Ankara, Turkey; Department of Cardiovascular Surgery, Turkey Yuksek Ihtisas Education and Research Hospital, Cankaya, Ankara, Turkey; Department of Cardiovascular Surgery, Turkey Yuksek Ihtisas Education and Research Hospital, Cankaya, Ankara, Turkey

## Case report:

A 52-year-old woman was sent to an orthopaedist because of left arm pain over the previous four days. An electrocardiogram (ECG) was planned with the suspicion of cardiac pathology and it revealed a left bundle branch block (LBBB). The patient was referred us because of this. Her age was the only coronary risk factor. Physical examination revealed no pathological findings. Her blood pressure was 110/70 mmHg, and heart rate was regular with a mean of 70 beats/minute. Transthoracic echocardiography revealed depressed left ventricular ejection fraction of 40%, moderate mitral valve insufficiency, a dilated left ventricle and segmental wall motion abnormalities. In the light of these findings, the patient underwent coronary angiography.

During the angiography session, a single coronary artery originating from the right sinus of Valsalva was seen. There was no significant coronary artery stenosis. We interpreted these echocardiographic findings in favour of dilated cardiomyopathy and started her on an angiotensin converting enzyme inhibitor and furosemide therapy with acetylsalicylic acid. However six months later another transthoracic echocardiography was performed and showed decreased ejection fraction and enlarged heart chambers compatible with dilated cardiomyopathy

## Discussion

Patients with congenital coronary artery anomalies can be asymptomatic and these anomalies are only incidentally found during routine angiography. Coronary arteries originating from a single coronary ostium in the aorta are rare and occur in less than 0.03% of the general population. Hyrtl reported the first case of a single coronary artery in 1841. Single coronary artery anomalies are classified according to the site of origin, anatomical distribution on the ventricular surface, and according to its relationship with the ascending aorta and the pulmonary artery. Despite the relatively low incidence of atherosclerotic heart disease in young patients with coronary anomalies, there are some reports in the literature in which patients have congenital coronary artery anomalies with coronary artery disease. Karaagac and his colleagues presented a 63-year-old woman whose coronary angiography for typical chest pain revealed an isolated single coronary artery with a significant flow-limiting lesion in the right coronary artery that was successfully treated with percutaneous coronary intervention. The prognosis in patients with a single coronary artery varies according to the anatomical distribution. The left main coronary artery travels between the aorta and pulmonary arteries and this can cause sudden cardiac death in young people, especially during heavy physical exercise.

## Conclusion

We present a rare case of a patient who had a single coronary ostium in the right sinus of Valsalva with dilated cardiomyopathy. It should be recognised as potentially dangerous and may present unexpected results such as sudden death. We want to remind physicians of this rare condition that is often asymptomatic and detected incidentally.

# Abstract: A rapid ethnographic assessment approach to exploring patient and provider experience in rheumatic heart disease

**Authors:** Tayla Hermanus, Thembekile Shato, Mark Engel, Zühlke Liesl, Jantina De Vries

**Affiliations:** Department of Medicine, Faculty of Health Sciences, University of Cape Town, South Africa; Department of Paediatrics, Faculty of Health Sciences, University of Cape Town, South Africa; Department of Medicine, Faculty of Health Sciences, University of Cape Town, South Africa; Department of Paediatrics, Faculty of Health Sciences, University of Cape Town, South Africa; Department of Medicine, Faculty of Health Sciences, University of Cape Town, South Africa

## Introduction

Rheumatic heart disease (RHD), while negligible in high-income countries, remains prevalent in low- and middleincome countries and is associated with poverty, social inequality and poor access to healthcare. Understanding and addressing the lack of awareness and education is key in the prevention and control of RHD and its antecedent, acute rheumatic fever (ARF). Our goal was to develop an approach to generate qualitative data in different RHD-endemic communities. Here we report our approach to developing a rapid ethnographic assessment (REA) to examine the patient and provider experiences of RHD. While REA has not been used in RHD previously, it has achieved success in diverse studies in medicine and public health.

## Methods

Rapid assessment is an established and robust qualitative methodology that triangulates different qualitative research methods to rapidly elicit pertinent information. Its purpose is to attain a deep understanding of socio-economic and sociopolitical factors influencing poor health outcomes. Therefore REA bridges the gap between empirical research and implementation strategies whose success is dependent on direct engagement with local communities. For RHD, we have developed an REA protocol around two key areas of interest: health-seeking behaviour and adherence to secondary prophylaxis for patients with sore throat; and ARF and RHD and mapping experiences of patients and healthcare providers at the first point of care to identify gatekeepers to care.

## Conclusion

We have developed REA protocols for application in RHD, which we anticipate will ascertain the needs of diverse communities affected by ARF and RHD by obtaining information on patient and provider experience. The REA can now be used as a key component of a comprehensive needs-assessment tool. The REA protocol and instruments will be made publicly available for use by the RHD research community

# Abstract: Saving lost lives; the Namibian children’s heart project

**Authors:** Christopher Hugo-Hamman, Fenny Shidhika, H du Toit, A Brooks, S Vosloo

**Affiliations:** University of Cape Town, Christiaan Barnard Memorial Hospital; Ministry of Health and Social Services, Namibia; University of Cape Town, South Africa; Ministry of Health and Social Services, Namibia; Ministry of Health and Social Services, Namibia; University of Cape Town, South Africa; Christiaan Barnard Memorial Hospital, Cape Town, South Africa

## Introduction

Prior to 2008, there were no services for heart disease in Namibia. The aim of this project was to provide treatment for children with heart disease. Between September 2008 and December 2014, 148 patients seen by a single cardiologist in the Paediatric and Congenital Heart Clinic at Windhoek Central Hospital, were referred to the Christiaan Barnard Memorial Hospital in Cape Town, South Africa (1 500 km away), for intervention and/or surgery. Costs were covered by the Ministry of Health and Social Services, Namibia.

## Objectives

The primary aim of this study was to audit results obtained through this project. We describe clinical features and diagnosis at presentation, intervention or surgery received, outcome and complications associated, follow up over the six-year period and therefore, the medium-term impact of this project.

## Methods

This was a retrospective case series. Two data sources identified patients referred for surgery or intervention, first, records at the Windhoek Central Hospital, and second, hospital admission records at the Christiaan Barnard Memorial Hospital. Case notes were reviewed for diagnosis (echocardiogram, cardiac catheterisation), intervention or surgery, follow up and clinical outcome.

## Results

Of 272 identified as needing surgery or intervention 148 patients with age at presentation between thrree days and 23 years were referred to Cape Town. Of these, 49 had diagnostic and 13 interventional catheterisations. Four patients were inoperable, either through complexity or irreversible pulmonary hypertension. Cardiac surgery was performed in 112 patients, of which 16 were palliative procedures. Complex cardiac lesions, co-morbidities and late presentation contributed to post-operative morbidity. There were five early deaths (mortality rate 4.4%). Twenty-five have been lost to follow up.

## Conclusion

There is a heavy burden of congenital heart disease in Namibia, a low middle-income country without the capacity to operate on babies and small children and complex congenital heart disease. Opinion differs on whether countries with small populations (Namibia has 2.2 million people) should have independent units for paediatric cardiac surgery or should refer to larger regional centres. Nevertheless, this successful public– private partnership reports a large cohort of patients with a comparatively good clinical outcome.

# Abstract: Registry’s and research provide a remedy for public policy; rheumatic heart disease in Namibia

**Authors:** Christopher Hugo-Hamman, L Sikwaya, N Nzuza, A Awases, A Bock, N Forster

**Affiliations:** Windhoek Central Hospital; Ministry of Health and Social Services, Republic of Namibia; Christiaan Barnard Memorial Hospital and the University of Cape Town, South Africa; Windhoek Central Hospital; Ministry of Health and Social Services, Republic of Namibia; Windhoek Central Hospital; Ministry of Health and Social Services, Republic of Namibia; Windhoek Central Hospital; Ministry of Health and Social Services, Republic of Namibia; Windhoek Central Hospital; Ministry of Health and Social Services, Republic of Namibia; Windhoek Central Hospital; Ministry of Health and Social Services, Republic of Namibia

## Background

Although the prevalence is as high as 30/1 000 in children of seven to 17 years old, rheumatic heart disease (RHD) is much neglected in Africa. Prior to participation in the global registry (Remedy), the burden of RHD in Namibia was unknown. There were no data to inform public policy and no programme for primary or secondary prevention. We report data which informed the development of a national programme (ASAP) for prevention and control of RHD.

## Methods

The national registry of RHD was established at Windhoek Central Hospital in January 2010. From January 2010 to December 2014, 463 patients were enrolled in this prospective, national, hospital-based registry. Questionnaires document demographics, clinical presentation, complications, ECG and echocardiogram, and management at enrollment. Data are presented on the first 281 patients who were entered into Remedy.

## Results

The distribution of cases reflects regional population density; 61% were female and 39% male. Ninety-seven (34%) were children and 83% under 40 years. Thirty-two per cent were in NYHA III–IV and 48% in heart failure. Nineteen per cent had atrial fibrillation, 6% stroke and 13% previous surgery. Only 34% of patients were receiving secondary penicillin prophylaxis, of whom 35% oral Pen VK. Of patients needing anticoagulation, 39% were receiving warfarin, 38% were aware of the target INR and 73% had no INR analysis the preceding six months.

## Conclusion

The RHD burden is highly significant. Gaps identified in the organisation and delivery of care pointed to the need for a national programme for the prevention and control of RHD. Patients are seen late with advanced disease. Low numbers of patients over 50 years of age (4%) reflect high mortality rates. Low numbers on secondary penicillin prophylaxis and poor compliance with anticoagulation protocols reflect a lack of awareness of RHD among health workers. A national advisory committee (NAC) for RHD, the first in Africa, was established in May 2015. The NAC will now elaborate on a national programme ‘ASAP’. This example of ‘research to action’ emphasises profound public policy benefits from clinical science.

# Abstract: Right ventricular dysfunction in Nigerians with peripartum cardiomyopathy

**Authors:** Kamilu Karaye, Krister Lindmark, Michael Henein

**Affiliations:** Department of Medicine, Bayero University; and Aminu Kano Teaching Hospital, Kano, Nigeria; Department of Public Health and Clinical Medicine, Umea University, Sweden; Department of Public Health and Clinical Medicine, Umea University, Sweden

## Introduction

Right ventricular systolic dysfunction (RVSD) in relation to outcome in peripartum cardiomyopathy (PPCM) has not been previously well described. The present study therefore aimed to assess RVSD in a cohort of PPCM patients in Kano, Nigeria.

## Methods

This longitudinal study was carried out in three referral hospitals in Kano, Nigeria. Consecutive PPCM patients who had satisfied the inclusion criteria were recruited and followed up for 12 months. RVSD was defined as the presence of either tricuspid annular plane systolic excursion (TAPSE) < 16 mm or peak lateral wall systolic myocardial velocity (S’) by tissue Doppler imaging (TDI) of < 10 cm/s. Dilated RV and right atrium (RA) were defined as RV basal diameter (RVb) > 42 mm and RA end-systolic area (RAA) > 18 cm2 or RA length (RAL) > 53 mm, respectively.

## Results

A total of 54 patients were consecutively recruited over six months, but only 33 of them were followed up, of whom 12 died (36.4%), eight (66.7%) within the first six months, four were lost to follow up (12.1%) and the remaining 17 (51.5%) survived the one-year follow up. RAA, RAL and RVb reduced from 18.0 ± 7.9 cm2, 42.0 ± 10.5 mm and 48.4 ± 9.7 mm at baseline to 13.3 ± 4.1 cm2 (*p* = 0.022), 36.9 ± 9.7 mm (*p* = 0.08) and 44.7 ± 10.2 mm (*p* = 0.180) at one year, respectively. At baseline, 61.1% of patients had RVSD, which fell to 17.7% at the one-year follow up (*p* = 0.004), implying RV remodelling, but this was not associated with the use of specific treatments such as renin–angiotensin–aldosterone blockers. There was no relationship between RVSD and mortality in the regression models.

## Conclusion

RVSD was common in Nigerians with PPCM but improved during the first 12 months. It was neither related to medical treatment nor to mortality.

# Abstract: Contemporary aetiology, clinical characteristics and prognosis of adults with heart failure in Tanzania: the prospective Tanzania heart failure (TAHEF) study

**Authors:** Peter Kisenge, Abel Makubi

**Affiliations:** Cardiovascular Medicine, Muhimbili National Hospital, Cardiovascular Medicine Unit, School of Medicine; Muhimbili University of Health and Allied Sciences, Dar es Salaam, Tanzania

## Introduction

In developed countries, the prevalence and incidence of heart failure (HF) are 2–3 and 0.5%, respectively and increase with age. The aetiology, clinical characteristics, therapy and prognosis of this syndrome have been extensively studied in developed countries but considerably less so in developing countries, and HF is largely unexplored in sub-Saharan Africa (SSA). Aetiologies in SSA may be changing towards developedworld patterns but may also vary according to setting and geographical region. Co-morbidities, predictors of prognosis, and the beneficial effects and widespread use of neurohormonal antagonist drugs have been well described in the developed world but such data are limited in SSA, and there are concerns that mortality rates remain high. Therefore, the objective was to assess the contemporary aetiology, clinical characteristics, prognosis and predictors of prognosis of HF in Tanzania.

## Methods

This was a prospective, observational study in the Cardiovascular Centre of the Muhimbili National Hospital in Dar es Salaam, Tanzania. Patients were 18 years of age or older with HF defined by the Framingham criteria. The main outcome measure was all-cause mortality

## Results

Among 427 included patients, 217 (51%) were females and the mean age (standard deviation) was 55 years (17). Heart failure aetiologies included hypertension (45%), cardiomyopathy (28%), rheumatic heart disease (12%) and ischaemic heart disease (9%). Concurrent atrial fibrillation, clinically significant anaemia, diabetes, tuberculosis and HIV were found in 16, 12, 12, 3, and 2%, respectively, while warfarin was used in 3% of the patients. The mortality rate, 22.4 per 100 person-years of observation over a median follow up of seven months, was independently associated with the presence of atrial fibrillation, hazard ratio 3.4 (95% confidence interval 1.6–7.0); in-patient status 3.2 (1.5–6.8); anaemia 2.3 (1.2–4.5); pulmonary hypertension 2.1 (1.1–4.2) creatinine clearance 0.98 (0.97–1.00) and lack of formal education 2.3 (1.3–4.2).

## Conclusion

In HF in Tanzania, patients are younger than in the developed world, but aetiologies are becoming more similar, with hypertension becoming more prevalent and rheumatic heart disease less so. Predictors of mortality that are possible to intervene against are anaemia, atrial fibrillation and lack of education. Efforts directed toward diagnosing and treating hypertension, anaemia and atrial fibrillation, as well as increasing disease awareness, may reduce the incidence and mortality of HF in Tanzania.

# Abstract: Short-message services (SMS) as an educational tool used during pregnancy: a literature review

**Authors:** Kim Lamont, Karen Sliwa

**Affiliations:** Soweto Cardiovascular Research Unit (SOCRU), Faculty of Health Sciences, University of the Witwatersrand, Johannesburg, South Africa; Hatter Institute, University of Cape Town; SOCRU, Faculty of Health Sciences, University of the Witwatersrand, Johannesburg, South Africa

## Background

In many low- and middle-income countries, access to health information during pregnancy is very scarce. The rapid adoption of mobile phones in these countries has created new opportunities for disseminating health information.

## Objective

This article aims to review the existing information on the use of short-message services (SMS) as a feasible tool to transmit health education information.

## Methods

The PubMed, Cochrane library, EMBASE and Google scholar databases were searched for studies in which mobile phone SMS were used to promote health education during pregnancy. Studies of adult women from any setting who received SMS health education messages during their pregnancy were included, irrespective of study design.

## Results

The analysis of results followed a narrative synthesis approach, which is a textual approach of a synthesis of findings from multiple studies. The synthesis was developed manually based on the extraction of data. All studies demonstrated use or interest in SMS technology to facilitate a health information message. Gazmarian and colleagues (2013) assessed factors related to the enrolment process and reception of health tips via SMS in the USA. Cormick evaluated the attitude and willingness of pregnant women to receive health educational material via SMS in Argentina. In Zanzibar, the effect of SMS on skilled delivery rates and access to emergency healthcare was assessed. The effect SMS alerts had on hospital deliveries and SMS interventions had on facility use during pregnancy were assessed in Rwanda. Lastly MiQuit assessed the effects of tailored education through SMS on smoking cessation during pregnancy compared to non-tailored information through leaflets.

## Conclusion

This highlights the feasibility of utilising SMS technology to promote health education and promote behavioural change.

# Abstract: Linguistic utilisation for hypertension health education in primary healthcare, a South African health experience

**Authors:** Nokuthula Mafutha, Sophie Mogotlane

**Affiliations:** Tshwane University of Technology, Pretoria, South Africa; Tshwane University of Technology, Pretoria, South Africa

## Background

Health education is a means to empower patients with hypertension if they are to participate in its management. It is the cornerstone of patient management. Health education is a learning experience in patients, which fosters motivation, skills, and the confidence necessary to improve health and make behavioural changes. Health education uses communication processes such as individual discussions, and mass and group media to reach target groups. There are 11 official languages in South Africa, which pose a challenge to transmitting a clear message to patients, especially when adding other non-South African languages to the PHC.

## Methods

The research design was quantitative, descriptive and contextual in nature. The population/unit of analysis was health promoters and pamphlets. The sampling method was convenience. Data collection was done in 12 primary health clinics using an audio voice recorder and a camera to capturer pictures of posters that could not be taken away from the facility. Data analysis was done using quantitative content analysis and descriptive statistics.

## Results

The findings revealed that the following hypertension-related words were commonly used by healthcare promoters and also in pamphlets: blood pressure, headaches, salt, weight, comply, stop smoking, and exercise. These words were used without taking into consideration the literacy level of the patients, or even their preferred language.

## Conclusion

If self-care is the cornerstone for management of non-communicable diseases such as hypertension, health education and printed materials should be context-specific, and addressed in the language of the community and at the educational levels of the people in the area.

# Abstract: Prevalence, correlates and prognostic implications of anaemia and iron deficiency in Tanzanian patients with heart failure: a report from the TAHEF study

**Authors:** Abel Makubi, Camilla Hage, Johnson Lwakatare, Bruno Mmbando, Peter Kisenge, Lars Lund, Lars Ryden, Julie Makani

**Affiliations:** Muhimbili University of Health and Allied Sciences, Dar es Salaam, Tanzania; Muhimbili University of Health and Allied Sciences, Dar es Salaam, Tanzania; Muhimbili University of Health and Allied Sciences, Dar es Salaam, Tanzania; Muhimbili University of Health and Allied Sciences, Dar es Salaam, Tanzania; Muhimbili University of Health and Allied Sciences, Dar es Salaam, Tanzania; Karolinska institute, Sweden; Karolinska institute, Sweden; Muhimbili University of Health and Allied Sciences, Dar es Salaam, Tanzania

## Objective

To determine the prevalence, correlates and prognostic implications of anaemia and iron deficiency (ID) in patients with heart failure (HF) in Tanzania.

## Methods

This was a cross-sectional and prospective, observational study carried out at the cardiovascular centre of the Muhimbili National Hospital in Dar es Salaam, Tanzania. The patients were 18 years of age or older with HF, defined according to the Framingham criteria. The primary outcome was anaemia and the secondary outcome was a composite of hospitalisation for HF or all-cause mortality.

## Results

A total of 401 HF patients were included (median age 56 years, interquartile range 41.67 years; 51% females). The prevalence of anaemia was 57%. The overall prevalence of ID was 49%, distributed as 69% vs 21% in subjects with and with out anaemia (*p* < 0.001). Normocytic anaemia was seen in 18% of the patients while none had macrocytic anaemia. The risk of having anaemia was positively associated with residency outside Dar es Salaam [OR 1.72 (95% CI 1.02–2.89); *p* = 0.038], atrial fibrillation [4.12 (1.60–10.61); *p* = 0.003], left ventricular ejection fraction < 45% [2.70 (1.5 –4.67); *p* < 0.001] and negatively associated with creatinine clearance (ORs per unit decrease) [0.98 (0.97–0.99); *p* = 0.012] and total cholesterol level [0.78 (0.63–0.98); *p* = 0.029].

The one-year survival free from severe HF outcome was 70%. The presence of ID anaemia increased the likelihood for a HF event [HR 2.67; 95% CI 1.39–5.07; *p* = 0.003], while anaemia without ID did not influence the risk.

## Conclusion

Iron-deficiency anaemia was common in Tanzanian patients with HF and was independently associated with the risk for hospitalisation or death.

# Abstract: Rationale and design of the role of oral iron in patients with heart failure and iron deficiency in Tanzania: the prospective Tanzania heart failure pilot oral iron clinical trial (TaHeFII)

**Authors:** Abel Makubi, Lars Lund, Julie Makani

**Affiliations:** Muhimbili University of Health and Allied Sciences, Dar es Salaam, Tanzania; Karolinska institute Sweden; Karolinska institute Sweden

## Introduction

In patients with chronic heart failure (HF), iron deficiency is common and associated with increased morbidity and mortality rates, independent of anaemia. Intravenous (iv) iron does improve quality of life and may reduce HF hospitalisation. However, iv iron is expensive and administration to large populations of HF patient is therefore not infeasible, particularly in countries with limited healthcare resources.

Oral iron is an established therapy for treating iron deficiency but has not been widely tested in HF patients. Oral iron remains promising in resource-limited settings because (1) it is easily available, (2) easy to administer to patients, (3) newer ferrous sulphate preparations may be better absorbed than older ferrous sucrose, (4) the pathophysiology of iron deficiency may differ geographically, and (5) oral iron is inexpensive. In view of this, we will assess whether addition of oral iron therapy to the conventional therapy improves haematological, myocardial, biochemical and clinical parameters in adult patients with HF and iron deficiency. This is a pilot study for a possible future large, randomised clinical trial.

## Methods

This is a single-centre, prospective, open-label, uncontrolled clinical trial. A total of 150 individuals with stable chronic HF and iron deficiency (with or without anaemia) are being piloted at Muhimbili cardiovascular centre in Dar es Salaam, for the period of eight months (January to August 2015.) Eligible patients will receive a fixed-dose oral iron sulphate therapy (200 mg), given three times a day for three months. The endpoint will be improvement in mean serum ferritin, left ventricular size and ejection fraction, six-minute walk distance, and NT-proBNP and haemoglobin levels at the end of three months, compared to baseline levels.-

## Conclusion

Should this pilot study become successful, it will pave way to a full randomised, controlled trial, which might determine the beneficial effect of oral iron in HF, thus improving the prognosis of this population.

# Abstract: Role of technology in creating rheumatic heart disease awareness among school-going children in Kenya

**Authors:** Duncan Matheka, Mellany Murgor, Gary Selnow

**Affiliations:** Machakos Level 5 Hospital, Kenya; University of Nairobi, Kenya; WiRED International

## Introduction

Rheumatic heart disease (RHD) is the most common cardiovascular disease in Kenya and mainly affects school-going children. As a preventable disease, its incidence may be significantly reduced by educating the community on preventative measures. Educating children is crucial in combating the disease since they are especially vulnerable to streptococcal infection. The role of innovative training approaches (technology based) among school-going children remains unverified.

## Objective

The current project therefore sought to train schoolgoing children on RHD using an interactive digital module from WiRED International, a US-based non-profit organisation working in Kenya.

## Methods

The module offered simplified animated presentations linking sore throat, rheumatic fever and RHD, as well as ways of their prevention. The module also introduced questions throughout the presentation and provided instant feedback to reinforce key concepts.

Upper primary school pupils from two schools were randomly assigned into control (*n* = 100) and experimental (*n* = 100) groups. The experimental group was trained using the module, while the control group did not have any teaching. Both groups then answered 23 multiple-choice questions (MCQs). During a follow-up visit one week later, the students were re-administered with the same final examination. The results were analysed using SPSS version 16.0.

## Results

The mean age of the pupils was 12.71 years. On the first test, the experimental group had higher average scores compared to the control group (16.3 ± 2.5 vs 10.5 ± 2.3 marks; *p* < 0.001). The follow-up test results were 15.7 ± 2.7 for experimental and 10.4 ± 2.4 marks for the control, *p* < 0.001. Age, class level or gender did not affect their performance.

## Conclusion

The use of interactive digital modules to train school-going children on RHD increases knowledge, awareness and is feasible, efficacious and sustainable. This approach is beneficial, and could potentially reduce the toll of RHD if tailored to the specific learning needs of the children and applied more widely.

# Abstract: Rheumatic heart disease patient support clubs: the Kenyan experience

**Authors:** Duncan Matheka, Mellany Murgor, Laura Musambayi, Kate Armstrong, Christine Jowi

**Affiliations:** Machakos Level 5 Hospital, Kenya; University of Nairobi, Kenya; University of Nairobi, Kenya; Caring and Living as Neighbours (CLAN), Australia; University of Nairobi, Kenya

## Background

Rheumatic heart disease (RHD) is the most common heart disease among children in Kenya. It is a chronic disease that primarily affects poor populations that cannot afford healthcare services. These patients often despair and are lost from follow up until advanced stages of the disease, usually resulting in high mortality rates. There is therefore an urgent need to promote holistic healthcare in Kenya to alleviate this burden. We hereby share our experience adapting a personcentred support club model that has been effectively used by CLAN (Caring and Living as Neighbours, an Australian NGO) to improve quality of life for children and adolescents living with a range of chronic health conditions in low-income settings in the Asia–Pacific region.

## Methods

Planning for the Kenyan RHD family support club utilised CLAN’s rights-based, community development framework for action, and focused multi-sectoral, internationally collaborative action on five key pillars: (1) affordable access to medicine (monthly penicillin) and equipment (echocardiography); (2) education (of children with RHD and their families, healthcare professionals, policy makers and the national and international community), research and advocacy; (3) optimal medical management (through primary, secondary and tertiary prevention); (4) establishment and development of Kenyan RHD family support clubs; (5) reducing financial burdens on and promoting financial independence of families living with RHD.

## Results

Successful engagement of a broad network of national and international multi-sectoral organisations around the Kenyan RHD support club launch of 8 March 2014 established the Kenyan RHD community as a visual hub for ongoing person-centred healthcare in the country. Support clubs offer material, moral and psychological support within a cost-effective, strategic, sustainable health system-strengthening, multidisciplinary approach.

## Conclusion

Support clubs as modelled in the Asia–Pacific region have potential for empowering families and communities in Kenya to engage with a broad range of partners around a united vision of improved quality of life for children who are living with RHD in Kenya.

# Abstract: Choice of anti-hypertensives among physicians and its impact on blood pressure control in Nigerians with hypertension

**Authors:** Muhammad Sani Mijinyawa, Hamza Muhammad, Hadiza Saidu, Balarabe Sulaiman

**Affiliations:** Department of Medicine, Bayero University, Kano; and Aminu Kano Teaching Hospital, Kano; Department of Medicine, Bayero University, Kano, Nigeria; Department of Medicine, Bayero University, Kano, Nigeria; Department of Medicine, Aminu Kano Teaching Hospital, Kano, Nigeria

## Introduction

Hypertension, if untreated or uncontrolled, leads to damage of vital organs such as the brain, heart and the kidneys, among others. These complications have been shown to be severer in black Africans. The benefit of treatment has been repeatedly demonstrated by many studies. Therefore many guidelines have been produced by relevant bodies in different countries in order to assist physicians in making the right choices for blood pressure (BP) control. Most of these bodies produce the guidelines based on the peculiarities of hypertension in their respective population. Several reports have shown how different hypertension is in black Africans, but there is no published guideline for its treatment in this population.

## Methods

This was a survey of known hypertensives who were on follow-up visits. Their prescriptions were assessed for drug name, class and number. Their blood pressures at that visit were also recorded. The prevalence of single therapy and combination therapy were determined. The percentage of BP control as well as the prescribed drugs in each group were also obtained. Compliance with the AHA-recommended two-drug combination was determined.

## Results

Those on a single agent were 13.11%, of whom 51.16% were controlled; 86.89% were on various combinations of two or more drugs, of whom 31.21% had controlled BP. BP control in those on two drugs was better than in those with more than two drugs (*p* = 0.0027).

ACEIs were the commonest used drug, either as a single agent (55.81%) or as a two-drug combination, as seen in 54.83% of the subjects on a two-drug combination. Fourteen different two-drug combinations were identified, with the best control seen in an ARB + diuretic, ACEI + diuretic and CCB + diuretic. The least control was observed in the ACEI + CCB group. Compliance with AHA recommendation was good but 7.7% were still in the unacceptable group, while another 7.7% were unclassified.

## Conclusion

Although there is better control in diuretic-based combinations, fewer diuretics are used as single agents. Despite fair compliance with AHA recommendations on drug combinations, overall BP control is still a problem, which calls for a revisit of these recommendations in African populations.

# Abstract: Group A streptococcal carriage in children residing in African countries

**Authors:** Annesinah Moloi, AH Moloi, L Abdullahi, DD Barth, ME Engel

**Affiliations:** University of Cape Town, Groote Schuur Hospital, Cape Town, South Africa; University of Cape Town, Groote Schuur Hospital, Cape Town, South Africa; University of Cape Town, Groote Schuur Hospital, Cape Town, South Africa; University of Cape Town, Groote Schuur Hospital, Cape Town, South Africa; University of Cape Town, Groote Schuur Hospital, Cape Town, South Africa

## Background

Asymptomatic children can be a major reservoir of pharyngeal Group A *Streptococcus* (GAS), with reported figures ranging from < 10% to > 20% in developing countries. There is a need to document GAS carriage in school children of all ages, which, together with the molecular characterisation (M-typing) of strains harboured in the pharynx of carriers, will help to ascertain the extent to which disease strains are prevalent among carriers. This background information would serve to assist healthcare providers in diagnosing symptomatic GAS pharyngitis, as well as potentially contributing to the development of a GAS vaccine. Currently, data on prevalence of GAS carriage and M-type distribution in African countries are largely scant. We therefore undertook to perform a systematic review to determine the prevalence and type distribution of asymptomatic streptococcus carriage in children aged five to 15 years, residing in African countries.

## Methods

We conducted a comprehensive literature search among a number of databases, using an African search filter to identify GAS prevalence studies that report on children between the ages of five and 15 years who reside in African countries. Electronic searches were complemented by a hand-search performed on reference lists of potentially included studies. The search was not limited by year of publication and language. Two evaluators independently reviewed, rated and abstracted data from each article. Prevalence estimates were pooled in a meta-analysis and stratified according to region and study design, using Stata®. Specifically, we applied the random-effects metaprop routine to aggregate prevalence estimates and account for between-study variability in calculating the overall pooled estimates and 95% CI for prevalence of GAS carriage.

## Results

Of the 1 665 articles retrieved, 16 studies incorporating cross-sectional and longitudinal study designs met with the inclusion criteria. The pooled prevalence of GAS carriage was 8% [95% confidence interval (CI), 5–10%] with pooled prevalences for cross-sectional studies equal to 8% (95% CI, 5–11%) and longitudinal studies, 9% (95% CI, 1–24%). Regional pooled rates were similar across East, Central and Southern Africa of between 7 and 8%, while countries within northern Africa had a pooled estimate of 14% (95% CI, 3–30%). Western Africa had the lowest pooled estimate of 2% (95% CI, 1–2%). Only one study reported data on molecular characterisation, of which 11/13 (85%) emm-types were included among the putative 30-valent vaccine currently under development.

## Conclusion

This study revealed a pooled GAS carriage estimate of 8% among African school children. Regional differences in carriages were largely minimal except for West and North Africa with prevalences of 2 and 14%, respectively. There is a dearth of data on molecular strain information, therefore emphasising the need for further studies.

# Abstract: Central arterial stiffness in Zambian normotensive and hypertensive participants

**Authors:** Festus Mushabati, Fastone M Goma, Lukubi Lwiindi, Nathan J Siulapwa, Sete Siziya

**Affiliations:** Copperbelt University, School of Medicine, Ndola, Zambia; University of Zambia, Zambia; Lusaka Apex Medical University, Zambia; Copperbelt University, School of Medicine, Ndola, Zambia;; Copperbelt University, School of Medicine, Ndola, Zambia;

## Background

Central arterial stiffness is a strong and independent predictor of cardiovascular events and mortality, including hypertension, in any given population. A few studies have found that being black could be associated with elevated arterial stiffness, thereby accounting for the high prevalence of hypertension in these populations. The present study sought to determine and compare central arterial stiffness using carotid femoral PWV (cfPWV) in a population of Zambian normotensive (NT) and hypertensive (HT) participants [both treated (HTC) and untreated hypertensives (HTN)] between 30 and 65 years of age.

## Methods

cfPWV was measured in 146 participants. A Complior® analysis device (Version 1.9 Beta 2013; ALAM Medical, France) was used. Superficial pulses were accessed non-invasively over the carotid–femoral segment.

## Results

The cfPWV values in the HTN participants (*n* = 23) were significantly higher than in the NT (*n* = 64) participants (11.4 ± 4.2 m/s vs 9.1 ± 3.2 m/s, p = 0.009). In the HTC participants (*n* = 59), their cfPWV values (10.4± 5.6 m/s) tended to approach those in the HTN participants, but with no statistical differences between them (*p* > 0.500). Furthermore, the mean cfPWV found in the NT participants was considerably higher than any found in previous studies, especially in white-skinned populations. Carotid femoral PWV did not show significant age-related increase in all three blood pressure categories (*r*^2^ < 0.03, *p* > 0.100).

## Conclusion

These findings show that central elastic arteries of HT participants, regardless of being on anti-hypertensive therapy or not, were stiffer and therefore less compliant than those in the NT participants, adding to their burden of hypertension. Regrettably, the HTC participants showed poor BP control, inferred from their considerably high cfPWV values that tended to approach the stiffness values of the untreated group.

# Abstract: Benzathine penicillin adherence for secondary prophylaxis among patients affected with rheumatic heart disease attending the Mulago Hospital

**Authors:** Charles Musoke, Charles Kiiza Mondo, Emmy Okello, Wanzhu Zhang, Barbara Kakande, Wilson Nyakoojo, Juergen Freers

**Affiliations:** Mulago National Referral Hospital; and Uganda Heart Institute, Kampala, Uganda; Mulago National Referral Hospital; and Uganda Heart Institute, Kampala, Uganda; Uganda Heart Institute, Kampala, Uganda; Uganda Heart Institute, Kampala, Uganda; Uganda Heart Institute, Kampala, Uganda; Uganda Heart Institute, Kampala, Uganda; Makerere University, Kampala, Uganda

## Introduction

Rheumatic heart disease (RHD) frequently occurs following recurrent episodes of acute rheumatic fever (ARF). Benzathine penicillin (benzapen) is the most effective method for secondary prophylaxis against ARF, whose efficacy largely depends on adherence to treatment. Various factors determine adherence to therapy but there are no data regarding current use of benzapen in patients with RHD attending the Mulago Hospital. The study aims were (1) to determine the levels of adherence with benzapen prophylaxis among RHD patients at the Mulago Hospital, and (2) establish the patient factors associated with adherence and the reasons for missing monthly benzapen injections.

## Methods

This was a longitudinal, observational study carried out at Mulago Hospital cardiac clinics over a period of 10 months; 95 consecutive RHD patients who satisfied the inclusion criteria were recruited over a period of four months and followed up for six months. Data on demographic characteristics and disease status were collected by means of a standardised questionnaire and a card to document the injections of benzapen received.

## Results

Most participants were female (75; 78.9%). The age range was five to 55 years with a median of 28 years. The mean age was 28.1 years (SD 12.2). The highest education level was primary school for most patients (44, 46.3%) with eight (8.4%) of the patients being illiterate. Most were in either NYHA stage II (39, 41.1%) or III (32, 33.7%).

Benzathine penicillin adherence: at least 44 (54%) adhered to the monthly benzapen prophylaxis, with adherence rates ≥ 80%; 38 (46%) patients were classified as non-adherent to the monthly benzapen, with rates less than 80%. The mean adherence level was 70.12% (SD 29.25) and the median level was 83.30%.

Factors associated with adherence: higher education status, and residing near a health facility favoured high adherence. Painful benzapen injection was the main reason for missed doses.

## Conclusion

The level of non-adherence was significantly high (46%).The painful nature of the benzapen injections and lack of transport money to travel to the health centre were the main reasons for non-adherence among RHD patients in the Mulago Hospital.

# Abstract: Teachers’ knowledge and attitudes related to rheumatic heart disease in Zambia

**Authors:** John Musuku, Joyce Chipili, Aidan Long, Brigitta Tadmor, Jonathan Spector

**Affiliations:** University Teaching Hospital, Lusaka;; University Teaching Hospital, Lusaka;; Massachusetts General Hospital, USA; Novartis Institutes of Biomedical Research; Novartis Institutes of Biomedical Research

## Introduction

Rheumatic heart disease (RHD) is a major public health problem in Africa affecting 1–5% of school children. Community and school involvement is increasingly recognised to be an essential component of national strategies to control RHD, but very little is known about teachers’ knowledge and attitudes about the disease. As part of a public–private partnership to combat RHD in Zambia, school-based screening of up to 10 000 school children will be conducted in Lusaka for the first time, using portable echocardiography. In preparation, we sought to characterise teachers’ knowledge of RHD, explore their willingness to participate in RHD screening programmes at their schools, and assess their general interest in participating in advocacy efforts related to RHD.

## Methods

A workshop was conducted for primary and secondary school teachers in Lusaka in February 2014. The curriculum was developed from educational materials produced by the World Health Organisation and World Heart Federation, and included a focus group session and written attitude survey. Participants also completed an eight-item multiple-choice questionnaire before and after the course to evaluate basic knowledge about RHD. Mean test scores were compared using paired Wilcoxon signed rank sum testing (SOFA software, version 1.3.4).

## Results

Fifty-three teachers from more than 45 schools participated. Most were female. All but threre had been teachers for at least five years and 26% had taught for more than 15 years. Approximately half of the teachers also served as their school’s health officer. Only 55% had ever heard of RHD before the workshop, and 24% reported that they had known a student with RHD. Forty-nine per cent of teachers were unaware that RHD is caused by a bacterial infection of the throat and few (less than 25%) knew that children with RHD require continual antibiotics to prevent progression of their heart disease. Pre–post knowledge scores improved from 3.8/8 (SD 0.9) to 5.9/8 (SD 1.2; *p* < 0.001). In the focus group discussion, teachers were overwhelmingly eager to help facilitate RHD screening programmes at their schools. They also expressed interest in learning more about how to prevent and treat RHD in order to help keep their students healthy.

## Conclusion

Teachers’ baseline awareness of RHD was poor and few had first-hand exposure to students with RHD despite the high prevalence of the disease in Africa. Notwithstanding, teachers were eager to learn about RHD and they demonstrated significantly improved knowledge after the workshop. Teachers in Lusaka appear poised to be vital partners in school-based screening programmes and may also play important roles in long-term efforts to control RHD in Zambia.

# Abstract: Improving access to secondary prevention for rheumatic heart disease: knowledge and skills training to address fear of anaphylactic penicillin allergy in Zambia

**Authors:** John Musuku, Aidan Long, Jonathan Spector, Brigitta Tadmor, Gloria Habanyama

**Affiliations:** University Teaching Hospital, Lusaka;; Massachusetts General Hospital, USA; Novartis Institutes of Biomedical Research; Novartis Institutes of Biomedical Research; University Teaching Hospital, Lusaka

## Introduction

Identifying barriers to penicillin administration is vital to the success of secondary prevention programmes for rheumatic heart disease (RHD). In Zambia we discovered that fear of anaphylactic penicillin allergy among health workers was preventing their adherence to standard treatment guidelines, which negatively impacted on patient care. To address this concern, we designed and implemented a city-wide penicillin allergy workshop. This is, to our knowledge, the first report of such a workshop in Africa.

## Objective

We sought to characterise changes in knowledge and skills after implementation of a novel penicillin allergy training workshop in Lusaka, Zambia, and to determine trainees’ perceptions of the utility of the course.

## Methods

As part of a large-scale public–private partnership to combat RHD in Zambia, a two-day penicillin allergy workshop was conducted in Lusaka in July 2013. The curriculum was developed according to evidence-based guidelines from the World Allergy Organisation, and included interactive didactic sessions, peer-to-peer learning, and a skills session focused on anaphylaxis management. Trainees completed a 10-item multiple-choice questionnaire before and after the course and were graded on a standardised five-point scale during pre- and post-training skills sessions. Mean test scores were compared using paired Wilcoxon signed rank sum testing (SOFA software, version 1.3.4).

## Results

Twenty-nine health workers (mostly nurses and doctors) from 20 district clinics and the University Teaching Hospital participated. Knowledge scores improved from 7.8/10 (SD 1.6) to 9.8/10 (SD 0.4; *p* < 0.001), and skill scores improved from 2.5/5 (SD 1.4) to 4.8/5 (SD 0.4; *p* < 0.001). In anonymous postcourse evaluations, all participants reported that their clinical practice would change as a result of the course.

## Conclusion

Significantly improved knowledge and skills were documented after conduct of a penicillin allergy workshop in Zambia, and all trainees reported the course to be highly relevant to their clinical practice. It is recommended that clinicians, policymakers, and other stakeholders that work in similar contexts determine whether fear of penicillin allergy is a contributing cause of failed secondary prevention in their programs and, if so, to address this concern in order to safeguard essential medical care for patients with RHD.

# Abstract: Characteristics and treatment outcomes of patients with and without pulmonary hypertension who underwent cardiac surgery at Muhimbili cardiac unit from 2008 to 2012

**Authors:** Reuben Mutagaywa

**Affiliations:** Muhimbili University College of Health and Allied Sciences, Dar es Salaam, Tanzania

## Introduction

Pulmonary hypertension (PHT) can complicate rheumatic heart disease (RHD) and congenital heart disease (CHD). Early intervention is the mainstay to avoid PHT and its complications. Here we report the outcomes of patients with and without PHT admitted for cardiac surgery at Muhimbili National Hospital (MNH).

## Methods

A total of 453 patients were assessed retrospectively from patients’ files, wards and theatre records. Ethical clearance was obtained. Data were recorded and filled in on a structured questionnaire. Analyses were done using mean or median, counts or percentages, the chi-square test and Kaplan–Meir survival curve; *p*-value < 0.05 was considered statistically significant.

## Results

A total of 330 patients underwent surgical operations in which 212 had RHD, 91 had CHD, 14 had both RHD and CHD, and 13 had other forms of cardiac disease. Among the operated patients, data on PHT were available in 259 (78.5%), where 178 (68.7%) had PHT. In the RHD group, PHT was present in 150 (82.0%) of patients, in whom 31.7% had mild PHT, 30.6% moderate, and 19.7% severe PHT. In the CHD group, PHT was present in 18 (32.1%) patients, 14.3% had mild PHT, 16.1% moderate, and 1.8% severe PHT.

At the end of the patients’ files review from the day of surgery to the day each patient was last seen, 47 (18.1%) patients had died, and 80.9% of them had had PHT. In patients who died from RHD, 26 (66.7%) had died within 90 days (early mortality) and 88.5 % of these had PHT. All of the deaths in the CHD group were early mortalities and all had had PHT. Moreover, patients with PHT stayed longer in ICU postoperatively versus patients without PHT. Kaplan–Meir analysis demonstrated an 85% one-year survival. When stratified by PHT the one-year survival was 90% in patients without PHT and 80% in patients with PHT (log-rank *p* = 0.06).

## Conclusion

The prevalence of PHT in patients undergoing cardiac surgery at MNH was high, especially in those with RHD. PHT was associated with early postoperative mortality. These findings call for intense care during the early postoperative period and further studies are required to look for other factors causing mortality.

# Abstract: Balloon mitral valvuloplasty in Kenya

**Authors:** Loice Mutai, Christine Jowi

**Affiliations:** Department of Paediatric Cardiology, the Mater Hospital; and University of Nairobi, Kenya; Department of Paediatric Cardiology, the Mater Hospital; and University of Nairobi, Kenya

## Introduction

A retrospective study was done to analyse transcatheter treatment of severe mitral valve stenosis (MS) in rheumatic heart disease at the Mater Hospital. The patient profile, outcome and follow up were assessed. The period was over 19 years since the treatment became available, to date.

## Methods

A retrospective study was done at the Mater Hospital. Data were obtained from 1996 until April 2015 from the catheterisation laboratory procedure book and individual patient files by the investigator and research assistants. The study population was rheumatic heart disease patients with severe mitral stenosis who had balloon mitral valvuloplasty (BMV) at the hospital.

## Results

There were 272 patients treated in the catheterisation laboratory for over 19 years to date; 32% were 10 to 15 years old, 20% were 16 to 20, 14% were 21 to 25, and 32% were 26 to 50 years old. The most commonly used method of BMV was the multitrack technique. The majority of patients were lost to follow up after two visits to the clinic. This is usually within the first year of treatment. Only two had over 10 years of follow up.

## Conclusion

Balloon mitral valvuloplasty is an efficient way to treat MS patients. It has a short hospital stay of between three and five days’ duration. Follow up at the hospital is poor. Without long-term data it is difficult to tell whether BMV is an effective treatment of patients with severe MS. It should be possible to set up a data base which is updated each time patients are seen. Even reviews at private offices could be channelled on e-mail to update this data base.

# Abstract: Congenital heart block in a Ugandan infant

**Authors:** Judith Namuyonga, Peter Lwabi, Sulaiman Lubega

**Affiliations:** Uganda Cardiac Society, Uganda Heart Institute, Mulago Hospital, Kampala, Uganda; Uganda Cardiac Society, Uganda Heart Institute, Mulago Hospital, Kampala, Uganda; Uganda Cardiac Society, Uganda Heart Institute, Mulago Hospital, Kampala, Uganda

## Introduction

Congenital heart block is a rare, fatal disorder. It occurs among one in 22 000 live births and has been associated with maternal lupus. Most studies have been published as case reports in developed countries.

## Case report

A case of a seven-month-old infant was referred to our facility, the Uganda Heart Institute, a super-specialised unit and the main referral for patients with heart conditions within Mulago National Referral Hospital in Kampala, Uganda. The baby presented with index generalised seizures with eye rolling, fatigue upon breastfeeding and apnoea. The baby was weak and had delayed milestones compared to her siblings. She was a clinically sick infant, small for age (4.8 kg), with oxygen saturation of 99%, and no oedema. She had severe bradycardia; the pulse rate was 39 beats per minute, with normal volume and synchronous with other pulses. The baby had respiratory distress, with a rate of 60 breaths/minute, and no basal crepitations.

Two-dimensional echocardiography revealed dilated heart chambers, non-compaction of the left ventricle with severe systolic dysfunction. The electrocardiogram showed severe bradycardia with third-degree heart block. A haemogram revealed features of iron deficiency anaemia (Hb 8.1 g/dl, MCV 59 fl), and the white blood cell count was 12.8 × 103.

Renal function and electrolytes were normal, and the maternal anti-nuclear factor, (ANCA) was non-reactive. Treatment given was intravenous dopamine 3 mg/kg renal protective dose, captopril and furosemide while the baby awaited pacemaker implantation. A permanent pacemaker (epicardial pacemaker generator) was implanted in the left hypochondrium. Six months after the pacemaker was implanted, the baby had gained weight, heart rate was 100 bpm, blood pressure 114/60 mmHg and the ECG demonstrated good capture.

## Conclusion

The ECG is significant in diagnosing children who present with severe left ventricular systolic dysfunction. This is the first case report on successful pacemaker implantation among children with congenital heart block in sub Saharan Africa.

# Abstract: Hypertensive heart failure in Abeokuta, Nigeria. Clinical characteristics and outcome: lessons from the Abeokuta heart failure registry

**Authors:** Okechukwu S Ogah, Ayodele Falase, Simon Stewart, Joshua O Akinyemi, Karen Sliwa

**Affiliations:** Division of Cardiology, Department of Medicine, University College Hospital, Ibadan, Nigeria;; Division of Cardiology, Department of Medicine, University College Hospital, Ibadan, Nigeria;; Mary MacKillop Institute for Health Research, Australian Catholic University, Melbourne, Australia; Department of Epidemiology and Medical Statistics, College of Medicine, University of Ibadan, Nigeria; Hatter Institute for Cardiovascular Research in Africa; and Institute of Infectious Disease and Molecular Medicine, Faculty of Health Sciences, University of Cape Town, Cape Town, South Africa

## Introduction

Hypertension is the commonest cardiovascular disease and the major risk factor for heart disease in Nigeria. There is paucity of information on hypertensive heart failure from Africa’s most populous country, which necessitated this study.

## Methods

Data from the Abeokuta Heart Failure Registry were used to determine the clinical characteristics, mode of treatment as well as intra-hospital outcome of hypertensive heart failure (HT-HF) patients in Abeokuta, south-west Nigeria. Eligible subjects were hypertensive patients with new-onset HF or those with decompensated HF. Standardised data of demographics and clinical profile, 12-lead ECG, echocardiography, treatment and outcome were obtained.

## Results

Three hundred and twenty subjects with HT-HF (17.8% with acute decompensated HF) were consecutively studied comprising 184 (57.5%) men and 136 (42.5%) women aged 58.4 ± 12.4 and 60.6 ± 14.5 years, respectively. The majority (n = 290, 90.6%) of subjects were known hypertensives and presented with a BP of 144 ± 32/91 ± 21 mmHg and heart rate of 96 ± 19 beats/min. Most (80%) presented in NYHA class III or IV and around one-third (35%) had preserved systolic function. Overall, the women had a higher body mass index than the men. Alternatively, cigarette smoking and alcohol consumption were significantly higher in men. A similar proportion had co-morbid type 2 diabetes (12.2%) and/or atrial fibrillation (12.8%). The majority were prescribed ACEI/ARB therapy (319, 99.1%) and/ or spironolactone (81.3%).

Median hospital stay was nine days (IQR 5–21) while intra-hospital mortality was 3.4%, with minimal gender-based differences. The 30-, 90- and 180-day mortality rates were 0.9% (95% CI: 0.2–3.5), 3.5% (95% CI: 1.7–7.3) and 11.7% (95% CI: 7.8–17.5), respectively. In a multiple logistic regression analysis, only serum creatinine was the independent predictor of mortality at 180 days (adjusted OR = 1.76, 95% CI: 1.17-2.64)

## Conclusion

Hypertension, as a highly preventable condition, is the commonest aetiological risk factor for heart failure in Nigeria. Most subjects present in their prime of life with severe heart failure and secondary valvular dysfunction and significant in-hospital mortality

# Abstract: Economic cost of heart failure in Nigeria: data from the Abeokuta heart failure registry

**Authors:** Okechukwu S Ogah, Simon Stewart, Obinna E Onwujekwe, Ayodele Falase, Saheed Adebayo, Taiwo O Olunuga, Karen Sliwa

**Affiliations:** Division of Cardiology, Department of Medicine, University College Hospital, Ibadan, Nigeria; Mary MacKillop Institute for Health Research, NHMRC CRE to Reduce Inequality in Heart Disease, Australian Catholic University; Department of Health Administration and Management, Faculty of Health Sciences and Technology, University of Nigeria, Enugu Campus, Enugu State, Nigeria; Division of Cardiology, Department of Medicine, University College Hospital, Ibadan, Nigeria; Department of Medicine, Federal Medical Centre, Abeokuta, Nigeria; Department of Medicine, Federal Medical Centre, Abeokuta, Nigeria; Hatter Institute for Cardiovascular Research in Africa and Institute of Infectious Disease; and Molecular Medicine, Faculty of Health Sciences, University of Cape Town, Cape Town, South Africa

## Introduction

Heart failure (HF) is a deadly, disabling and often costly syndrome worldwide. Unfortunately, there is a paucity of data describing its economic impact in sub-Saharan Africa; a region in which the number of relatively younger cases will inevitably rise.

## Methods

Heath economic data were extracted from a prospective HF registry in a tertiary hospital situated in Abeokuta, south-west Nigeria. Out-patient and in-patient costs were computed from a representative cohort of 239 HF cases including personnel, diagnostic and treatment resources used for their management over a 12-month period. Indirect costs were also calculated. The annual cost per person was then calculated.

## Results

Mean age of the cohort was 58.0 ± 15.1 years and 53.1% were men. The total computed cost of care for HF in Abeokuta was 76 288 845 Nigerian Naira (US$508 595), translating to 319 200 Naira (US$2 128) per patient per year. The total cost of in-patient care (46% of total healthcare expenditure) was estimated at 34 996 477 Naira (about US$301 230). This comprised 17 899 977 Naira (50.9%; US$114 600) and 17 806 500 Naira (49.1%; US$118 710) for direct and indirect costs, respectively. Out-patient cost was estimated as 41 292 368 Naira (US$275282). The relatively high cost of out-patient care was largely due to cost of transportation for monthly followup visits. Payments were mostly made through out-of-pocket spending.

## Conclusion

The economic burden of HF in Nigeria is particularly high, considering the relatively young age of affected cases, a minimum wage of 18 000 Naira (US$120) per month and a considerable component of out-of-pocket spending for those affected. Health reforms designed to mitigate the individual-tosocietal burden imposed by the syndrome are required.

# Abstract: Cardio metabolic risk factors in ambulatory patients attending primary healthcare facilities in Nairobi, Kenya

**Authors:** Elijah Nyainda Ogola, Loice Achieng, Mark Joshi, Ken Mutai

**Affiliations:** University of Nairobi, Nairobi, Kenya; University of Nairobi, Nairobi, Kenya; University of Nairobi, Nairobi, Kenya; University of Nairobi, Nairobi, Kenya

## Introduction

There is a rising burden of non-communicable diseases (NCDs) in Africa as a result of a rise in risk factors (RFs) due to the epidemiological transition. These conditions are largely asymptomatic, especially early on. Contact with patients with inter-current illnesses offers an opportunity for opportunistic screening. However primary healthcare facilities are usually overcrowded, understaffed and underequipped, making screening difficult, hence a lost opportunity. We investigated the prevalence of selected RFs in ambulatory patients attending primary healthcare facilities in Nairobi.

## Methods

Twenty-four public and private facilities registered in Nairobi were randomly selected. Patients, 35 years and above on their index visit to the facility for the particular complaint, were eligible for recruitment. Every fifth eligible patient was recruited until the target of 10 per facility. Following informed consent, a history was taken for socio-demographic data, alcohol and tobacco use, self-reported hypertension, diabetes and HIV status, and drug history. Blood pressure, weight and height for BMI, and waist and hip circumference were measured. Fasting glucose, fasting lipid profile, serum creatinine for calculation of eGFR and serum uric acid levels were assayed. Continuous data are presented as means and categorical data as percentages. Prevalence of RFs is presented as proportions with 95% confidence intervals (CI). Correlations were done using the chi-square test.

## Results

We studied 213 patients. Mean age was 45 ± 9.4 years, and 64.3% were female. The prevalence (CI) of the RFs in the patients were as follows: smoking: 5.6% (2.9–96); obesity: 34.9% (28.5–41.7); diabetes: 8% (4.7–12.5), hypertension: 23.5% (18–29.5), and dyslipidaemia: 73.7% (67.3–79.5); 16.3% (11–22) had abnormal kidney function, 14.6% were in stage 2 and 1.4% in stage 3 renal failure. Urinary albumin was not done so it was not possible to identify stage 1 CKD. A total of 57% of hypertensives and 63% of diabetics were newly recognised. There was a statistically significant gender difference in smoking: male 14.5%, female 0.7% (*p* = 0.0001) and obesity: male 23.55%, female 41.2% (*p* = 0.01)

## Conclusions

There was a high prevalence of cardiometabolic RFs, most of it unrecognised, in attendees of primary healthcare facilities for routine care. This offers an opportunity for opportunistic screening. Training of primary healthcare workers and equipping the facilities to enable screening is an important step in the control of NCDs.

# Abstract: Risk-factor profile and co-morbidities in 2 398 patients with newly diagnosed hypertension from the Abuja heart study

**Authors:** Dike Ojji, Elena Libhaber, John Atherton, Jacob Alfa, Bolaji Abdullahi, Ada Nwankwo, Karen Sliwa

**Affiliations:** Cardiology Unit, Department of Medicine, University of Abuja Teaching Hospital, Gwagwalada, Abuja; Hatter Institute for Cardiovascular Research in Africa, Department of Medicine, Faculty of Health Sciences, University of Cape Town, South Africa; Cardiology Unit, Department of Medicine, University of Abuja Teaching Hospital, Gwagwalada, Abuja; Cardiology Unit, Department of Medicine, University of Abuja Teaching Hospital, Gwagwalada, Abuja; Cardiology Unit, Department of Medicine, University of Abuja Teaching Hospital, Gwagwalada, Abuja; Cardiology Unit, Department of Medicine, University of Abuja Teaching Hospital, Gwagwalada, Abuja; Hatter Institute for Cardiovascular Research in Africa, Department of Medicine, Faculty of Health Sciences, University of Cape Town, South Africa

## Introduction

Risk factors, co-morbidities and end-organ damage in newly diagnosed hypertension are poorly described in larger cohorts of urban African patients undergoing epidemiological transition.

## Methods

We prospectively collected detailed clinical, biochemical and echocardiography data of all subjects with hypertension as the primary diagnosis in patients presenting at the Cardiology Unit of the University of Abuja Teaching Hospital over an eight-year period.

## Results

There were 1 187 female patients (49.4%) of the 2 398 subjects, with a mean age of 51 ± 12.8 years. Presenting symptoms and signs were most commonly palpitation in 691 patients (28.8%), followed by dyspnoea on exertion in 541 (22.6%), orthopnea in 532 (22.2%), pedal oedema in 468 (19.5%), and paroxysmal nocturnal dyspnoea in 332 (13.8%), whereas only 31(1.3%) presented with chest pain. Risk factors were obesity in 671 patients (28%), 523 (21.8%) had a total cholesterol level > 5.2 mmol/l, diabetes mellitus was present in 201 (8.4%) and 187 (7.8%) were smokers.

End-organ damage was present in the form of echocardiographic left ventricular hypertrophy in 1 336 patients (55.7%), followed by heart failure in 542 (22.6%). Arrhythmias occurred in 110 (4.6%) of cases, cerebrovascular accident in 103 (4.3%), chronic kidney disease in 26 (1.1%), hypertensive encephalopathy in 10 (0.4%) and coronary artery disease in six (0.26%). There were marked differences in gender as women were more obese and men presented with more advanced disease.

## Conclusion

The burden of hypertension and its complications in this carefully characterised African cohort was quite enormous with more than two-fifth having one form of complication. The need of effective primary and secondary preventive measures to be mapped out to tackle this problem cannot be overemphasised.

# Abstract: Right ventricular systolic dysfunction is common in hypertensive heart failure: a prospective study in sub-Saharan Africa

**Authors:** Dike Ojji, Lionel Opie

**Affiliations:** Cardiology Unit, Department of Medicine, University of Abuja Teaching Hospital, Gwagwalada, Abuja, Nigeria; Hatter Institute for Heart Research, Interuniversity Cape Heart Group of the MRC, University of Cape Town Medical School, South Africa

## Introduction

Right ventricular (RV) systolic dysfunction is now widely recognised as a strong and independent predictor of adverse outcomes in patients with heart failure (HF). Reduction of RV systolic function more closely predicts impaired exercise tolerance and poor survival than does left ventricular (LV) systolic function. In spite of this, there is a dearth of data on RV function in hypertensive HF, which is the commonest form of HF in sub-Saharan Africa. We therefore conducted a prospective cohort study of hypertensive HF patients presenting to the University of Abuja Teaching Hospital, Abuja, Nigeria over an eight-year period.

## Methods

This was prospective cohort study. Hypertension was defined according to the JNC VII guidelines. The subjects also had one or more clinical features of long-standing hypertension, which included thickened arterial wall, locomotor brachialis and at least grade 2 hypertensive retinopathy. HF was diagnosed according to the guidelines of the European Society of Cardiology. The functional status of the HF subjects was categorised according to the New York Heart Association functional classification. RV systolic function was defined as a tricuspid annular plane systolic excursion (TAPSE) < 15 mm using M-mode echocardiography.

## Results

RV systolic dysfunction was identified in 272 (44.5%) of the 611 subjects that were studied. Subjects with TAPSE < 15 mm had a worse prognosis compared to those with TAPSE ≥ 15 mm.There was a significant correlation between TAPSE and other adverse prognostic markers, including left and right atrial area, LV size, LV mass, LV ejection fraction, restrictive mitral inflow and RV systolic pressure (RVSP). However, LV ejection fraction and right atrial area were the only independent determinants of RV systolic dysfunction.

## Conclusion

Hypertensive HF is a major cause of RV systolic dysfunction, even in a population with a low prevalence of coronary artery disease, and RV systolic dysfunction is associated with a poor prognosis in hypertensive HF. Detailed assessment of RV function should therefore be part of the echocardiographic evaluation of patients with hypertensive HF.

# Abstract: Assessing antihypertensive adherence with therapeutic drug monitoring

**Authors:** Brian Rayner, Erika Jones, Sandra Castel, Marc Blockman, Eric deCloedt, Sylva Schwager, Edward Sturrock, Maia Lesossky

**Affiliations:** University of Cape Town, Groote Schuur Hospital, Cape Town, South Africa; University of Cape Town, Groote Schuur Hospital, Cape Town, South Africa; University of Cape Town, Groote Schuur Hospital, Cape Town, South Africa; University of Cape Town, Groote Schuur Hospital, Cape Town, South Africa; University of Cape Town, Groote Schuur Hospital, Cape Town, South Africa; University of Cape Town, Groote Schuur Hospital, Cape Town, South Africa; University of Cape Town, Groote Schuur Hospital, Cape Town, South Africa; University of Cape Town, Groote Schuur Hospital, Cape Town, South Africa

## Objective

The proportion of South African hypertensive patients with controlled blood pressure (BP) is low. Non-adherence may play an important role but monitoring adherence remains difficult. Two commonly used antihypertensives are amlodipine and enalapril. This study aimed to determine whether monitoring amlodipine levels and inhibition of angiotensin converting enzyme (ACE) are feasible means to determine patient adherence.

## Methods

Patients attending a referral clinic for resistant hypertension who were prescribed enalapril and amlodipine (± other antihypertensives) were enrolled. After informed consent, patients underwent BP monitoring, filled in a questionnaire on adherence, and blood was sampled for amlodipine levels and ACE activity. Assessments were repeated. Amlodipine was assayed using liquid chromatography–mass spectrometry. The degree of ACE inhibition was determined by the z-phenylalanine-histidine-leucine and hippuryl-histidine-leucine (zFHL/HHL) ratio.

## Results

One hundred patients (age ± 50.5 years and 46% male) were enrolled, with 65 follow-up assessments. There was no difference between the mean BP from visit one to two. Most patients (90%) self-medicated, and 24% used pillboxes.

## ACE inhibitor results

control data suggest a zFHL/HHL ratio < 1.4 to be consistent with no ACE inhibition. Ten patients (17%) were found to be non-adherent at both visits and 12 (20%) at either visit one or two; 38 (63%) were adherent at both visits. Five patients had missing data. There were significant differences in BP between adherent and non-adherent patients: 141 ± 22/84 ± 15 mmHg vs 168 ± 30/105 ± 18 mmHg for visit one (*p* < 0.0001); and 146 ± 23/86 ± 17 mmHg vs 172 ± 34/99 ± 22 mmHg for visit two (*p* = 0.002 for SBP and *p* = 0.19 for DBP).

## Amlodipine results

an undetectable level of amlodipine was found at both visits in 10 (15.4%) patients and in four (6.2%) patients at either visit. Fifty-one (78.5%) patients were adherent at both visits. The BP of patients adherent to amlodipine at visit one was 141 ± 22/85 ± 14 mmHg vs 176 ± 27/108 ± 19 mmHg in the non-adherent group (*p* < 0.0001). At visit two the adherent group had a BP of 148 ± 29/86 ± 17 mmHg vs 173 ± 21/102 ± 22 mmHg (*p* = 0.008 and *p* = 0.005 for SBP and DBP). At both visits 67% of those non-adherent to amlodipine were non-adherent to enalapril.

## Conclusion

Monitoring antihypertensive adherence through therapeutic drug monitoring is a feasible option at the clinic. Non-adherence strongly predicts the presence of uncontrolled BP.

# Abstract: Echocardiographic assessment of pulmonary artery systolic pressure and outcomes in ambulatory heart failure patients: a Moroccan study

**Authors:** Fatima Ezzahra Sabri, Meriam Abelhad, Rachida Habbal

**Affiliations:** Department of Cardiology, Ibn Rochd University Hospital, Casablanca, Morocco; Department of Cardiology, Ibn Rochd University Hospital, Casablanca, Morocco; Department of Cardiology, Ibn Rochd University Hospital, Casablanca, Morocco

## Purpose

Pulmonary hypertension (PH) is a predictor of morbidity and mortality in patients with chronic heart failure (HF) but it is poorly described in our population. We sought to study the prevalence, determinants and prognostic significance of PH in a large representative population with HF.

## Methods

We retrospectively studied 1 613 patients with chronic heart failure on the HF registry of the University Hospital Ibn Rochd, Casablanca. Systolic pulmonary artery pressure (PASP) was determined by echocardiography and pulmonary hypertension was defined as PASP > 50 mmHg.

## Results

The proportion of patients with PASP ≥ 35, ≥ 40, ≥ 45 and ≥ 50 mmHg was: 26.47, 22.87, 17.24 and 12.15%, respectively. PH was present in 196/1 613 patients, with an average age of 69 years. There was 40.30% women and 59.70% men; 96.43% of patients had low left ventricular ejection fraction (LVEF) and only 1.02% had preserved LVEF. The aetiology of HF was predominantly ischaemic (17.86%). Most patients were receiving diuretics (66.33%), beta-blockers (58.16%) and ACE inhibitors (86.22%), whereas 55.10% were on spironolactone. The aetiology of PH was mostly due to left heart disease, while only 1.02% was due to primary HF and 2.04% of patients had chronic obstructive pulmonary disease (COPD). Patients with PH had a rate of 6.06% occurrence of acute heart failure decompensation (AHFD). The association with right ventricular dysfunction (RVD) increased the risk of AHFD and complications. We noted that patients with both PH and RVD had a greater risk of AHFD (14.28%).

## Conclusion

PH is common in HF patients, which is associated with a worse LV function and it provides incremental prognostic information. The combination of PH and RVD is particularly ominous: elevated PASP, determined by echocardiography, can identify ambulatory patients with HF at increased risk for adverse events.

# Abstract: Prevalence and factors associated with non-dipping pattern in Moroccan hypertensive patients: about 50 cases reports

**Authors:** Fatima Ezzahra Sabri, Ayoub Elhammiri, Tarik Bentaouen, Rachida Habbal

**Affiliations:** Department of Cardiology, Ibn Rochd University Hospital, Casablanca, Morocco; Department of Cardiology, Ibn Rochd University Hospital, Casablanca, Morocco; Department of Cardiology, Ibn Rochd University Hospital, Casablanca, Morocco; Department of Cardiology, Ibn Rochd University Hospital, Casablanca, Morocco

## Objective

Ambulatory blood pressure (BP) monitoring has become useful in the diagnosis and management of hypertensive individuals. In addition to 24-hour values, the circadian variation of BP adds prognostic significance in predicting cardiovascular outcome. Our aims were to determine the prevalence of non-dipping patterns and to assess clinical conditions associated with this status in groups of both treated and untreated hypertensive subjects, studied separately.

## Methods

Clinical data and 24-hour ambulatory BP monitoring were obtained from 50 hypertensive patients. There were 20 previously untreated and 30 treated hypertensive patients. Ambulatory BP monitoring was performed on 40 patients with an oscillometric device. A non-dipping pattern was defined when the nocturnal systolic BP dip was < 10% of the daytime systolic BP.

## Results

The prevalence of non-dipping was 44% in the untreated group and 52% in treated patients. In both groups the nondipping pattern was associated with advanced age, obesity, diabetes mellitus and overt cardiovascular or renal disease. In treated patients, non-dipping was associated with the use of a higher number of antihypertensive drugs but not with the time of the day at which antihypertensive drugs were administered.

## Conclusion

The non-dipping pattern is common in hypertensive patients. A clinical pattern of high cardiovascular risk is associated with non-dipping, so it is important to assess this measurement in hypertensive patients.

# Abstract: Value of non-invasive assessment of pulmonary hypertension in heart failure with preserved ejection fraction, and its prognostic importance

**Authors:** Fatima Ezzahra Sabri, Meriam Abelhad, Rachida Habbal

**Affiliations:** Department of Cardiology, Ibn Rochd University Hospital, Casablanca, Morocco; Department of Cardiology, Ibn Rochd University Hospital, Casablanca, Morocco; Department of Cardiology, Ibn Rochd University Hospital, Casablanca, Morocco

## Objectives

Pulmonary hypertension (PH) is a frequent complication in heart failure (HF). However, its impact factor in heart failure with preserved ejection fraction (HFPEF) is not well known. This study aimed to identify the prognostic value of PH measured by echocardiography in patients with HFPEF.

## Methods

A retrospective study of 384 patients with HFPEF (LVEF > 45%) from the heart failure registry of the Ibn Rochd University Hospital of Casablanca, Morocco. Pulmonary hypertension was defined as pulmonary artery systolic pressure (PASP) > 35 mmHg, measuring tricuspid regurgitation velocity and atrial pressure using Doppler echocardiography. The primary endpoint was all-cause readmissions for acute heart failure decompensation (AHFD) during a six-year follow up.

## Results

A total of 71 patients (18.5%) had PASP > 35 mmHg and 164 patients had PASP < 35 mmHg (47%). The average age was 64 ± 3 years, 40.30% were women and 59.70% were men. The primary endpoint was observed in 20 patients with PH (28%) who were readmitted for AHFD, whereas 112 patients without PH had a rate of 7% of developing AHFD. Patients with PH were an increased adjusted risk for the primary endpoint.

## Conclusions

Pulmonary hypertension (PASP > 35 mmHg), measured by non-invasive methods, is a strong and independent predictor of an unfavourable outcome in patients with heart failure and normal or only mildly reduced ejection fraction.

# Abstract: Prevalence and outcomes of pulmonary hypertension and diastolic dysfunction in a heart failure population

**Authors:** Fatima Ezzahra Sabri, Meriam Abelhad, Rachida Habbal

**Affiliations:** Department of Cardiology, Ibn Rochd University Hospital, Casablanca, Morocco; Department of Cardiology, Ibn Rochd University Hospital, Casablanca, Morocco; Department of Cardiology, Ibn Rochd University Hospital, Casablanca, Morocco

## Background

Pulmonary hypertension due to left heart disease is very common. Our aim was to investigate the prevalence and relationship between the severity of left ventricular diastolic dysfunction and pulmonary hypertension (PH) in a heart failure (HF) population.

## Methods

We retrospectively studied 1 613 patients with chronic heart failure in the HF registry of the University Hospital of Ibn Rochd, Casablanca. Transthoracic echocardiography was used to categorise diastolic function and estimate pulmonary artery pressure. Systolic pulmonary artery pressure (PASP) was determined by echocardiography and pulmonary hypertension was defined as PASP > 35 mmHg. Grade 1 diastolic dysfunction was determined if the E/A ratio was < 0.8. An E/A ratio of 0.8–1.5 was classified as grade 2 diastolic dysfunction, and E/A > 1.5 as grade 3. If no data on E/A ratio were available, the ratio of early transmitral flow velocity (E) to mitral annular diastolic velocity (e’) was used. Grade 1 was assumed if E/e’ was < 8, representing impaired relaxation with normal filling pressures. E/e’ of 8–15 was classified as grade 2, and E/e’ > 15 as type 3.The endpoint was the readmission for AHFD (acute heart failure decompensation).

## Results

Among 1 613 HF patients, PH was present in 21% of patients with grade 1 diastolic dysfunction, in 20% of patients with grade 2, and in 41% of patients with grade 3. Outcomes were not related to the severity of diastolic dysfunction but were worse in patients with PH. Patients with PH had a rate of 21% occurrence of AHFD, while only 13% of patients without PH had AHFD.

## Conclusions

Our data indicate that HF patients with even mild diastolic dysfunction often have PH. Given the discrepancy in influence of diastolic dysfunction and PH on outcome, PH may play an independent and previously underestimated pathophysiological role in HF.

# Abstract: Devising useful algorithms based on electrocardiographic findings to discriminate between right coronary artery and left circumflex artery occlusion in acute inferior wall myocardial infarction patients

**Authors:** Ramdhany Sachin, Guo Ning

**Affiliations:** Flacq Hospital, Telfair Moka, Mauritius; First Affiliated Hospital of Xian Jiaotong University, China

## Background

Although it is generally considered that patients with acute ST-segment elevation myocardial infarction (STEMI) in the inferior wall have a more favourable prognosis than those with STEMI in the anterior wall, potential complications exist when the culprit artery is the right coronary artery (RCA). We therefore compared old and new electrocardiographic (ECG) criteria, which could be useful for early identification of the culprit vessel between the RCA and left circumflex (LCX) artery in patients with inferior wall STEMI.

## Methods

A total of 194 patients with acute STEMI in the inferior wall admitted consecutively to our hospital were enrolled into this study. Eighteen-lead ECGs recorded on admission, at a speed of 25 mm/s and voltage of 10 mm/mV, were analysed. ST-segment deviation was measured with a hand-held calliper and magnifying glass at the J-point in all available leads. Coronary angiography was performed within the first 12 hours from symptom onset. Of these, 166 patients were identified with the culprit lesion in the RCA and 28 patients with the lesion in the LCX.

## Results

Altogether the sensitivity and specificity of 10 old and six new ECG criteria were examined. Two of the new criteria proved their utility in identifying the RCA and the LCX as the infarct-related artery: (1) the ratio of std I/aVL ≤ 1, which yielded a sensitivity of 86.7%, a specificity of 92.8% and a positive predictive value of 98.6%, made it an accurate marker for an RCA occlusion; (2) STE or isoelectric ST in lead I, which yielded a sensitivity of 93%, a specificity of 88% and a positive predictive value of 56.5%, made it a highly accurate criterion to diagnose LCX as the infarct-related artery in patients with acute inferior wall STEMI.

## Conclusions

Two new ECG criteria in combination with previous criteria can be used to discriminate the RCA from the LCX as the culprit artery at the bedside in patients with acute inferior wall STEMI. The ECG is useful in differentiating between RCA and LCX involvement in acute inferior wall STEMI before primary percutaneous coronary intervention, allowing decisions about therapy to be taken at the earliest possible time.

# Abstract: Echocardiographic predictors of outcome in acute heart failure patients in sub-Saharan Africa: insights from THESUS-HF

**Authors:** Mahmoud U Sani, Beth A Davison, Gad Cotter, Albertino Damasceno, Bongani M Mayosi, Okechukwu S Ogah, Charles Mondo, Anastase Dzudie, Dike B Ojji, Kouam Charles Kouam, Ahmed Suliman, Gerald Yonga, Ba Sergine Abdou, Fikru Maru, Bekele Alemayehu, Christopher Edwards, Karen Sliwa

**Affiliations:** Department of Medicine, Bayero University Kano; Aminu Kano Teaching Hospital, Kano, Nigeria; Momentum Research Inc, Durham, North Carolina, USA; Momentum Research Inc, Durham, North Carolina, USA; Faculty of Medicine, Eduardo Mondlane University, Maputo, Mozambique; Department of Medicine, GF Jooste and Groote Schuur Hospitals, University of Cape Town, Cape Town, South Africa; Department of Medicine, University College Hospital Ibadan and Ministry of Health, Abia State, Nigeria; Uganda Heart Institute, Kampala, Uganda; Department of Internal Medicine, Douala General Hospital and Buea Faculty of Health Sciences, Douala, Cameroon; Department of Medicine, University of Abuja Teaching Hospital, Abuja, Nigeria; Department of Internal Medicine, Douala General Hospital and Buea Faculty of Health Sciences, Douala, Cameroon; Faculty of Medicine, University of Khartoum, Khartoum, Sudan; Department of Medicine, Aga Khan University, Nairobi, Kenya; Service de Cardiologie, Faculte de medecine de Dakar, Dakar, Senegal; Addis Cardiac Hospital, Addis Ababa, Ethiopia; Addis Cardiac Hospital, Addis Ababa, Ethiopia; Momentum Research Inc, Durham, North Carolina, USA; Hatter Institute for Cardiovascular Research in Africa, Department of Medicine, Faculty of Health Sciences, University of Cape Town, South Africa

## Background

The role of echocardiography in the risk stratification of acute heart failure (HF) is unknown. Some small studies and retrospective analyses have found little change in echocardiographic variables during acute HF admission and some echocardiographic parameters were not found to be associated with outcomes. It is unknown which echocardiographic variables will predict outcomes in sub-Saharan African patients admitted with acute HF. Using echocardiograms, this study aimed to determine the predictors of death and readmissions within 60 days and deaths within 180 days in patients with acute HF.

## Methods and Results

Out of the 1 006 patients in the THESUSHF registry, 954 had an echocardiogram performed within a few weeks of admission. Echocardiographic measurements were performed according to the American Society of Echocardiography guidelines. We examined the associations between each echocardiographic predictor and outcome using regression models. Heart rate and left atrial size predicted death within 60 days or readmission. Heart rate, left ventricular posterior wall thickness (PWTd) and presence of aortic stenosis were associated with the risk of death within 180 days. PTWd added to the clinical variables in predicting 180-day mortality.

## Conclusions

Echocardiographic variables, especially those of left ventricular size and function, were not found to have additional predictive value in patients admitted for acute HF. Left atrial size, aortic stenosis, heart rate and measures of hypertrophy (LV PWTd) had some predictive value, suggesting the importance of early treatment of hypertension and severe valvular heart disease.

# Abstract: Role of Amp-Activated Protein Kinase (AMPK) in vascular endothelial protection

**Authors:** Aamir Shamsi, Justin Martin

**Affiliations:** Imperial College, London, UK; Imperial College, London, UK

## Background

Adenosine monophosphate-activated protein kinase (AMPK), although known for its role in regulating cellular metabolism, has recently emerged as an important kinase involved in vascular endothelial protection. We noted it to have similar actions to those of MnSOD, HO-1 and DAF, genes that protect the endothelium from inflammatory and oxidative stress. Therefore we investigated whether AMPK’s cytoprotective activity includes the induction of these genes, and explored the signalling pathways that may be involved.

## Methods

Human umbilical vein endothelial cells first underwent a flow perfusion assay. They were then treated with AICAR (an AMPK activator) for 24 hours, AMPK adenovirus (Ad CA-AMPK) for 18 hours or a combination of atorvastatin and rapamycin for two hours, and either immunoblotted for various proteins or analysed via flow cytometry. Transcription factor CREB was silenced using siRNA.

## Results

In this study we showed that oscillatory shear stress may be responsible for down-regulating levels of phospho-AMPK and *HO-1*. Cells treated with AICAR had a significant increase in *MnSOD*, *HO-1* and *DAF* protein expression. Ad CA-AMPK was shown to deliver active forms of AMPK into the cells and infection of this adenovirus also up-regulated the levels of *MnSOD*, *HO-1* and *DAF*. We also showed that AMPK activates CREB. Our results suggest that depletion of CREB with siRNA reduces *MnSOD* protein induction by Ad CA-AMPK.

## Conclusion

We showed that AMPK activation induces the cytoprotective genes *MnSOD, HO-1*, and for the first time, *DAF*. We have also suggested that CREB may be involved in the pathway for AMPK-dependent induction of *MnSOD* and that AMPK activation may be a future therapy target.

# Abstract: Protocol: what factors influence health-seeking behavior and adherence to secondary prophylaxis for patients with sore throat, acute rheumatic fever and rheumatic heart disease?

**Authors:** Thembekile Shato, Tayla Hermanus, Mark Engel, Jantina De Vries, Liesl Zühlke

**Affiliations:** Department of Paediatrics, Faculty of Health Sciences, University of Cape Town, South Africa; Department of Medicine, Faculty of Health Sciences, University of Cape Town, South Africa; Department of Medicine, Faculty of Health Sciences, University of Cape Town, South Africa; Department of Medicine, Faculty of Health Sciences, University of Cape Town, South Africa; Department of Paediatrics, Faculty of Health Sciences, University of Cape Town, South Africa; Department of Medicine, Faculty of Health Sciences, University of Cape Town, South Africa

## Introduction

The high prevalence of rheumatic heart disease (RHD) in low-income compared to high-income settings is reflective of the impact of socio-economic factors on disease aetiology and prognosis. The intersection of these biosocioeconomic factors influences health-seeking behaviour for those with throat infections, and adherence to secondary prophylaxis for acute rheumatic fever (ARF) patients. The dearth of information around the experiences of RHD patients in endemic communities curtails the formulation and implementation of effective programmes that seek to prevent the occurrence and control of RHD. Our aim is to identify factors that influence health-seeking behaviour at different stages of disease and adherence to secondary prophylaxis utilising a rapid ethnographic approach.

## Methods and Results

We propose to use a mixed-methods qualitative research design to explore patients’ experiences from primary infection to overt RHD. The population will be composed of three patient groups: patients with throat infection, those with ARF and those with RHD. We will employ non-probability sampling techniques to select respondents. We anticipate using patient interviews, focus groups and novel techniques, such as photo elicitation and digital storytelling. Findings will be triangulated to explore relationships, note contractions and identify common barriers.

## Conclusion

This protocol will determine the factors that influence health-seeking behaviour and adherence to secondary prophylaxis for patients with sore throat, ARF and RHD across diverse communities. In addition, it should identify opportunities for interventions and note challenges associated with the formulation and implementation of such interventions. A final possible benefit would be the application to the evaluation of already existing or newly formulated RHD control programmes.

# Abstract: Role of echocardiography and cardiac CT in the diagnosis of constrictive pericarditis

**Authors:** Saad Subahi

**Affiliations:** Khartoum, Sudan

Constrictive pericarditis is a commonly missed diagnosis. The reasons for that are casual clinical examination, casual echocardiography studies, acute presentation, predominant gastric symptoms and absence of calcification in the chest X-ray in some patients. In most patients, comprehensive echocardiography may provide conclusive evidence of constrictive pericarditis. Therefore echocardiography remains the initial imaging method of choice for the majority of the patients with pericardial constriction. Additional testing is needed in some clinical scenarios to make the diagnosis with more confidence .

In this presentation the role of echocardiography and cardiac CT in the diagnosis of constrictive pericarditis is discussed. An important reason to use echocardiography early in the diagnostic process is to rule out other common causes of CCF such as cardiomyopathy and unrecognised valvular disease. Encasement of the heart by stiff pericardium leads to abrupt cessation of ventricular diastolic filling, it also isolates the heart from respiratory changes in intrathoracic pressure and causes exaggerated ventricular interdependence. These pathophysiological findings are well demonstrated by 2D, M-mode and Doppler findings. Early diastolic septal noticing, flattening of the left ventricle posterior wall in diastole and dilatation of the inferior vena cava are classic M-mode and 2D features associated with constrictive pericarditis. Doppler interrogation of transmitral flow velocity shows early rapid restrictive filling with reciprocal respiratory changes between transmitral and tricuspid flow or between pulmonary and hepatic flow. Cardiac computed tomography (CT) on the other hand provides excellent anatomic delineation of the pericardium and enables a precise measurements of pericardial thickness, it also shows abnormalities of the contour of pericardium and conical deformity of the ventricles.

# Abstract: Blood pressure pattern of preschool children in Port Harcourt city, Nigeria

**Authors:** Petronila Tabansi, Kelechi Onyemkpa, Barbara Otaigbe, Ifeoma Anochie

**Affiliations:** University of Port Harcourt, Choba, Port Harcourt, Nigeria;; Maitama District Hospital, Abuja; University of Port Harcourt, Choba, Port Harcourt, Nigeria;; University of Port Harcourt, Choba, Port Harcourt, Nigeria;

## Introduction

Blood pressure (BP) is the force exerted by circulating blood on the walls of the blood vessels. It is an important vital sign in children and adults, and abnormal levels in childhood can track into adulthood, especially when persistent. Sustained high BP levels can result in end-organ damage in affected individuals, with consequent high morbidity that is potentially fatal. Precursors of high blood pressure are sometimes present in young children long before manifestation in adulthood. This suggests the possibility for early identification of children who are at risk of developing hypertension as adults. Nigeria has a high prevalence of essential hypertension among its adult population, yet scarce information exists on the BP pattern of their preschool sub-population. There is no cure for essential hypertension, therefore primary prevention by screening and intervention in childhood is essential to avert tracking into adulthood.

## Aim

To determine the blood pressures pattern in preschool children in Port Harcourt City (PHC) and therefore identify those at potential risk for tracking into adulthood.

## Methods

A multistage sampling technique was used to randomly select preschool children (2–5 years) from the three school districts in PHC. Ethical clearance, permission and consent for the study were obtained from the respective authorities. Pupils’ anthropometry (weight and height) was done and body mass index (BMI) was calculated. Basal BP was then measured under resting conditions.

## Results

There were 710 pupils, 365 (51.4%) were male and 345 (48.6%) female. Age ranged from two to five years. Mean systolic BP was 93.2 ± 10.6 mmHg while mean diastolic BP was 58.8 ± 8.0 mmHg. Mean systolic BP of the males was significantly higher, at 94.0 ± 9.7 mmHg compared to 92.4 ± 11.5 mmHg for the females (p = 0.045). Mean diastolic BP of males was also higher than that of females. The majority (633, 89.1%) had normal BP while 77 children (10.9%) were hypertensive. Hypertension was commoner (15.9%) among the three-yearold children. Males had a higher prevalence of hypertension (11.8%) compared to females (9.9%).

## Conclusion

The blood pressure pattern in preschool children in PHC demonstrates a high prevalence of hypertension at 10.9%. There is a need for routine periodic screening of this population sub-group for early intervention to prevent tracking into adulthood.

# Abstract: BNP predicts systolic dysfunction in pregnant women with cardiac disease

**Authors:** Kemi Tibazarwa, Lauren Nicholson, Tasneem Adam, Catherin Elliott, John Anthony, Elena Libhaber, Karen Sliwa

**Affiliations:** The Hatter Institute for Cardiovascular Research in Africa, Cape Town, South Africa, and Muhimbili National Hospital, Dar es Salaam, Tanzania; University of Cape Town, South Africa; The Hatter Institute for Cardiovascular Research in Africa, Cape Town, South Africa; Department of Obstetrics and Gynaecology, University of Cape Town, South Africa; Department of Obstetrics and Gynaecology, University of Cape Town, South Africa; University of the Witwatersrand, Johannesburg, South Africa; The Hatter Institute for Cardiovascular Research in Africa, Cape Town, South Africa

## Introduction

In pregnancy, the importance of BNP levels have mostly been evaluated in pre-eclampsia. Changes in BNP levels among women with cardiovascular disease (CVD) complicating pregnancy have not been characterised.

## Methods

As part of an on-going registry at a specialised tertiary clinic in Cape Town, we examined the serum BNP levels in 152 consecutive patients manifesting cardiovascular disease in pregnancy, or within six months postpartum. Each patient underwent clinical assessment, ECG, echocardiography and laboratory tests, including serum BNP levels, which were performed at baseline and six-months follow up. Twenty controls with normal pregnancy also had BNP measurement; half being antepartum, and half postpartum.

## Results

Median BNP levels were 95 pg/ml (IQR 47–186) at baseline, and 91 pg/ml (IQR 47–181) at follow up. The baseline and follow-up BNP levels in patients with cardiac disease who either presented postpartum or within the first trimester of pregnancy were 2.5 times greater than those presenting during the second and third trimester (*p* = 0.0003 and 0.0007, respectively). This differed from comparisons of BNP levels of 10 healthy pregnant women to 10 healthy women postpartum, where the BNP levels postpartum were not statistically different from those antepartum (*p* = 0.17).

There was no demonstrable association between gestational age, or premature delivery and the corresponding BNP levels. Despite a 16% sero-prevalence of HIV in our cohort, there was no association with BNP levels in this cohort.

In addition to elevated BNP levels in patients with left heart failure, patients with isolated right heart failure had BNP levels twice that of those without right heart failure (*p* = 0.003), although this was not associated with any particular underlying primary diagnosis. Nine patients died within the six-month follow-up period (6%), with the majority dying in the postpartum period. Serum BNP levels at baseline and six months each predicted left ventricular (LV) dimensions and systolic function at each visit, respectively, as well as death. Furthermore, baseline serum BNP was able to predict LV size and dimensions at six months.

## Conclusions

Serum BNP levels correlated well with LV dimensions, as well as LV and RV function, and may prove useful as a simple tool for risk stratification of pregnant and postpartum patients with cardiac disease who are at risk of poorer outcomes. Larger studies may help to distinguish more specific associations.

This study highlights some of the challenges faced in diagnosing heart disease in pregnancy; where not only can the symptoms and some of the clinical signs overlap with those of normal pregnancy, but where BNP levels, commonly used in the management of heart failure, have been known to fluctuate throughout pregnancy, thereby rendering it difficult to use as a screening test for heart failure in pregnant women. Our study helps to address that challenge by showing that for women with heart disease presenting during certain periods of gestation and those postpartum, blood BNP measurement may be useful in detecting CVD, and perhaps even for prognosis.

# Abstract: The Tanzanian Cardiovascular Disease (TANCarD) registry: from pilot to full phase

**Authors:** Kemi Tibazarwa, Robert Mvungi, Peter Kisenge, Naiz Majani, Delilah Kimambo, Tatizo Waane, Tulizo Shemu, Sulenda Kubhoja, Bashir Nyangassa, Mohammed Janabi

**Affiliations:** Muhimbili National Hospital, Dar es Salaam, Tanzania; Muhimbili National Hospital, Dar es Salaam, Tanzania; Muhimbili National Hospital, Dar es Salaam, Tanzania; Muhimbili National Hospital, Dar es Salaam, Tanzania; Muhimbili National Hospital, Dar es Salaam, Tanzania; Muhimbili National Hospital, Dar es Salaam, Tanzania; Muhimbili National Hospital, Dar es Salaam, Tanzania; Muhimbili National Hospital, Dar es Salaam, Tanzania; Muhimbili National Hospital, Dar es Salaam, Tanzania; Muhimbili National Hospital, Dar es Salaam, Tanzania

## Introduction

Cardiovascular disease (CVD) has often taken the lead among the growing NCD epidemic that many African countries face, including that faced by Tanzania. However, still far too little is known about the true burden of CVD in Tanzania, including in Dar es Salaam. In recent years, scattered attempts have been made to describe the frequency of CVD and the risk factors in mostly rural parts of Tanzania. To date, no systematic study of the epidemiology and natural history of all-cause CVD has been done in Tanzania. Subsequently, there is no reliable data on the burden of CVD in Tanzania, including in the economic capital Dar es Salaam.

Within the national referral hospital Muhimbili National Hospital (MNH), the Department of Cardiovascular Medicine attends to over 17 000 patient visits a year (as out-patients and in-patients), with suspected or, in the vast majority, confirmed CVD). Given the magnitude of patient flow through this department, and its reflection of the profile of CVD at large, it is of highest importance to record and study our patient profile in great detail to better understand trends in CVD within our own setting.

## Methods

In order to bridge the knowledge gap on the burden of CVD in Tanzania, we initiated a registry of CVD as seen within the public sector in Dar es Salaam, Tanzania. The Department of Cardiovascular Medicine at MNH acts as the sole point within the public sector for definitive diagnosis of patients with CVD other than uncomplicated essential hypertension. Hence, by capturing all patients suspected of CVD in the public sector at the department of Cardiovascular Medicine at Muhimbili National Hospital and, through the referral system, its catchment area, we are essentially adopting a prospective populationbased study of symptomatic CVD within Dar es Salaam.

## Results

While this registry has only recently been launched, we describe the exciting data emerging from the first six months of study. At a glance, our study demonstrates a diverse array of CVD, from the more textbook untreated congenital and rheumatic heart diseases, to the surge of atherosclerotic cardiac and vascular disease. Of particular interest as well, however, is the astonishing number of connective tissue diseases uncovered through detailed work-up of the presenting CVD patients. Further to this, we include provisional data of short-term outcome for all patients, including those who received surgical or catheter-based interventions.

## Conclusions

Already this registry enables us to better depict the burden of CVD in Tanzania, and tempts a re-look into the popular assumptions of CVD in Tanzania. Furthermore, it provides a wake-up call into the principles around pharmaceutical prioritisation for Tanzania with regard to CVD, both in medications as well as in facilities for invasive therapy.

# Abstract: Health services for cardiometabolic diseases among people living with HIV/AIDS in low-and middle-income countries: a preliminary systematic review

**Authors:** Nathaniel Tulloch, David Watkins

**Affiliations:** Department of Internal Medicine, University of Washington, Seattle, USA;; Department of Medicine, University of Cape Town, Cape Town, South Africa; Department of Medicine, University of Washington, Seattle, WA, USA

## Background and objectives

The roll-out of antiretroviral drug therapy (ART) has led to a rapid increase in life expectancy among people living with HIV/AIDS (PLWHA) in low- and middle-income countries (LMIC), especially in sub-Saharan Africa. As a result, PLWHA are increasingly at risk for cardiometabolic diseases (CMD); yet studies have documented that, in many LMIC, CMD health services are severely constrained. At the same time, HIV/AIDS primary care services are relatively robust in many settings, creating unique opportunities and challenges around management of CMD among PLWHA. This study seeks to systematically review the evidence around health services for CMD among PLWHA in LMIC.

## Methods

We developed a systematic review protocol based on the PRISMA checklist. To characterise and synthesise the diverse literature in this area, we adapted a conceptual model for hypertension services (Khatib et al., 2014) to our research question. We sought to review either quantitative and qualitative studies on (1) the ‘cascade’ of care for CMD among PLWHA (i.e. awareness, treatment and control), and (2) barriers and facilitators to CMD care among PLWHA. The CMD included in this study were cardiovascular diseases, hypertension, type 2 diabetes, dyslipidaemia and chronic kidney disease. Our protocol outlines the databases to be searched, the search terms, and the article-screening process. Our results will be restricted to research based in LMIC and published in English since the year 1996.

## Results

A preliminary application of our search strategy to Medline yielded 1 313 titles. As a first step to refining our protocol, we screened publications from the past two years (150 titles), noting the following general trends. First, only 43 titles were retained for abstract screening. Many studies only reported the prevalence of metabolic dysfunction from ART (e.g. lipodystrophy). Only six abstracts had potential relevance to our research question around elements of the ‘cascade’ of CMD care. At the same time, these issues were the primary aim in only three of these studies. These studies found that PLWHA have poor understanding and low rates of diagnosis of CMD. Medication availability and low provider knowledge were also noted as ‘supply side’ barriers. At the same time, medication adherence clubs, and pedometer-based walking programmes were notable facilitators of CMD care. Only one study found that a majority (83%) of PLWHA with hypertension were on medication.

## Conclusions

This preliminary analysis suggests many knowledge gaps around CMD health services among PLWHA. There do appear to be important differences, compared to the general population in LMIC. Our next steps will be to refine the search protocol and terms, and comprehensively screen the literature. These results can then be used to develop tailored interventions to improve CMD care among PLWHA.

# Abstract: Anthropometric indices: optimal cut-off values for abdominal obesity in adult Nigerians

**Authors:** Ejiroghene Umuerri, Godson Eze

**Affiliations:** Delta State University Teaching Hospital, Oghara, Nigeria; Delta State University Teaching Hospital, Oghara, Nigeria

## Introduction

Obesity refers to the amount of excessive fat in the body. The distribution of fat is important as central obesity has been more linked to adverse cardiovascular risks than has general obesity. The cut-off values for body mass index (BMI), waist circumference (WC), waist–hip ratio (WHR) and waist-to-height ratio (WHtR) have been shown to vary with ethnicity. This study sets out to determine the optimal cut-off values for obesity in adult Nigerians using four anthropometric indices.

## Methods

This was a cross-sectional population study in Delta State, Nigeria. Data on weight, height, waist circumference and hip circumference were obtained using recommended protocols and BMI, WC, WHR and WHtR were calculated. Assuming BMI as the standard method for diagnosing obesity, the receiver operating characteristic (ROC) analysis was used to maximise the sensitivity and specificity of the other anthropometric indices.

## Results

A total of 866 participants aged 18 years and above were studied, 381 (44.0%) were males and 485 (56.0%) were females. The prevalence of obesity was 11.2% using a BMI of ≥ 30 kg/m^2^. Using cut-off values of WHtR ≥ 0.5 irrespective of gender, WC > 102 cm for men and > 88 cm for women and WHR > 0.95 for men and > 0.8 for women, the prevalence of obesity was 75.4, 13.9 and 54.3%, respectively. WHtR had the largest area under curve (*R* = 0.862, 95% CI: 0.803–0.923, *p* < 0.001) while it was 0.824 (95% CI, 0.764–0.885, *p* < 0.001) for WC and 0.622 (95% CI, 0.575–0.706, *p* < 0.001) for WHR.

The prevalence of obesity using the ROC-derived values for WHtR, WC and WHR was 23.09, 26.79 and 44.23%, respectively. The ROC indicates that in the setting of the study, the WHtR cut-off value should be 0.6, while WC should be 1.026 times and WHR 0.919 times the aforementioned cut-off values. Optimal cut-off values being proposed among adult Nigerians are WHtR ≥ 0.6, WC > 104 cm and > 90 cm for males and females and WHR > 0.87 and > 0.73 for males and females, respectively

## Conclusion

WHtR is the best screening tool for diagnosis of abdominal obesity in our setting. Optimal cut-off values for WHtR and WC are higher, while WHR is lower compared to existing values for the European population.

# Abstract: Obesity indices and peripheral artery disease measured by ankle–brachial index in Nigerian out-patients

**Authors:** Ejiroghene Umuerri, Austine Obasohan

**Affiliations:** Delta State University Teaching Hospital, Oghara, Nigeria; University of Benin Teaching Hospital, Benin, Nigeria

## Introduction

Peripheral artery disease (PAD), an important component of the cardiovascular triad, has been linked with obesity as one of the risk factors for its development. The risk posed by obesity however varies, depending on the indices measured.

## Methods

We compared the relationship of measurements of central and visceral obesity [waist circumference (WC) and waist–hip ratio (WHR)] versus that of general obesity [body mass index (BMI)] in the development of PAD among Nigerians with hypertension and/or diabetes mellitus. PAD was diagnosed when the ankle–brachial index (ABI) was < 0.9 in either limb.

## Results:

A total of 541 patients (194 males and 347 females) with a mean age of 58.4 (± 0.46) years were studied. The mean BMI, WC and WHR were 27.8 (± 0.222) kg/m2, 96.8 (± 0.515) cm and 0.941 (± 0.003), respectively. Although the mean BMI, WC and WHR were higher in patients with PAD than those without PAD, the difference was only statistically significant for WC and WHR (*p* = 0.003 and *p* = 0.016) but not BMI (*p* = 0.151). However, the difference in mean BMI was statistically significant in patients < 60 years (*p* = 0.015) but not in those ≥ 60 years (*p* = 0.953).

## Conclusion

This study has shown that in Nigerian Africans, measurement of central and visceral obesity were more related to the development of PAD than was BMI, which is a measure of general obesity. This lack of significance was probably due to the fact that PAD occurred more in older people as there was a significant relationship with PAD in people younger than 60 years old.

# Abstract: Prevalence and socio-demographic correlates of obesity and overweight in delta state, Nigeria: a rural–urban comparison

**Authors:** Ejiroghene Umuerri, Omotola Ayandele

**Affiliations:** Delta State University Teaching Hospital, Oghara, Nigeria; Delta State University Teaching Hospital, Oghara, Nigeria

## Introduction

Obesity is a lifestyle disease associated with multiple adverse health conditions such as type 2 diabetes mellitus, cardiovascular disease and some cancers. Its prevalence is on the increase globally, partly because of urbanisation. This study explored the differences in prevalence of overweight and obesity and their association with socio-demographic characteristics in rural and urban populations in Delta State, Nigeria

## Methods

A cross-sectional survey was carried out of households in Jesse (rural) and Warri (urban) using the modified WHO Steps.

## Results

There was a total of 866 respondents, 44.0 and 56.0% from rural and urban populations, respectively. The male versus female distribution was 49.9 vs 50.1% (rural) and 39.4 vs 60.6% (urban) (χ2 = 9.525, *p* = 0.002). The mean age (± SD) was 47.1 (± 19.0) years (rural) and 38.9 (± 12.2) years (urban) (*t* = 7.332, 95% CI: 6.004–10.396; *p* < 0.001). The difference in educational status between rural and urban populations was significant (χ2 = 308.123; df = 4; *p* < 0.001). The mean BMI was 23.05 (± 6.9) kg/m^2^ (rural) and 24.98 (± 5.6) kg/m^2^ (urban) (*t* = 1.936, 95% CI: 1.080–2.792; *p* = 0.015).

The overall prevalence of obesity and overweight was 11.2 and 20.8%, respectively, with urban being higher than rural (15.9 and 23.7% vs 5.2 and 17.1%, respectively). The prevalence of overweight was higher in females than males in both urban and rural settings (urban: 26.2 vs 19.9%; rural: 17.3 vs 16.8%). Differences in BMI categories between urban and rural settings was found only among females (females: χ^2^ = 29.800, df = 3, *p* < 0.001; males: χ^2^ = 6.191, df = 3, *p* = 0.103). The prevalence of overweight and obesity was highest among middle-aged (40–64 years) respondents compared with the young and elderly in both rural (19.7 vs 6.6%) and urban (31.1 vs 20.9%) and the difference in high BMI (≥ 25 kg/m^2^) between urban and rural setting in this age group was statistically significant (χ^2^ = 26.889, df = 3, *p* < 0.001).

The prevalence of overweight and obesity was higher among rural participants with secondary education than those with primary or no formal education. The reverse was the case for urban participants. The urban–rural differences in the association between educational status and prevalence of obesity and overweight (≤ primary: χ^2^ = 24.861, df = 3, *p* < 0.001; secondary: χ^2^ = 8.501, df = 3, *p* < 0.037).

## Conclusion

This study has shown that obesity and overweight was common, being higher in the urban than the rural population. High BMI was more prevalent among females and middleaged persons in both settings as well as in urban dwellers with higher levels of education.

# Abstract: Limited supplies of antihypertensive medications in primary care settings in three African countries

**Authors:** David Watkins

**Affiliations:** University of Cape Town, Groote Schuur Hospital, Cape Town, South Africa

## Background and objectives

Treatment guidelines and targets for reducing cardiovascular mortality in Africa assume that antihypertensive medications are routinely available in primary care settings. I sought to explore the availability of common antihypertensive drugs across three countries.

## Methods

I obtained data from nationally representative health facility surveys used in the Access, Bottlenecks, Costs, and Equity (ABCE) study. I included all facilities that provide hypertension care in Ghana, Kenya and Uganda. The antihypertensive drugs assessed in these surveys were captopril, lisinopril, atenolol, propranolol, nifedipine and hydralazine; both stocks and stock-out rates were recorded. I first conducted descriptive analyses of drug availability by country during 2011 (the last survey year); this included the presence of (1) individual drugs, (2) drug classes, and (3) at least one or two drugs (from different classes). I then used the longitudinal datasets from Kenya and Uganda (2007–2011) to estimate associations between facility characteristics and drug availability, employing logistic regression models with facility-level random intercepts.

## Results

While individual drug availability varied across countries, stock-out rates were generally < 10% across countries. However only 27% of Kenyan facilities reported carrying any ACE inhibitor, and only 37% of Ghanaian facilities carried any beta-blocker. In Ghana, 100% of facilities carried at least one drug, but only 51% of facilities had two or more drug options. Estimates for at least one versus at least two drugs were 67 vs 53% for Kenyan facilities and 82 vs 58% for Ugandan facilities, respectively. In both Kenya and Uganda, public facilities were significantly less likely and higher-level facilities significantly more likely to carry more than one antihypertensive drug; however the marginal effects were much larger in Uganda. In neither country was there an independent association across facility locale (rural vs semi-urban vs urban).

## Conclusions

Primary healthcare providers in three African countries appear to lack drug options for managing hypertension. About half of the surveyed facilities carried only one drug, and public and first-level clinics in Kenya and Uganda were more likely to have no antihypertensive drugs at all. Hence, important supply-side barriers remain to implementing hypertension ‘roadmaps’ and guidelines in Africa.

# Abstract: Strategies to reduce the burden of rheumatic fever and rheumatic heart disease in Africa: a cost-effectiveness analysis tool for local decision-making

**Authors:** David Watkins, Solomon J Lubinga, Joseph B Babigumira

**Affiliations:** University of Cape Town, Cape Town, South Africa; University of Washington, Seattle, USA; University of Washington, Seattle, USA; University of Washington, Seattle, USA

## Background and objectives

Mortality rates from acute rheumatic fever (ARF) and rheumatic heart disease (RHD) remain high in many parts of Africa. However, it is not clear whether to focus on preventing RHD or treating existing cases with surgery. We developed a model to assess the cost-effectiveness of scaling up primary prevention (PP), secondary prevention (SP), and valve surgery (VS), given local resource constraints.

## Methods

We created a Markov model of the natural history of ARF and RHD, taking transition probabilities, healthcare and programme costs, current intervention coverage levels, and intervention effectiveness data from previously published studies and expert opinion. We took a healthcare system perspective on costs, and measured outcomes as disability-adjusted life-years (DALYs), discounting both at 3%. We calculated incremental cost-effectiveness ratios (ICERs) for increasing coverage of PP, SP and VS to 70, 92 and 95%, respectively compared to the status quo level of coverage. We assessed three scenarios: (1) upper-middle-income country with existing surgical platform, (2) lower-middle-income country with no existing surgical platform, and (3) low-income country with no existing surgical platform. We used per capita gross domestic product (GDPpc) levels to assess cost effectiveness. We also estimated the impact of PP, SP and VS on per capita government health expenditures (GHEpc) in each scenario.

## Results

Across all scenarios, scaling PP was cost saving (1 900–2 300 DALYs averted per 100 000). Scaling SP was cost effective (ICERs $116–268/DALY) and consumed less than 1% of GHEpc. Scaling VS was not cost effective in scenario 1 (ICER $25 800/DALY; 17.5 times GDPpc) or scenario 2 (ICER $49 700/ DALY; 7.4 times GDPpc), but it was cost effective in scenario 3 (ICER $33 200/DALY; 2.6 times GDPpc). The latter result was driven by the lower cost of using existing surgical capacity; however programme expenditures consumed 8.8% of GHEpc.

## Conclusions

This preliminary analysis suggests that PP and SP are cost effective in Africa. The cost effectiveness of building new surgical platforms to scale VS depends highly on programme costs. Future work will assess the cost effectiveness of high-volume surgical platforms serving multiple countries. This model could be used locally as a tool to assist in planning ARF and RHD programmes.

# Abstract: Global demand for cigarettes: a cross-country analysis of consumption, income and price elasticity; 2008–2012

**Authors:** David Watkins, Ruben Conner, Joseph Dieleman, Marie Ng

**Affiliations:** University of Cape Town, Cape Town, South Africa; University of Washington, Seattle, USA; PATH, Seattle, USA; Institute for Health Metrics and Evaluation, University of Washington, Seattle, USA; Institute for Health Metrics and Evaluation, University of Washington, Seattle, USA

## Background and objectives

Tobacco taxation remains an effective but under-utilised means of reducing the burden of smoking-related diseases globally. Estimates of price elasticity of demand (PED), i.e. the ‘sensitivity’ of cigarette use to changes in prices and taxes vary widely across countries and studies, and recent reports suggest that variations in the intensity of cigarette consumption influence price sensitivity. We sought to explore cross-country variations in PED accounting for variations in the relationship between smoking intensity, price and income level.

## Methods

We obtained Global Burden of Disease 2013 estimates of per capita cigarette consumption in 181 countries over five years (2008–2012). We calculated the average price per pack of cigarettes in each country-year using tobacco sales data. We included, as controls, per capita gross domestic product (GDPpc) as a proxy for income as well as indicator variables for the presence of other tobacco policies. We used quantile regression to model PED across varying levels of cigarette consumption, and we assessed statistical significance using robust standard errors.

## Results

We estimated average PED for cigarettes to be –0.15 during 2008–2012, although it was smaller in lower-consumption settings (e.g. –0.11 at the 25th percentile of consumption) and in higher-consumption settings (e.g. –0.12 at the 75th percentile of consumption) compared to moderate-consumption settings (e.g. –0.22 at the median of consumption). We found that PED also varies depending on a country’s level of economic development. Low-income countries were expected to be less price sensitive, while high-income countries were expected to be more price sensitive. Our results were robust to several alternative model specifications.

## Conclusions

Globally, there is significant heterogeneity in PED for cigarettes. Countries where the intensity of smoking is very high or low are less price sensitive than moderate-consumption countries. Furthermore, wealthier countries are more price sensitive than poorer countries. Our findings raise concerns about the relative effectiveness and potentially regressive nature of raising cigarette prices in lower-income, lower-consumption settings (e.g. in sub-Saharan Africa) in the absence of robust non-tax tobacco policies.

# Abstract: Contemporary estimates of morbidity and mortality rates of rheumatic heart disease in South Africa: outcomes from the Cape Town component of the global rheumatic heart disease registry

**Authors:** Liesl Zühlke, ME Engel, BM Mayosi

**Affiliations:** Department of Medicine, Groote Schuur Hospital, Cape Town, South Africa; Department of Medicine, Groote Schuur Hospital, Cape Town, South Africa; Department of Medicine, Groote Schuur Hospital, Cape Town, South Africa

## Introduction

The Global Rheumatic Heart Disease Registry (the REMEDY study) is a prospective study of the baseline characteristics, complications and incidence of sequelae in RHD, and included 531 adults and children from Cape Town.

## Methods

This report describes the characteristics and morbidity estimates at enrolment of patients from two South African sites. We also determined the incidence of adverse cardiovascular events: congestive cardiac failure (CCF), stroke, infective endocarditis, major bleeding, peripheral embolism, rheumatic fever recurrence, hospitalisation, surgery or intervention, pregnancy and all-cause mortality over a 24-month period.

## Results

The RHD patients enrolled at two Cape Town tertiary institutions were young, predominantly female, and largely post-surgical but with a high prevalence of complications at enrolment. Over the follow-up period, we documented an event rate of 203.56 per 1 000 person-years and an annual mortality rate of 4.1% in the Cape Town cohort. The most frequent event in the 24-month period was hospitalisation (13.2%/year) followed by surgery (4.24%/year), CCF (3.86%/year), major bleeding and stroke (1.41/year). Enrolment in cardiac failure (hazard ratio 15.73, 95% CI: 3.94–62.7, *p* < 0.0001) and development of a subsequent episode of CCF conferred the highest risk of mortality (hazard ratio 11.1, 95% CI: 5.6–21.96, *p* = 0.047).

## Conclusion

RHD patients in Cape Town had a mortality rate of 4.1%, which was comparable with the general population. There was a heavy burden of morbid and mortal events with an incidence of 203.56 events per 1 000 person-years. These findings point to the need for targeted interventions to identify and manage at-risk individuals.

# Abstract: Incidence, prevalence and outcome of rheumatic heart disease in South Africa: a systematic review of contemporary studies

**Authors:** Liesl Zühlke, ME Engel, D Watkins, BM Mayosi

**Affiliations:** Department of Medicine, Groote Schuur Hospital, Cape Town, South Africa; Department of Medicine, Groote Schuur Hospital, Cape Town, South Africa; Department of Medicine, Groote Schuur Hospital, Cape Town, South Africa; Department of Medicine, Groote Schuur Hospital, Cape Town, South Africa

## Background

Twenty years after its first democratic election, South Africa is experiencing a health transition. The impact of change on the incidence, prevalence and outcome of rheumatic heart disease (RHD) is unknown.

## Methods

We conducted a systematic overview of the incidence, prevalence and outcomes of RHD in South Africa over the past two decades according to a published protocol.

## Results

The overall crude incidence of symptomatic RHD was 24.7 per 100 000 (95% CI: 22.1–27.4) population per annum among adults (> 13 years) in Soweto, while the prevalence of asymptomatic echocardiographic RHD in schoolchildren was 20.2 cases per 1 000 children (95% CI: 15.3–26.2) in Cape Town. The 60-day mortality after admission with acute heart failure due to RHD was 24.8% (95% CI: 13.6–42.5%) and 180-day mortality was 35.4% (95% CI: 21.6–54.4%). Post-operative mortality at 30 days was 2% (95% CI: 0.0–4%). Post-surgical survival was over 75% at five years, and over 70% at 10 years. Cause-specific mortality rate per 100 000 population decreased from 1.27 (95% CI: 1.17–1.39) in 1997 to 0.7 (95% CI: 0.63–0.78) in 2012.

## Conclusions

The incidence of symptomatic RHD in adults and the prevalence of asymptomatic RHD in schoolchildren are high in South Africa. The mortality rate was high in patients with RHD-related heart failure, although post-surgical morbidity and mortality rates were low. Mortality attributed to RHD may be falling at a population level.

# Abstract: Evaluation of a focused protocol for handheld echocardiography and computer-assisted auscultation in detecting latent rheumatic heart disease in scholars

**Authors:** Liesl Zühlke, ME Engel, S Nkepu, BM Mayosi

**Affiliations:** Department of Medicine, Groote Schuur Hospital, Cape Town, South Africa; Department of Medicine, Groote Schuur Hospital, Cape Town, South Africa; Department of Medicine, Groote Schuur Hospital, Cape Town, South Africa; Department of Medicine, Groote Schuur Hospital, Cape Town, South Africa

## Introduction

Echocardiography is the diagnostic test of choice for latent rheumatic heart disease. The utility of echocardiography for large-scale screening is limited by high cost, complex diagnostic protocols, and time to acquire multiple images. We evaluated the performance of a brief hand-held echocardiography protocol and computer-assisted auscultation in detecting latent rheumatic heart disease with or without pathological murmur.

## Methods

Twenty-seven asymptomatic participants with latent rheumatic heart disease, based on World Heart Federation criteria, and 66 healthy controls were examined by standard cardiac auscultation to detect pathological murmur. Hand-held echocardiography using a focused protocol, which utilises one view (i.e. parasternal long axis) and one measurement (i.e. mitral regurgitant jet) and computer-assisted auscultation utilising an automated decision tool were performed on all participants.

## Results

The sensitivity and specificity of computer-assisted auscultation in latent rheumatic heart disease was 4% (95% CI: 1.0–20.4%) and 93.7% (95% CI: 84.5–98.3%), respectively. The sensitivity and specificity of the focused hand-held echocardiography protocol for definite rheumatic heart disease was 92.3% (95% CI: 63.9–99.8%) and 100%, respectively. The test reliability of hand-held echocardiography was 98.7% for definite and 94.7% for borderline disease, and adjusted diagnostic odds ratios were 1 041 and 263.9 for definite and borderline disease, respectively.

## Conclusion

Computer-assisted auscultation had an extremely low sensitivity but high specificity for pathological murmur in latent rheumatic heart disease. Focused hand-held echocardiography had a fair sensitivity but high specificity and diagnostic utility for definite or borderline rheumatic heart disease in asymptomatic participants.

# Abstract: Precordialgies et syncopes revelant un angor spastique: a propos d’un cas au chu d’angondje a Libreville

**Authors:** Mahutondji Allognon, Ayo Elsa Bivigou, Franck Moubamba

**Affiliations:** Centre Hospitalier Universitiere d’Angondje, Gabon;; Centre Hospitalier Universitiere de Libreville, Gabon; Centre Hospitalier Universitiere d’Angondje, Gabon;

## Introduction

L’angor spastique, depuis sa description effectuée par Prinzmetal, a connu un enrichissement de son expression clinique. Nous rapportons un cas d’angor vasospastique révélé par des précordialgies et des épisodes de syncope.

## Méthodes

cas clinique.

## Résultats

Un homme de 47 ans est admis pour des précordialgies de repos associés à des pertes de connaissance de durée brève et des palpitations. Ces symptômes évolueraient depuis deux mois. Ce patient est à haut risque cardiovasculaire du fait d’une hypertension artérielle suivie depuis cinq ans sous ramipril-hydrochlorothiazide et bisoprolol, d’une obésité majeure et d’une hypercholestérolémie. Pendant l’examen clinique initial, le patient signale l’apparition d’une douleur précordiale à type de constriction, rapidement suivie d’un épisode syncopal devant l’examinateur. L’examen physique postcritique est normal avec une pression artérielle à 120/85 mmHg, une fréquence cardiaque à 75/min. Le tracé électrocardiographique et l’échocardiographie Doppler sont sans particularité. La troponine est négative. L’enregistrement Holter ECG des 24 heures retrouve au moment des épisodes douloureux, des périodes de lésion sous épicardique concomitantes d’un bloc auriculo-ventriculaire du deuxième degré Mobitz 2 avec retour spontané à un tracé normal. Le diagnostic d’angor spastique retenu, un traitement à base d’amlodipine est instauré permettant un amendement complet des symptômes. Il bénéficie plus tard d’une coronarographie objectivant une plaque intermédiaire du tronc commun estimée à 50%. Le test de provocation n’est pas réalisé. L’exploration électrophysiologique est normale. Un antiagrégant plaquettaire et une statine sont rajoutés à l’inhibiteur calcique au décours de la coronarographie. Les recommandations de 2008 de la Japanese Circulation Society nous ont permis de retenir le diagnostic d’angor spastique en présence d’un Holter concluant. Les tests de provocation ne sont indispensables qu’en cas de résultat litigieux ou normal du Holter. Le traitement de première intention repose sur les inhibiteurs calciques parfois associés aux dérivés nitrés. L’évolution chez notre patient après six mois de suivi est favorable.

## Conclusion

L’angor spastique est probablement sous diagnostiqué dans notre pratique quotidienne en Afrique sub-saharienne. Le Holter occupe une place de choix dans la démarche diagnostique.

# Abstract: Infarctus du myocarde inferieur compliquant un non compaction isolee du ventricule gauche: a propos d’un cas

**Authors:** Fatimata Gatta Ba, Houleymata Ba, Mohamed Issa Kharchi, Mohamed Moctar, Abderahmane Bah, Sirakhé Camara

**Affiliations:** Centre National de Cardiologie, Nouakchott, Mauritania; Centre National de Cardiologie, Nouakchott, Mauritania; Centre National de Cardiologie, Nouakchott, Mauritania; Centre National de Cardiologie, Nouakchott, Mauritania; Centre National de Cardiologie, Nouakchott, Mauritania; Centre National de Cardiologie, Nouakchott, Mauritania

## Introduction

Le non compaction du ventricule gauche est une affection congénitale rare caractérisée par la présence de nombreuses trabéculations ventriculaires et de profonds récessus intertrabéculaires. Son association à des anomalies coronaires est peu décrite dans la littérature. Nous rapportons le cas d’un patient porteur d’un non compaction isolée du ventricule gauche qui a présenté un infarctus du myocarde inférieur.

## Méthodes

cas clinique

## Observation

Mr SH âgé de 70 ans, sans facteurs de risque cardio-vasculaire, était suivi pour un non compaction isolée du ventricule gauche de découverte récente, au décours d’un épisode d’oedème aigu du poumon. Il avait présenté une douleur thoracique intense constrictive avec à l’électrocardiogramme une bradycardie sinusale régulière à 55 bpm, un sus décalage du segment ST en inférieur avec une ischémie sous épicardique et onde Q de nécrose. Par ailleurs, on notait un bloc auriculoventriculaire du 1er degré, une hypertrophie auriculaire gauche et ventriculaire gauche.

Il était stable sur le plan hémodynamique, l’examen clinique était normal. La troponine I était positive à 3.57 ug/l. La radiographie du thorax de face montrait une cardiomégalie à 0.66.

L’échocardiographie transthoracique objectivait des cavités gauches dilatées avec akinésie de la paroi inférieure et dysfonction systolique légère. On notait les trabéculations profondes de l’apex et des parois latéro-médiane et latéro-apicale du ventricule gauche avec remplissage des récessus par le flux sanguin et un rapport zone non compactée/zone compactée à 2.4.

La coronarographie avait mis en évidence une lésion intermédiaire au niveau du segment 2 de l’artère coronaire droite et une lésion serrée au niveau du segment 3; le réseau coronaire était normal par ailleurs.

Le patient a été mis sous traitement associant bétabloquants, antiagrégant plaquettaire, diurétiques, inhibiteur de l’enzyme de conversion et statines et a bénéficié d’une angioplastie avec stent actif.

## Conclusion

Le diagnostic de non compaction du ventricule gauche peut être fait par échocardiographie trans thoracique, qui reste l’examen de choix, complétée par l’imagerie par résonnance magnétique cardiaque. Son association à un infarctus du myocarde a rarement été rapportée ; celui-ci pouvant en être une conséquence ou être secondaire à l’athérosclérose.

# Abstract: Le syndrome metabolique: etude multicentrique descriptive en milieu hospitalier cardiologique dakarois

**Authors:** Fatimata Gatta Ba, Malick Bodian, Bamba Ndiaye, Alioune Alassane Ngaide, Maboury Diao, Serigne Abdou Ba

**Affiliations:** Centre National de Cardiologie, Nouakchott, Mauritania; Service de Cardiologie, CHU Aristide Le Dantec, Dakar, Sénégal; Service de Cardiologie, CHU Aristide Le Dantec, Dakar, Sénégal; Service de Cardiologie, CHU Aristide Le Dantec, Dakar, Sénégal; Service de Cardiologie, CHU Aristide Le Dantec, Dakar, Sénégal; Service de Cardiologie, CHU Aristide Le Dantec, Dakar, Sénégal

## Introduction

Le syndrome métabolique, constellation de facteurs de risque, est associé à une incidence accrue de diabète et de maladies cardio-vasculaires. L’objectif de ce travail était de déterminer sa prévalence et ses caractéristiques en milieu hospitalier cardiologique dakarois.

## Méthodes

Nous avons mené une étude transversale et multicentrique, de septembre à novembre, dans 3 services de cardiologie de Dakar. Les patients de 20 ans et plus qui répondaient à la définition du syndrome métabolique selon l’IDF et/ou à la définition harmonisée de 2009 ont été inclus. Les paramètres épidémiologiques, cliniques et paracliniques ont été recueillis.

## Résultats

Nous avons recruté 53 patients qui présentaient un syndrome métabolique selon la définition de l’IDF, d’où une prévalence de 11.8% des hospitalisés et 47.3% des hypertendus. On notait une prédominance féminine avec un sexe ratio de 0.32. L’âge moyen des patients était de 63.1 ans (35 et 89 ans). La pression artérielle élevée (94.3%) et le taux bas de HDL-cholestérol (75.4%) étaient les composantes les plus fréquentes. Soixantesept virgule neuf pour cent des patients ne présentaient que 3 composantes du syndrome. Les patients vivaient en zone urbaine dans 94.3% des cas. Les hommes étaient plus scolarisés que les femmes (*p* = 0.01).

Selon la définition harmonisée, 55 patients étaient concernés d’où une prévalence de 12.3% des hospitalisés. Le sex ratio était de 0.37 en faveur des femmes et l’âge moyen, de 63.3 ans. L’obésité (96.4%) et la pression artérielle élevée (94.5%) étaient plus fréquentes ; L’obésité était retrouvée plus souvent chez les femmes que chez les hommes (p = 0.03). Plus de 3 composantes du syndrome métabolique étaient retrouvées chez 30.9% des patients.

Les maladies cardio-vasculaires associées au syndrome métabolique étaient les coronaropathies (29%) et l’accident vasculaire cérébral (9.1%). L’insuffisance cardiaque était retrouvée chez 40% des patients.

## Conclusion

Le syndrome métabolique n’est pas rare en milieu hospitalier cardiologique. Ses composantes les plus fréquemment retrouvées sont l’hypertension artérielle, l’obésité et l’hypo HDL-cholestérolémie. Parmi les maladies cardio-vasculaires associées à ce syndrome, la maladie coronaire reste prédominante.

# Abstract: Aspects cliniques et paracliniques du diabete au cours du syndrome coronarien aigu: enquete transversale en milieu hospitalier Senegalaise

**Authors:** Kana Babaka, Sayouba Savadogo, Ngoné Diaba Diack, Serigne Cheikh Tidiane Ndao, Sow Dior Diagne, Bouna Diack, Alassane Mbaye, Abdoul Kane

**Affiliations:** Cardiologie de l’hôpital général de Grand Yoff, Sénégal; Cardiologie de l’hôpital général de Grand Yoff, Sénégal; Cardiologie de l’hôpital général de Grand Yoff, Sénégal; Cardiologie de l’hôpital général de Grand Yoff, Sénégal; Cardiologie de l’hôpital général de Grand Yoff, Sénégal; Cardiologie de l’hôpital général de Grand Yoff, Sénégal; Cardiologie de l’hôpital général de Grand Yoff, Sénégal; Cardiologie de l’hôpital général de Grand Yoff, Sénégal

## Introduction

Le diabète est un facteur de risque cardiovasculaire majeur et la survenue d’un syndrome coronarien aigu (SCA) constitue un tournant déterminant dans l’évolution du diabète. L’objectif de cette étude était d’évaluer les aspects épidémiologiques, diagnostiques, thérapeutiques et évolutifs des patients diabétiques présentant un SCA au service de cardiologie de l’hôpital général de Grand Yoff.

## Méthodes

Il s’agit d’une étude rétrospective, descriptive incluant tous les malades hospitalisés pour un SCA de janvier 2013 à avril 2014. Nous avons étudié les caractéristiques du diabète ainsi que les facteurs de risque cardio-vasculaire associés. Les cas d’hyperglycémie réactionnelle ont été exclus de l’étude.

## Résultats

Nous avons colligé 134 patients hospitalisés pour un SCA parmi lesquels 43 présentaient un diabète soit une prévalence de 32%. L’âge moyen était de 63 ans et le sex-ratio de 1.68. Le diabète était inaugural chez ¼ des patients et chez les autres, la durée moyenne d’évolution était de 10 ± 7.4 ans. Il était de type 2 chez 72% des patients. La glycémie à l’entrée était supérieure à 1.80 g/l chez 19 patients et l’HbA1c moyenne était de 9.1%. Parmi les autres facteurs de risque cardio-vasculaire, l’HTA était retrouvée dans 53.5% des cas, la dyslipidémie dans 48% et le tabagisme dans 34.9%. Le diabète était isolé chez 7% des cas. Le délai de consultation était supérieur à 12 heures dans 41% des cas et la douleur thoracique était le symptôme révélateur chez 97% des patients. Il s’agissait d’un SCA avec sus décalage persistant du segment ST chez 38 patients. Une thrombolyse avait été réalisée chez 45% des patients. Une insulinothérapie était instaurée chez 30% des patients diabétiques et dans ¼ des cas aucun traitement antidiabétique n’avait été mis en route durant l’hospitalisation. La durée moyenne d’hospitalisation était de 10 jours avec une mortalité de 18.6%.

## Conclusion

Le diabète est un facteur de risque cardio-vasculaire fréquent chez les patients hospitalisés pour un SCA au service de cardiologie de l’HOGGY. Une amélioration de la prise du diabète pourrait permettre la prévention de cette complication majeure et réduire la forte morbi-mortalité retrouvée chez nos patients.

# Abstract: Prevalence des porteurs de plaques d’atherome carotidiennes et aortiques dans la population semi-rurale de gueoul au Senegal: enquete sur 1 411 sujets

**Authors:** Kana Babaka, Alassane Mbaye, Adama Kane, Diane Djiogap, Bouna Diack, Abdoul Kane

**Affiliations:** Cardiologie CHU Aristide Le Dantec, Dakar, Senegal; l’Hôpital Général de Grand Yoff, Dakar, Senegal; Cardiologie CHU Aristide Le Dantec, Dakar, Senegal; Cardiologie CHU Aristide Le Dantec, Dakar, Senegal; Cardiologie CHU Aristide Le Dantec, Dakar, Senegal; l’Hôpital Général de Grand Yoff, Dakar, Senegal

## Introduction

La prévention des maladies cardio-vasculaires est essentielle et passe entre autres par la détection de l’athérosclérose infra clinique. L’objectif de notre étude était d’étudier le profil et la prévalence des personnes porteuses de plaques d’athérome au niveau carotidien et aortique.

## Méthodes

Il s’agit d’une étude transversale et descriptive sur un mois (03 Novembre – 03 Décembre 2012) incluant les sénégalais âgés d’au moins 35 ans résidant à Guéoul depuis au moins 6 mois ayant accepté volontairement de participer à l’étude. Les femmes enceintes étaient exclues. Les données étaient analysées grâce au logiciel Epi info version 3.5.1. Un seuil de significativité était retenu pour un *p* < 0.05. Le diagnostic était posé par l’écho- Doppler vasculaire.

## Résultats

Sujets (1 411) ont été colligés. L’âge moyen était de 48.5 ans avec une prédominance féminine et un sex ratio de 2.93. La prévalence des facteurs de risque cardio-vasculaire était élevée avec au premier rang la dyslipidémie (61.1%), suivie de la sédentarité (56.1%) et de l’hypertension artérielle (46.4%). La prévalence des porteurs de plaques d’athérome carotidiennes était 6.8%, celle des porteurs de plaques d’athérome aortique était 0.07% et celle des porteurs des plaques d’athérome à la fois carotidiennes et aortiques était 0.3%. Parmi les porteurs de plaques carotidiennes 42.7% avaient des plaques bilatérales. L’âge avancé, le sexe féminin (55%) et l’HTA étaient significativement corrélés à ces plaques ; 50% des porteurs de plaques avaient un artériopathie oblitérante des membres inférieurs et 5.2% des porteurs de plaques carotidiennes avaient des antécédents d’accident vasculaire cérébral.

## Conclusion

Dans notre étude, la prévalence des porteurs de plaques d’athérome carotidiennes et aortiques semble faible. Cependant, l’association avec les différents facteurs de risque cardio-vasculaire est à souligner, justifiant l’importance d’une stratégie de prévention globale des maladies cardio-vasculaires.

# Abstract: Irm cardiaque: luxe ou necessite dans la prise en charge des syndromes angoreux aigus du sujet jeune? A propos de 2 observations

**Authors:** Kana Babaka, Moustapha Sarr, Simon Antoine Sarr, Kharchi Issa Ould, Babacar Mbengue, Malick Bodian, Mouhamadou Bamba Ndiaye, Adama Kane, Maboury Diao, Serigne Abdou Ba

**Affiliations:** Cardiologie CHU Aristide Le Dantec Dakar, Senegal;; Cardiologie CHU Aristide Le Dantec Dakar, Senegal;; Cardiologie CHU Aristide Le Dantec Dakar, Senegal;; Cardiologie CHU Aristide Le Dantec Dakar, Senegal;; Radiologie, Hopital Principal de Dakar, Senegal; Cardiologie CHU Aristide Le Dantec Dakar, Senegal;; Cardiologie CHU Aristide Le Dantec Dakar, Senegal;; Cardiologie CHU Aristide Le Dantec Dakar, Senegal;; Cardiologie CHU Aristide Le Dantec Dakar, Senegal;; Cardiologie CHU Aristide Le Dantec Dakar, Senegal;

## Introduction

La douleur précordiale est un motif fréquent d’admission en cardiologie dont les étiologies peuvent être variées. L’infarctus du myocarde est parfois difficile à différencier de la myocardite aiguë dans sa forme pseudo-infarctoïde. Dans ce contexte, l’imagerie par résonance magnétique (IRM) cardiaque est un outil diagnostique discriminant majeur. Nous rapportons les observations de deux sujets jeunes ayant présenté l’un une myocardite, l’autre un infarctus du myocarde.

## Méthodes

cas clinique.

## Résultats

Il s’agissait de deux patients, l’un âgé de 23 ans tabagique sevré depuis 3 mois avec un syndrome fébrile récent, l’autre âgé de 22 ans footballeur professionnel avec notion de stress sans autres facteurs de risque retrouvés ni de prise de stupéfiants. Ils ont été admis en urgence pour une précordialgie constrictive, intense. L’examen physique était sans particularité. On notait chez les deux patients une élévation des biomarqueurs myocardiques, des troubles de la repolarisation primaire à l’électrocardiogramme et des troubles de la cinétique segmentaire, une dysfonction systolique du ventricule gauche à l’échocardiographie Doppler transthoracique ainsi qu’une coronarographie normale. L’IRM a permis de poser le diagnostic en concluant pour le premier patient à une myocardite aiguë avec akinésie apicale et antérieure, hypersignal T2 franc témoin d’un oedème myocardique et une prise de contraste hétérogène nodulaire respectant les couches sous-endocardiques. Pour le deuxième patient, elle retrouvait un aspect en faveur d’un infarctus aigu étendu antéro-apical avec hypokinésie sévère et rehaussement tardif quasi transmural, une atteinte du pilier antérieur de la valve mitrale. En plus du traitement antalgique, le premier patient a reçu du périndopril et du bisoprolol, le deuxième patient a reçu un traitement anti thrombotique et anticoagulant. L’évolution clinique était favorable.

## Conclusion

L’imagerie par résonance magnétique cardiaque, au-delà de l’électrocardiogramme, est un outil diagnostique discriminant devant une douleur précordiale avec élévation des biomarqueurs myocardiques notamment chez le sujet jeune.

# Abstract: Insuffisance cardiaque au cours de la fibrillation atriale non valvulaire a Brazzaville, Congo

**Authors:** Ellenga Mbolla Bertrand, Rolles Amorce Matingou, Solange Mongo-Ngamami, Christian Kouala-Landa, Mao Ikama, Suzy-Gisele Kimbally-Kaky

**Affiliations:** Service de cardiologie, CHU de Brazzaville, Congo; Service de cardiologie et medecine interne, CHU de Brazzaville, Congo; Service de cardiologie et medecine interne, CHU de Brazzaville, Congo; Service de cardiologie et medecine interne, CHU de Brazzaville, Congo; Service de cardiologie et medecine interne, CHU de Brazzaville, Congo; Service de cardiologie et medecine interne, CHU de Brazzaville, Congo

## But

Déterminer le profil de l’insuffisance cardiaque au cours de la fibrillation atriale non valvulaire (FANV).

## Méthodes

Il s’est agi d’une étude descriptive, réalisée durant 6 mois en 2013, dans le service de cardiologie et médecine interne du CHU de Brazzaville. Etaient inclus, les patients admis pour FANV et ayant réalisé des examens biologiques et morphologiques.

## Résultats

Cinquante patients, d’âge moyen 67.3 ± 12.8 ans (extrêmes: 34 et 88 ans) ont été inclus, dont 27 (54%) femmes. Les antécédents étaient représentés par l’HTA (*n* = 23; 46%) et l’insuffisance cardiaque (*n* = 24). Les signes fonctionnels étaient la dyspnée (*n* = 38; 72%), les palpitations (*n* = 16; 32%) et les signes neurologiques (*n* = 12; 24%). L’insuffisance cardiaque a été notée dans 36 cas (72%) et l’hémiplégie dans 7 cas (14%). A l’échographie cardiaque, la fraction d’éjection était abaissée (< 50%) dans 30 cas (60%) et une HTAP a été rapportée dans 24 cas (46%). Les étiologies principales étaient la cardiopathie hypertensive (30%), la myocardiopathie dilatée (16%) et la cardiopathie ischémique (14%). Le risque thrombotique était élevé dans 39 cas (78%), et le risque hémorragique était modéré dans 35 cas (70%). Un contrôle de la fréquence cardiaque a été effectué chez 40 patients (80%), et un traitement anticoagulant par anti-vitamine K a été utilisé dans 42 cas (84%). Les complications étaient représentées par les AVC (*n*=5) et le syndrome hémorragique sous anti-vitamine K (*n* = 2). Le décès a été enregistré dans 6 cas (12%), et la durée moyenne d’hospitalisation était de 14 ± 6.9 jours (extrêmes: 3 et 34 jours).

## Conclusion

La fréquence de la FANV augmente avec l’émergence des facteurs de risque cardiovasculaires dont l’HTA en milieu africain. Les patients sont pris en charge tardivement, et les anticoagulants oraux directs sont très peu employés.

# Abstract: Maladie thrombo-embolique veineuse: experience du Centre Hospitalier Universitaire de Libreville (CHUL)

**Authors:** Ayo Elsa Bivigou, Mahutondji Christian Allognon, Nzengue Jean-Emmanuel Ecke

**Affiliations:** CHU de Libreville, Gabon;; CHU d’Angondje à Libreville; CHU de Libreville, Gabon;

## Introduction

La maladie veineuse thrombo-embolique (MVTE) semble de plus en plus fréquente en Afrique subsaharienne. Les récentes recommandations internationales nous ont amené à faire le point sur cette pathologie. Cette étude avait pour but d’en décrire les aspects sociodémographiques, diagnostiques thérapeutiques et évolutifs au CHUL.

## Méthodes

Il s’agissait d’une étude rétrospective et descriptive réalisée de janvier 2013 à décembre 2014. Nous avons inclus tous les patients hospitalisés ayant un diagnostic de MVTE, thrombose veineuse profonde (TVP) et/ou embolie pulmonaire (EP), confirmé par échographie Doppler veineux et/ou angioscanner thoracique.

## Résultats

Sur les 768 patients hospitalisés durant cette période, 48 cas de MVTE ont été colligés soit une prévalence de 6.2%. Les TVP concernaient 37 patients (4.8%) et 11 patients présentaient une EP (1.43%). L’âge moyen était de 45 ± 17 ans. Le sex ratio homme/femme était de 0.4. Les facteurs de risque de la MVTE étaient définis en 3 catégories: transitoires majeurs (27%), transitoires mineurs (25%) et étiologiques permanents (63%). L’obésité était le facteur étiologique prédominant (37.5%, *p* < 0.01). La MVTE était idiopathique chez 31.2% des patients. Les D-dimères ont été dosés dans 36.7% des cas. La troponine et le BNP n’ont pas été notifiés. La localisation des TVP proximales était principalement fémoro-poplitée (56.6%). La TVP était distale dans 16% des cas. L’association EP et TVP a été objectivée chez un patient. Une thrombose de la veine basilique a été retrouvée. L’EP a été classée à risque élevé chez deux patients en état de choc. Les scores de probabilité diagnostique de Wells et de Genève ont été utilisés respectivement dans 21 et 9% des cas. L’héparine non fractionnée était initialement administrée chez 44.2% des patients avec relai par fluindione dans 38.7% des cas. La durée moyenne d’hospitalisation était de 15 ± 10.9 jours. Trois patients sont décédés (6.2%) du fait de complications hémorragiques majeures.

## Conclusion

La MVTE concerne une population relativement jeune. L’utilisation des outils diagnostiques et la stratification du risque sont indispensables pour une prise en charge initiale optimale. La prévention chez les sujets à risque reste de rigueur.

# Abstract: La dysplasie arythmogene du ventricule droit avec maladie d’ebstein: une association rare et dangereuse

**Authors:** IB Diop, AD Diallo, M Leye, S Manga, K Regnault, O Dieye, M Dioum, EM Sarr, LL Diene, D Bindia

**Affiliations:** Clinique Cardiologique, CHU Fann, Dakar, Sénégal; Clinique Cardiologique, CHU Fann, Dakar, Sénégal; Clinique Cardiologique, CHU Fann, Dakar, Sénégal; Clinique Cardiologique, CHU Fann, Dakar, Sénégal; Clinique Cardiologique, CHU Fann, Dakar, Sénégal; Clinique Cardiologique, CHU Fann, Dakar, Sénégal; Clinique Cardiologique, CHU Fann, Dakar, Sénégal; Clinique Cardiologique, CHU Fann, Dakar, Sénégal; Clinique Cardiologique, CHU Fann, Dakar, Sénégal; Clinique Cardiologique, CHU Fann, Dakar, Sénégal

## Introduction

La dysplasie arythmogène du ventricule droit est une dégénérescence graisseuse du myocarde ventriculaire droit responsable de troubles du rythme ventriculaire pouvant conduire à la mort subite chez les personnes jeunes et les athlètes.

## Cas clinique

La maladie d’Ebstein est une cardiopathie congénitale rare caractérisée par un défaut de décollement des feuillets de la valve tricuspide. Elle peut être pourvoyeuse de troubles du rythme et insuffisance cardiaque droite. Nous n’avons pas retrouvé dans la littérature une association de ces 2 cardiopathies rares. Nous rapportons le cas d’une patiente de 13 ans adressée dans notre service pour échocardiographie sur des anomalies électriques associées à des signes de décompensation cardiaque droite. La symptomatologie a débuté en décembre 2014 dominée par des palpitations avec secondairement deux épisodes de syncope.

Dans ses antécédents on retrouvait une consanguinité chez les parents avec notion de décès du frère aîné dans le même tableau à l’âge de 15 ans. L’examen physique retrouvait une défaillance cardiaque droite. L’électrocardiogramme révélait une tachycardie sinusale régulière avec une déviation axiale gauche, des ondes Q profondes de V1 à V4, un aspect QS en DII, DIII et aVF et des extrasystoles ventriculaires (ESV) précoces à type de bloc de branche gauche.

L’échographie cardiaque concluait à une maladie d’Ebstein mineure associée à une dilatation importante des cavités droites avec hypokinésie de la paroi antérieure du ventricule droit et de l’infundibulum évocateurs d’une dysplasie arythmogène du ventricule droit. Le Holter ECG a confirmé l ’hyper excitabilité ventriculaire à type de bloc de branche gauche avec des salves de tachycardie ventriculaire (TV). Devant ce tableau une indication d’intervention de Conn associée à un défibrillateur automatique implantable a été posée. Sa prise en charge était prévue après transfert en Europe.

L’évolution a été marquée par une persistance des signes de congestion droite malgré le traitement diurétique maximal et surtout des salves de TV malgré un traitement anti-arythmique (amiodarone et béta bloquant). Le décès est survenu brutalement en cours d’hospitalisation, 6 mois après l’apparition des symptômes.

## Conclusion

Il s’agit à notre connaissance du premier cas d’association de 2 pathologies rares avec un potentiel évolutif pouvant être grave voire mortel dans notre observation. Etant donné les troubles du rythme ventriculaires graves le défibrillateur implantable aussitôt que possible reste la meilleure alternative efficace. La chirurgie de la maladie d’Ebstein est envisageable dans certaines formes anatomiques avec des résultats encourageants.

# Abstract: Cardiologie interventionnelle au centre de coronarographie du chu de fann (DAKAR) experience preliminaire. A propos de 173 cas: resultats, difficultes et perspectives

**Authors:** IB Diop, S Manga, K Regnault, A Alanti, M Dioum, M Leye, EHM Sarr, A Diallo, L Diene

**Affiliations:** Clinique Cardiologique, CHU Fann, Dakar, Senegal; Clinique Cardiologique, CHU Fann, Dakar, Senegal; Clinique Cardiologique, CHU Fann, Dakar, Senegal; Clinique Cardiologique, CHU Fann, Dakar, Senegal; Clinique Cardiologique, CHU Fann, Dakar, Senegal; Clinique Cardiologique, CHU Fann, Dakar, Senegal; Clinique Cardiologique, CHU Fann, Dakar, Senegal; Clinique Cardiologique, CHU Fann, Dakar, Senegal; Clinique Cardiologique, CHU Fann, Dakar, Senegal

## Introduction

La cardiologie interventionnelle était presque inexistante en Afrique sub-Saharienne alors que les maladies cardio-vasculaires constituent la première cause de mortalité. Nos patients étaient transférés dans les pays du Nord. Nous rapportons notre première année d’expérience en cardiologie interventionnelle.

## Méthodes

Nous avons réalisé une analyse rétrospective des coronarographies et angioplasties réalisées au Centre de Coronarographie du Centre Hospitalier Universitaire de Fann de Dakar entre Avril 2013 et Mars 2015. L’équipement utilisé était un Siemens Axiom Artis. Les données cliniques, paracliniques, angiographiques et évolutives ont été recueillies et analysées.

## Résultats

Durant cette période de 23 mois, 173 patients ont bénéficié d’une coronarographie. Le sexe ratio était de 3.35. L’âge moyen était de 62.84 ± 10.49 ans. L’hypertension artérielle était le principal facteur de risque cardio-vasculaire retrouvé (52.02%), suivie de la dyslipidémie (43.35%), du tabagisme (34.68%) et du diabète de type II (24.27%) des cas. Quarante-quatre pourcent des patients avaient déjà présenté un infarctus du myocarde. La demande de coronarographie était motivée dans 32.94% des cas par un angor d’effort et par une dyspnée d’effort dans 15.6% des cas. Un test d’effort a été réalisé chez 10.98% des patients et était positif dans 84.21% des cas. L’électrocardiogramme était anormal dans 85.54%. L’écho-Doppler cardiaque trans-thoracique montrait une fraction d’éjection ventriculaire gauche altérée dans 37.57 % des cas. L’abord fémoral a été utilisé chez 93.64% des patients. Le produit de contraste utilisé était l’Ioxitalamate de méglumine dans 88.43% des cas. La durée moyenne d’exposition aux radiations était de 11 min 02 s. L’artère responsable du syndrome coronarien était l’inter ventriculaire antérieur (IVA) dans 67.63% des cas. L’indication thérapeutique posée était l’angioplastie dans 39.75% des cas, le traitement médical dans 21.68% des cas et le pontage aorto-coronaire chez 15.06% des patients. Quarante angioplasties ont été réalisées avec pose de stents avec un bon résultat. Un patient est décédé en cours de procédure (dissection extensive de la cx).

## Conclusion

Notre expérience montre qu’en Afrique, il est possible d’implanter des salles de cardiologie interventionnelle permettant de réduire la morbi-mortalité coronarienne et de résoudre le problème des évacuations sanitaires.

# Abstract: Cardiopathies neonatales et urgences neonatales: difficultes de detection et de prise en charge precoce au Senegal

**Authors:** IB Diop, M Leye, K Regnault, AD Diallo, O Dieye, EHM Sarr, S Manga, LL Diene, M Dioum, D Bindia

**Affiliations:** Clinique Cardiologique, CHU Fann, Dakar, Senegal; Clinique Cardiologique, CHU Fann, Dakar, Senegal; Clinique Cardiologique, CHU Fann, Dakar, Senegal; Clinique Cardiologique, CHU Fann, Dakar, Senegal; Clinique Cardiologique, CHU Fann, Dakar, Senegal; Clinique Cardiologique, CHU Fann, Dakar, Senegal; Clinique Cardiologique, CHU Fann, Dakar, Senegal; Clinique Cardiologique, CHU Fann, Dakar, Senegal; Clinique Cardiologique, CHU Fann, Dakar, Senegal; Clinique Cardiologique, CHU Fann, Dakar, Senegal

## Introduction

La clinique Cardiologique du CHU Fann de Dakar constitue le principal Centre de Référence des Cardiopathies Congénitales au Sénégal. L’objectif de ce travail est de montrer la prévalence des cardiopathies congénitales en période néonatale au sein de la consultation d’échocardiographie, de déterminer les motifs des demandes d’exploration, les pathologies détectées ainsi que les problèmes induits par cette détection.

## Méthodes

Nous avons réalisé une étude rétrospective de janvier 2010 à mars 2015, nous nous sommes intéressés à l’âge, la provenance, le motif de consultation le diagnostic écho cardiographique et ses implications thérapeutiques et évolutives.

## Résultats

Durant la période d’étude 121 nouveau-nés ont été reçus en consultation (1% des patients explorés). L’âge moyen était de 13.7 ± 6.8 jours avec un sexe ratio de 0.35. Ils étaient essentiellement référés pour bilan d’une malformation extracardiaque (41%) et pour l’évaluation d’un souffle cardiaque (19%). Ces nouveaux nés provenaient en majorité de l’Hôpital d’Enfants Albert Royer (51%). Les communications interventriculaires (CIV) (11%) et les communications inter auriculaires (CIA) (9%) étaient les plus fréquentes suivies de la persistance du canal artériel (PCA) (4%) de la tétralogie de Fallot (4%) et des canaux atrio-ventriculaires (4%). La transposition des gros vaisseaux représentait 2.4% des cas, l’hypoplasie du ventricule gauche 1.6%. Un coeur normal était confirmé dans 48% des cas.

## Conclusions

Les cardiopathies détectées en période néonatale restent dominées par les communications interventriculaires et les CIA. La prévalence de la transposition des gros vaisseaux reste faible témoignant d’une sous détection et d’une mortalité néonatale élevée. Le dépistage et la prise en charge précoce devraient être améliorés par l’examen systématique à la naissance et un relèvement du plateau technique médico chirurgical permettant l’établissement d’une chaine de soins complète.

# Abstract: Dilatation mitrale percutanee: experience inaugurale au Senegal

**Authors:** IB Diop, K Regnault, EM Sarr, M Dioum, S Manga, M Leye, AD Diagne, LL Diene, D Bindia

**Affiliations:** Clinique Cardiologique, CHU Fann, Dakar, Sénégal; Clinique Cardiologique, CHU Fann, Dakar, Sénégal; Clinique Cardiologique, CHU Fann, Dakar, Sénégal; Clinique Cardiologique, CHU Fann, Dakar, Sénégal; Clinique Cardiologique, CHU Fann, Dakar, Sénégal; Clinique Cardiologique, CHU Fann, Dakar, Sénégal; Clinique Cardiologique, CHU Fann, Dakar, Sénégal; Clinique Cardiologique, CHU Fann, Dakar, Sénégal

## Introduction

Le rétrécissement mitral rhumatismal reste une cardiopathie fréquente au Sénégal. Son traitement a été modifié par le développement de la commissurotomie mitrale percutanée (CMP). Notre objectif était de rapporter notre première expérience de CMP et d’en évaluer les résultats à court terme.

## Méthodes

Cette étude portait sur une série de quatorze patients porteurs de rétrécissement mitral serré symptomatique avec anatomie favorable ayant bénéficié d’une CMP. L’indication était posée sur l’anatomie valvulaire et la sévérité de la sténose appréciées à l’écho-Doppler cardiaque. La dilatation mitrale était réalisée sous anesthésie locale ou générale par voie veineuse fémorale et trans septale au moyen d’un ballon d’Inoue en salle de cathétérisme cardiaque (Siemens ARTIS).

## Résultats

L’âge moyen était de 23 ± 6.77 ans. Le sex-ratio était de 0.2. La majorité des patients avait une dyspnée stade II (NYHA). Le diamètre moyen du ballon était de 27 ± 1.2 mm. A l’écho-Doppler post procédure, on notait une augmentation de la surface mitrale (SM) de 0.67 ± 0.09 à 1.7 ± 0.13 cm2 et une diminution du gradient moyen et des pressions pulmonaires systoliques (PAPS) respectivement de 17 ± 5.4 à 7.3 ± 2.9 mmHg et de 107 ± 18.6 à 49 ± 24.9 mmHg. Un échec de dilatation était noté par défaut de passage du ballon de l’OG vers le VG. La majorité des patients avait une fuite grade I et II en post procédure. Au contrôle à 3 mois, la majorité de nos patients était nettement améliorée au plan fonctionnel avec stabilité des données échocardiographiques.

## Conclusion

la CMP est devenue un traitement de choix du rétrécissement mitral. La sélection des patients reste primordiale basée sur des critères anatomo-cliniques et fonctionnels bien codifiés.

# Abstract: Le syndrome de holt oram: a propos de deux cas a la clinique cardiologique du chu de fann, Dakar

**Authors:** IB Diop, AD Diallo, M Leye, S Manga, EM Sarr, K Regnault, O Dieye, M Dioum, LL Diene, D Bindia

**Affiliations:** Clinique Cardiologique, CHU Fann, Dakar, Sénégal; Clinique Cardiologique, CHU Fann, Dakar, Sénégal; Clinique Cardiologique, CHU Fann, Dakar, Sénégal; Clinique Cardiologique, CHU Fann, Dakar, Sénégal; Clinique Cardiologique, CHU Fann, Dakar, Sénégal; Clinique Cardiologique, CHU Fann, Dakar, Sénégal; Clinique Cardiologique, CHU Fann, Dakar, Sénégal; Clinique Cardiologique, CHU Fann, Dakar, Sénégal; Clinique Cardiologique, CHU Fann, Dakar, Sénégal; Clinique Cardiologique, CHU Fann, Dakar, Sénégal

## Introduction

Le syndrome de Holt-Oram, encore appelé syndrome Main-Coeur, est un trouble héréditaire rare caractérisé par une malformation complexe du coeur et des membres supérieurs. Le plus souvent, il existe des anomalies dans les os du carpe du poignet et dans les os du pouce en même temps que des malformations cardiaques tels que des communications interauriculaire (CIA) ou ventriculaire (CIV). Nous rapportons deux cas de syndrome de Holt-Oram.

## Méthodes

cas cliniques (2).

## Observation 1

Jeune garçon de 12 ans adressé dans notre service pour CIV de découverte systématique. Dans ses antécédents on retrouvait une chirurgie de hernie inguino-scrotale droite et de l’avant-bras droit. L’examen physique révélait une agénésie bilatérale du pouce avec atrophie du membre supérieur droit et un souffle systolique mésocardiaque. L’échocardiographie a posé le diagnostic de CIV périmembraneuse large avec sténose valvulaire pulmonaire moyenne. Il a bénéficié d’une fermeture chirurgicale de sa CIV avec un gradient infundibulaire résiduel pulmonaire à 75 mmHg. La mise sous béta bloquant a permis en 6 mois une réduction du gradient jusqu’à 19 mmHg. Actuellement le patient est asymptomatique sans traitement.

## Observation 2

Enfant de 3 ans adressé pour CIA découverte dans le cadre du bilan de bronchites à répétition. On retrouve une notion de consanguinité entre les parents. L’examen physique retrouvait un souffle systolique endapexien avec une décompensation cardiaque globale et une agénésie bilatérale du pouce et du radius. L’échographie cardiaque retrouvait une CIA ostium secundum large avec un septum interauriculaire multi perforé et un retentissement important sur les cavités droites. Le patient est actuellement en attente d’une fermeture chirurgicale de sa CIA.

## Conclusion

Nous avons reporté deux cas de syndrome de Holt Oram avec des manifestations cardiaques différentes. Il s’agit d’une association rarement reportée en Afrique qu’il faut savoir rechercher devant toute anomalie osseuse des membres supérieurs en particulier du rayon radial.

# Abstract: Problematique de la prise en charge chirurgicale des cardiopathies congenitales au Senegal. Cas operes par les missions humanitaires localement au Senegal de 2010 a 2015: indications, resultats et perspectives

**Authors:** IB Diop, AD Diallo, M Leye, --- ---, --- ---, --- ---, --- ---, --- ---, --- ---, --- ---, --- ---, --- ---, --- ---, --- ---, --- ---

**Affiliations:** Clinique Cardiologique, CHU Fann, Dakar, Sénégal; Clinique Cardiologique, CHU Fann, Dakar, Sénégal; Clinique Cardiologique, CHU Fann, Dakar, Sénégal; Clinique Cardiologique, CHU Fann, Dakar, Sénégal; Clinique Cardiologique, CHU Fann, Dakar, Sénégal; Clinique Cardiologique, CHU Fann, Dakar, Sénégal; Clinique Cardiologique, CHU Fann, Dakar, Sénégal; Clinique Cardiologique, CHU Fann, Dakar, Sénégal; Clinique Cardiologique, CHU Fann, Dakar, Sénégal; Clinique Cardiologique, CHU Fann, Dakar, Sénégal; Clinique Cardiologique, CHU Fann, Dakar, Sénégal; Clinique Cardiologique, CHU Fann, Dakar, Sénégal; Clinique Cardiologique, CHU Fann, Dakar, Sénégal; Clinique Cardiologique, CHU Fann, Dakar, Sénégal; Clinique Cardiologique, CHU Fann, Dakar, Sénégal

## Introduction

Les cardiopathies congénitales sont de plus en plus nombreuses au Sénégal du fait d’un diagnostic de plus en plus précoce. Cependant le principal problème demeure dans leur prise en charge car la chirurgie à coeur ouvert reste encore très peu accessible. Dans un pays où les ressources sont très limitées en l’absence d’autonomie complète des équipes locales et d’équipements adéquats, la prise en charge chirurgicale des enfants se fait essentiellement grâce aux humanitaires. Elle peut être locale pour les cardiopathies les plus simples au cours de missions ponctuelles ou par transfert dans des centres européens spécialisés pour les cas les plus complexes.

## Objectif

Le but de ce travail était d’évaluer la prise en charge chirurgicale des patients durant les missions humanitaires qui se sont déroulés au service de chirurgie thoracique et cardiovasculaire de l’hôpital Fann de Janvier 2010 à Juin 2015, d’analyser les indications, les résultats et les perspectives.

## Méthodes

Nous avons colligés 68 cas de patients ayant bénéficié de chirurgie de leur cardiopathie congénitale durant l’une des 13 missions humanitaires réalisées dans cette période. L’âge moyen était de 7.8 ans avec des extrêmes à 11 jours et 25 ans. En moyenne 5 patients étaient opérés à chaque mission.

## Résultats

Les principales indications retrouvées étaient la tétralogie de Fallot dans 50% des cas, la communication interauriculaire dans 17.6% des cas et la communication interventriculaire dans 14.7% des cas. Ces cardiopathies pouvaient être isolées ou associées. Les patients avaient bénéficié d’une cure complète dans 95.5% des cas et d’une chirurgie palliative dans 5.8% des cas; 4 cas de décès ont été enregistrés au total sur les 6 ans. Cependant, ces patients opérés représentaient 31% des patients qui avaient une indication opératoire au moment de ces missions.

## Conclusion

Ce qui montre que ces missions ne peuvent résorber qu’une infime partie des patients en attente d’une chirurgie. Pour régler définitivement ces problèmes il faut dès lors réfléchir sur les possibilités de développement de la chirurgie locale.

# Abstract: MALADIE CORONAIRE CHEZ LE PATIENT DIABETIQUE: ASPECTS CLINIQUES, PARACLINIQUES ET DIFFICULTES THERAPEUTIQUES

**Authors:** M Dioum, IB Diop, K Regnault, S Manga, MCBO Leye, EM Sarr, AD Diagne, LL Diene, D Bindia

**Affiliations:** Clinique Cardiologique, CHU Fann, Dakar, Sénégal; Clinique Cardiologique, CHU Fann, Dakar, Sénégal; Clinique Cardiologique, CHU Fann, Dakar, Sénégal; Clinique Cardiologique, CHU Fann, Dakar, Sénégal; Clinique Cardiologique, CHU Fann, Dakar, Sénégal; Clinique Cardiologique, CHU Fann, Dakar, Sénégal; Clinique Cardiologique, CHU Fann, Dakar, Sénégal; Clinique Cardiologique, CHU Fann, Dakar, Sénégal; Clinique Cardiologique, CHU Fann, Dakar, Sénégal

## Introduction

La pathologie coronarienne représente une cause majeure de décès cardio-vasculaire chez les diabétiques. L’objectif de cette étude était de décrire les aspects diagnostiques et les difficultés thérapeutiques rencontrées chez les coronariens diabétiques.

## Methodes

Nous avons réalisé une étude rétrospective descriptive couvrant la période de juin 2013 à mai 2015. Nous avons inclus tous les patients diabétiques connus ou de découverte récente ayant bénéficié d’une coronarographie et/ou d’une angioplastie dans notre Centre. La saisie des données a été faite sur Excel et l’analyse statistique a été faite par le logiciel Stata IC 12.

## Résultats

Nous avons inclus 36 patients. La prévalence hospitalière était de 21%. L’âge moyen était de 63 ± 9.6 ans. Le sex ratio était de 2. Les facteurs de risque associés au diabète étaient dominés par l’hypertension artérielle (80%) suivie de la dyslipidémie (44%) du tabagisme et de l’obésité respectivement (27 et 25%). Plus d’un quart de nos patients avait eu un antécédent d’infarctus du myocarde. Tous les patients étaient sous antidiabétiques oraux et trois parmi eux étaient sous insuline. La symptomatologie clinique était dominée par l’angor d’effort (44%) et 25% des patients étaient asymptomatiques. Sur le plan biologique, la glycémie à jeun moyenne était de 1.62 ± 0.6 g/l (0.98–2.92) et le taux moyen d’hémoglobine glyquée de 6.5% (4.2–9.8). L’électrocardiogramme retrouvait des anomalies du segment ST et de l’onde T dans 61% des cas. La fraction d’éjection du ventricule gauche moyenne était de 54% (28–76). Au plan angiographique, l’atteinte du réseau coronaire gauche était plus fréquente (88%). L’atteinte était mono-tronculaire dans 27% des cas, bi-tronculaire dans 22% des cas et tri-tronculaire dans 25% des cas. Dans 26% des cas, la coronarographie était normale. Huit patients (22.2%) avaient bénéficié d’une angioplastie coronaire dont 6 avec des stents actifs et 2 avec des stents nus. Deux patients ont été traités par pontage aortocoronaire. Un traitement médical avait été retenu chez 72% des patients du fait de leur âge avancé, de l’aspect anatomique des lésions coronariennes, mais aussi des problèmes chirurgicaux et financiers.

## Conclusion

Chez les diabétiques l’atteinte pluri-tronculaire est fréquente. La prise en charge thérapeutique reste difficile en raison essentiellement de l’inaccessibilité des moyens thérapeutiques d’où la nécessité d’un contrôle strict de tous les facteurs de risque cardio-vasculaire et d’une collaboration entre cardiologue et diabétologue. 

# Abstract: TV fasciculaire reduite par inhibiteurs calciques chez une femme enceinte

**Authors:** Abdelhak Fodha, Mlayah Dorsaf, Mejdi Ben Messoud, Abdallah Wajih, Beccouche Mohamed, Majed Hassine, Betbout Fethi, Dridi Zohra, Gamra Habib

## Introduction

Les tachycardies ventriculaires (TV) chez la femme enceinte peuvent se voir sur une anomalie structurale cardiaque ou révélant un trouble de rythme congénital non diagnostiqué. Il n’est cependant pas rare d’avoir une TV gravidique sur coeur sain.

## Méthodes

cas clinique

## Observation

Nous rapportons le cas d’une femme âgée de 24 ans, enceinte à 22 SA, qui consulte aux urgences pour des crises de palpitations récurrentes plutôt bien tolérées. Sa grossesse est bien suivie et de déroulement normale. Sur son tracé électrocardiographique, on enregistre une tachycardie régulière à QRS peu élargis avec aspect de bloc de branche droit et axe gauche évoquant un hémi bloc antérieur gauche évoquant une tachycardie ventriculaire bénigne fasciculaire de Belhassen. Les digitaliques et les manoeuvres vagales faites n’ont rien donné, de même que l’amiodarone malgré une dose de charge en IV puis un relais par voie orale pendant 24 heures.

Cette arythmie présente la caractéristique d’être sensible aux inhibiteurs calciques qui ont la particularité de ralentir la tachycardie avant de l’arrêter, chose produite dans notre cas. La patiente n’a pas refait de tachycardie depuis cet incident sous couverture d’inhibiteurs calciques. L’échographique cardiaque faite n’a pas retrouvé d’anomalies. Nous envisageons après son accouchement de lui faire une exploration électro-physiologique et éventuellement une ablation par radiofréquence.

## Conclusion

Les TV idiopathiques fasciculaires au cours de la grossesse répondant aux inhibiteurs calciques sont rares est de diagnostic souvent difficile. Leur pronostic est généralement bon et sont accessibles à une ablation endocavitaire.

# Abstract: Cardiomyopathies dilatees: suivi evolutif sur 2 ans et facteurs pronostiques au chu sylvanus olympio de lome

**Authors:** Akue E Goeh, M Pio, C Kengne, S Baragou, Y Afassinou, S Péssinaba, KE Mossi, B Atta, K Ehlan, F Damorou

**Affiliations:** Service de Cardiologie, Centre Hospitalier Universitaire Sylvanus Olympio (CHU SO) de Lomé, Togo; Service de Cardiologie, Centre Hospitalier Universitaire Sylvanus Olympio (CHU SO) de Lomé, Togo; Service de Cardiologie, Centre Hospitalier Universitaire Sylvanus Olympio (CHU SO) de Lomé, Togo; Service de Cardiologie, Centre Hospitalier Universitaire Campus de Lomé, Togo; Service de Cardiologie, Centre Hospitalier Universitaire Sylvanus Olympio (CHU SO) de Lomé, Togo; Service de Cardiologie, Centre Hospitalier Universitaire Campus de Lomé, Togo; Service de Cardiologie, Centre Hospitalier Universitaire Sylvanus Olympio (CHU SO) de Lomé, Togo; Service de Cardiologie, Centre Hospitalier Universitaire Sylvanus Olympio (CHU SO) de Lomé, Togo; Service de Cardiologie, Centre Hospitalier Universitaire Campus de Lomé, Togo

## Introduction

La cardiomyopathie dilatée (CMD) est l’atteinte cardiaque la plus fréquente au Togo.

## Objectifs

Analyser l’évolution des CMD sur une période de 2 ans et dégager les facteurs pronostiques.

## Méthodes

Il s’est agi d’une étude longitudinale descriptive allant de janvier 2010 à janvier 2014. Elle a été réalisée dans le service de cardiologie du CHU SO de Lomé. Une première phase était rétrospective et avait consisté à la sélection des dossiers des patients hospitalisés pour CMD du 1er janvier 2010 au 31 décembre 2011. La deuxième phase était prospective et avait consisté à suivre les patients du 1er janvier 2012 au 1er janvier 2014.

## Résultats

La prévalence des CMD était de 25.4%. L’âge moyen des patients était de 50.6 ± 16.8 ans. Le sex-ratio H/F était de 0.72. L’insuffisance cardiaque globale était le principal mode de décompensation (74.9%). L’origine hypertensive était la plus observée (26.2%). La durée moyenne d’hospitalisation était de 12.8 ± 16.6 jours. La mortalité était de 7.3% en hospitalisation. La mortalité à 3 mois, à 6 mois, à 1 et 2 ans de suivi était respectivement de 11.5, 16.7, 24, 40.8%. Les facteurs de mauvais pronostic étaient: sexe féminin, âge ≥ 55 ans, présence d’extrasystoles ventriculaires, DTDVG ≥ 70 mm, FEVG ≤ 25%, HTAP ≥ 60 mmHg, présence d’un thrombus intra cavitaire, l’origine hypertensive, ischémique et virale (VIH).

## Conclusion

La survie à 2 ans des CMD est faible au Togo due à la sévérité des lésions à l’admission et à une mauvaise observance thérapeutique.

# Abstract: Hypercholestrolemie familiale homozygote compliquee de coronaropathie severe: a propos d’un cas

**Authors:** G Hachachna, Mokhtar O Ait, M Saidane, S Latreche, S Benkhedda

**Affiliations:** Clinique Cardiologique, CHU Mustapha, Alger, Algerie; Clinique Cardiologique, CHU Mustapha, Alger, Algerie; Clinique Cardiologique, CHU Mustapha, Alger, Algerie; Clinique Cardiologique, CHU Mustapha, Alger, Algerie; Clinique Cardiologique, CHU Mustapha, Alger, Algerie

## Introduction

L’hypercholestérolémie familiale homozygote est une maladie rare touchant environ un individu sur un million. Elle est mortelle en l’absence de traitement l’hypercholestérolémie homozygote est le plus souvent due à une mutation homozygote du gène LDLR (plus souvent en cas de mariage consanguin). Elle est caractérisée cliniquement par la présence de dépôts cutanés de cholestérol qui permettent de faire le diagnostic dans les premières années de la vie. L’atteinte cardiaque est caractérisée par un véritable envahissement de la racine de l’aorte par des dépôts de cholestérol réalisant une sténose supra valvulaire souvent calcifiée et rapidement evolutive. Cette athérosclérose véritablement maligne, peut englober les ostias coronaires sur le plan biologique. La cholestérolémie totale est en général comprise entre 6 et 15 g/l. Pour le traitement LDL-aphérèse et les statines ont montré leur efficacité dans ce cas.

## Méthodes

cas clinique. Nous rapportons une observation d’une jeune femme de 28 ans dont le bilan de recherche d’une atteinte cardiaque a trouvé des lésions tri tronculaires ratées par PCI.

# Abstract: Ablation par radiofrequence du flutter atrial: a propos des 3 premiers cas realises au Senegal

**Authors:** RG Houndolo, A Kane, E Houensou, K Babaka, SA Sarr, P Defaye, I Kouame, M Bodian, MB Ndiaye, M Diao, M Sarr, SA Ba

**Affiliations:** Clinique Cardiologique, Hopital Aristide Le Dantec, Dakar, Sénégal; Clinique Cardiologique, Hopital Aristide Le Dantec, Dakar, Sénégal; Clinique Cardiologique, Hopital Aristide Le Dantec, Dakar, Sénégal; Clinique Cardiologique, Hopital Aristide Le Dantec, Dakar, Sénégal; Clinique Cardiologique, Hopital Aristide Le Dantec, Dakar, Sénégal; Clinique Cardiologique, Hopital Aristide Le Dantec, Dakar, Sénégal; Clinique Cardiologique, Hopital Aristide Le Dantec, Dakar, Sénégal; Clinique Cardiologique, Hopital Aristide Le Dantec, Dakar, Sénégal; Clinique Cardiologique, Hopital Aristide Le Dantec, Dakar, Sénégal; Clinique Cardiologique, Hopital Aristide Le Dantec, Dakar, Sénégal; Clinique Cardiologique, Hopital Aristide Le Dantec, Dakar, Sénégal; Clinique Cardiologique, Hopital Aristide Le Dantec, Dakar, Sénégal

## Introduction

Le flutter atrial constitue un trouble du rythme fréquent et grave engageant le pronostic fonctionnel et vital du sujet à cause du risque thromboembolique. Il peut survenir sur coeur sain ou pathologique. Malgré le traitement médical et les cardioversions électriques ou chimiques, les récidives sont fréquentes. L’ablation par radiofréquence est une technique moderne curative du flutter atrial. Nous rapportons 3 observations à propos des 3 premiers patients ayant bénéficié d’une ablation de flutter atrial au Sénégal.

## Méthodes

cas cliniques (3).

## Observation

Le premier patient était âgé de 26 ans et porteur d’une cardiomyopathie dilatée hypokinétique depuis 10 ans. Il a présenté 2 ans auparavant un accident un accident vasculaire cérébral ischémique au décours duquel le diagnostic de flutter avait été posé. Il a subi 3 cardioversions électriques qui se sont avérées être des échecs. Il était sous traitement anti arythmique (cordarone 200 mg/j et bisoprolol 10 mg/j et anticoagulant oral. L’ECG de départ montre un flutter atrial à conduction variable et une hypertrophie ventriculaire gauche systolique. Le deuxième patient était âgé de 62 ans et présentait un flutter atrial à conduction fixe 2/1 sur coeur sain. Il était sous traitement de base de bisoprolol 10 mg/j et anticoagulant oral. Le troisième patient était âgé de 46 ans et avait comme antécédent un infarctus du myocarde datant de 6 mois. Il présentait une cardiopathie ischémique et un flutter atrial depuis 3 mois. L’ECG de départ montrait un rythme sinusal avec une nécrose séquellaire en antérieur. Il était également sous bisoprolol 10 mg/j et traitement anticoagulant oral. Pour les 3 patients, la procédure d’ablation s’est avérée être un succès et s’est déroulée sans incident ni accident. Aucun des patients n’a présenté de complications après la procédure. L’ECG post ablation montre un rythme sinusal. Les patients sont asymptomatiques et restent en rythme sinusal avec un recul moyen de 3 mois.

## Conclusion

L’ablation par radiofréquence est une technique curative du flutter atrial simple. Elle est aujourd’hui accessible au Sénégal. Elle permet d’améliorer le pronostic du patient.

# Abstract: Evaluation de la stimulation cardiaque definitive a l’hôpital Aristide le Dantec a Dakar

**Authors:** RG Houndolo, A Kane, E Houensou, S Savadogo, SA Sarr, F Taqseft, M Bodian, MB Ndiaye, M Diao, M Sarr, SA Ba

**Affiliations:** Clinique Cardiologique, Hôpital Aristide Le Dantec, Dakar, Sénégal; Clinique Cardiologique, Hôpital Aristide Le Dantec, Dakar, Sénégal; Clinique Cardiologique, Hôpital Aristide Le Dantec, Dakar, Sénégal; Clinique Cardiologique, Hôpital Aristide Le Dantec, Dakar, Sénégal; Clinique Cardiologique, Hôpital Aristide Le Dantec, Dakar, Sénégal; Clinique Cardiologique, Hôpital Aristide Le Dantec, Dakar, Sénégal; Clinique Cardiologique, Hôpital Aristide Le Dantec, Dakar, Sénégal; Clinique Cardiologique, Hôpital Aristide Le Dantec, Dakar, Sénégal; Clinique Cardiologique, Hôpital Aristide Le Dantec, Dakar, Sénégal; Clinique Cardiologique, Hôpital Aristide Le Dantec, Dakar, Sénégal; Clinique Cardiologique, Hôpital Aristide Le Dantec, Dakar, Sénégal

## Introduction

Les porteurs de pacemaker sont de plus en plus nombreux dans le monde, avec des indications de plus en plus en plus larges. Au Sénégal et en Afrique la stimulation cardiaque est en plein essor. La possibilité de complications redoutables impose des précautions au cours de l’implantation et la nécessité d’une surveillance adéquate et régulière. Dans ce travail nous évaluons la pratique de la stimulation cardiaque à l’Hôpital Aristide Le Dantec.

## Méthodes

Il s’agit d’une étude rétrospective allant de janvier 2004 à décembre 2008 concernant une population de patients ayant bénéficié d’une stimulation cardiaque définitive à la clinique cardiologique de l’Hôpital Aristide Le Dantec.

## Résultats

Notre étude portait sur une population de 172 patients. La prévalence était de 4.5%. L’âge moyen était de 70 ans avec des extrêmes de 21 et 96 ans. Le niveau socio-économique des patients était: moyen (60.5%); bon (25%); bas (14.5)% ; 52.3% des patients étaient de sexe féminin et 47.7% de sexe masculin. Les symptômes retrouvés étaient: vertiges (66.3%), dyspnée (48.8%), syncope (43.6%). Les patients asymptomatiques représentaient 2.9%. Les anomalies électrocardiographiques retrouvées étaient: BAV III (79.1%), BAV haut degré (15.1%), BAVII (1.2%), tri bloc (2.9%), bloc sino-auriculaire (1.2%), maladie de l’oreillette (0.6%). Les étiologies étaient: dégénérative (69.1%), myocardiopathies (6.4%), endocardite infectieuse (0.6%), cause inconnue (20.4%). L’antibioprophylaxie était prescrite pour tous les patients à base de céphalosporine de 3ème génération. Une stimulation temporaire a été faite chez 16.3% des patients. La voie sous-clavière pour la stimulation définitive a été utilisée chez 95.3% des patients. Une pile neuve était utilisée chez 95.9% des patients. La stimulation mono chambre représentait 87.8%. La stimulation double chambre représentait 12.8%. Les sondes ventriculaires étaient unipolaires chez 64% des patients. Toutes les sondes auriculaires étaient bipolaires. Les sondes à barbillon représentaient 82%. Les sondes à vis représentaient 18%. Les complications étaient: infection de la loge (4.1%), extériorisation de la sonde (2.9%), hématome de la poche (2.3%), déplacement de sonde (1.7%), pneumothorax/hémothorax (1.7%), thrombose veineuse axillaire (0.6%).

## Conclusion

Au Sénégal la pratique de la stimulation cardiaque est devenue une méthode thérapeutique très utilisée. Elle se fait aujourd’hui avec peu de complications graves. Sa pratique peut être cependant améliorée.

# Abstract: Evaluation de la fonction systolique du ventricule droit dans la Cardiomyopathie du Peripartum (CMPP) a l’hopital Aristide le Dantec a Dakar

**Authors:** RG Houndolo, MB Ndiaye, SA Sarr, MM Alaoui, KS Babaka, M Bodian, Ad Kane, M Diao, M Sarr, SA Ba

**Affiliations:** Clinique Cardiologique, Hôpital Aristide Le Dantec, Dakar, Sénégal; Clinique Cardiologique, Hôpital Aristide Le Dantec, Dakar, Sénégal; Clinique Cardiologique, Hôpital Aristide Le Dantec, Dakar, Sénégal; Clinique Cardiologique, Hôpital Aristide Le Dantec, Dakar, Sénégal; Clinique Cardiologique, Hôpital Aristide Le Dantec, Dakar, Sénégal; Clinique Cardiologique, Hôpital Aristide Le Dantec, Dakar, Sénégal; Clinique Cardiologique, Hôpital Aristide Le Dantec, Dakar, Sénégal; Clinique Cardiologique, Hôpital Aristide Le Dantec, Dakar, Sénégal; Clinique Cardiologique, Hôpital Aristide Le Dantec, Dakar, Sénégal; Clinique Cardiologique, Hôpital Aristide Le Dantec, Dakar, Sénégal

## Introduction

La cardiomyopathie du péripartum est caractérisée par une dysfonction systolique du ventricule gauche survenant entre le dernier mois de la grossesse et le cinquième mois du post-partum chez des patientes sans cardiopathies préexistantes. L’analyse de la fonction systolique du ventricule droit au cours de la CMPP est peu étudiée. L’objectif de notre étude était d’évaluer la prévalence de la dysfonction systolique du ventricule dans la CMPP.

## Résultats

19 cas de CMPP étaient étudiés. L’âge moyen était de 31.7 ans avec des extrêmes de 18 et 44 ans. La confrontation de tous les indices a montré 15 cas (78.9%) de dysfonction systolique du ventricule droit. L’altération de la FAC représentait 15 cas (100%). L’altération de la TAPSE était observée dans 12 cas (80%). L’altération de l’onde Sa tricuspide était diagnostiquée dans 10 cas (66.66%). L’altération du pic de vitesse de contraction isovolumétrique était constatée dans 13 cas (86.67%). L’altération de l’accélération myocardique isovolumétrique était identifiée dans 7 cas (46.67%). L’indice de TEI était anormal dans 13 cas (86.67%).

## Conclusion

Le raccourcissement de la surface du ventricule droit était l’indice le plus sensible. L’onde Sa tricuspide et l’accélération myocardique isovolumétrique étaient des indices spécifiques.

# Abstract: Prise en charge du syndrome coronarien aigu avec sus decalage du segment st (sca st+) au service de reanimation de l’Hopital Principal de Dakar

**Authors:** Isabelle Macoucou Kouame, Babacar Niang, Malick Niang, Bakary Diatta, Khalifa Ababacar Wade

**Affiliations:** Service de Cardiologie du CHU Aristide le Dantec, Dakar, Sénégal; Département d’Anesthésie, Réanimation, Hôpital Principal de Dakar, Sénégal; Département d’Anesthésie, Réanimation, Hôpital Principal de Dakar, Sénégal; Département d’Anesthésie, Réanimation, Hôpital Principal de Dakar, Sénégal; Département d’Anesthésie, Réanimation, Hôpital Principal de Dakar, Sénégal

## Introduction

L’infarctus du myocarde est une nécrose ischémique systématisée intéressant au moins 2 cm2 de surface ventriculaire. Il s’agit d’une urgence cardiologique absolue, pouvant engager le pronostic vital à court terme. Du fait de l’absence d’unité de soins intensifs cardiologiques à l’Hôpital Principal de Dakar, la prise en charge initiale se fait en réanimation. Les objectifs de cette étude étaient d’évaluer le profil épidémiologique, clinique, paraclinique, thérapeutique et évolutif à la phase aiguë.

## Méthodes

Il s’agit d’une étude rétrospective, allant de Jjanvier 2011 à décembre 2014. Etaient inclus tous les patients admis pour SCA ST+. Le diagnostic était posé sur des arguments cliniques ou électrocardiographiques, associés à une élévation des biomarqueurs de souffrance myocardique.

## Résultats

Nous avons inclus 41 patients. Le sex-ratio était de 2.72 en faveur des hommes. La moyenne d’âge était de 59.41 ans. Une hypertension artérielle était retrouvée chez 56.1% des patients. Un diabète était retrouvé chez 29.3% des patients et 33% d’entre eux avaient plus de 10 ans d’évolution. Une dyslipidémie était retrouvée chez 14.6% des patients, un tabagisme chez 31.7% des patients avec un nombre de paquets années moyen à 22.5. Des antécédents de SCA ST+ étaient retrouvés chez 4 patients, dont un ayant bénéficié d’un stent de nature non précisé. Les symptômes étaient dominés par la douleur thoracique dans 78% des cas, suivis des signes digestifs (7.3%). Les sujets diabétiques avaient présenté une douleur thoracique dans 58.3% des cas. Le délai moyen entre la survenue des symptômes et le premier contact médical était de 8.26 heures et 61% des patients étaient reçu avant la 12ème heure. A l’admission 75.6% des patients présentaient une lésion sous-épicardique. L’infarctus en territoire antérieur étendu était le plus retrouvé. L’examen retrouvait un stade I de Killip chez 87% des patients, et le délai moyen de prise en charge en réanimation après la pose du diagnostic était de 40.6 minutes.

La thrombolyse a été effectuée chez 20 patients (48.78%), la streptokinase a été utilisée chez 18 patients, l’atéplase chez 2 patients dans le cadre d’une thrombolyse pré-hospitalière. L’évolution initiale était favorable chez 31 patients (75.6%). Les principales complications étaient à type de choc cardiogénique (5 patients), de troubles du rythme ventriculaire (3 patients) et de bloc auriculo-ventriculaire complet nécessitant la mise en place d’une sonde d’entrainement électro systolique chez un patient. La durée moyenne d’hospitalisation était de 63.22 heures avec un taux de survie à 75.7%.

## Conclusion

La prise en charge du SCA ST+ pose de réels problèmes. Malgré l’évolution plutôt favorable observée dans notre étude cette dernière pourrait être améliorée par la création d’unités de soins intensifs cardiologiques et l’expansion des procédures de revascularisation.

# Abstract: Apport de l’echodoppler vasculaire dans le diagnostic lesionnel du pied diabetique au cours du diabete de type 2

**Authors:** M Pio, S Baragou, Y Afassinou, Akue E Goeh, KE Mossi, S Péssinaba, A Vignon, B Atta, K Ehlan, B Koudema

**Affiliations:** Service de Cardiologie, Centre Hospitalier Universitaire Sylvanus Olympio (CHU SO) de Lomé, Togo; Service de Cardiologie. Centre Hospitalier Universitaire Campus de Lomé, Togo; Service de Cardiologie, Centre Hospitalier Universitaire Sylvanus Olympio (CHU SO) de Lomé, Togo; Service de Cardiologie, Centre Hospitalier Universitaire Sylvanus Olympio (CHU SO) de Lomé, Togo; Service de Médecine Interne. Centre Hospitalier Universitaire Sylvanus Olympio de Lomé, Togo; Service de Cardiologie. Centre Hospitalier Universitaire Campus de Lomé, Togo; Service de Cardiologie, Centre Hospitalier Universitaire Sylvanus Olympio (CHU SO) de Lomé, Togo; Service de Cardiologie, Centre Hospitalier Universitaire Sylvanus Olympio (CHU SO) de Lomé, Togo; Service de Cardiologie, Centre Hospitalier Universitaire Sylvanus Olympio (CHU SO) de Lomé, Togo; Service de Cardiologie, Centre Hospitalier Universitaire Sylvanus Olympio (CHU SO) de Lomé, Togo

## Objectifs

Analyser et décrire l’aspect échodoppler des lésions ouvertes du pied chez le diabétique de type 2 et en évaluer les diagnostics lésionnels.

## Méthodes

Il s’agissait d’une étude prospective allant de septembre 2011 à janvier 2014. Elle s’est déroulée au centre d’explorations cardiovasculaires Saint Esprit de Lomé. L’étude a inclus les patients diabétiques de type 2 ayant présenté une lésion ouverte (plaie, ulcération, gangrène) du pied. Nous avons réalisé un échodoppler artériel et veineux chez tous les patients inclus dans l’étude.

## Résultats

L’étude a concerné 113 patients diabétiques de type 2. Le sex-ratio H/F était de 0.88. L’âge moyen était de 60.77 ans. Les indications des échodopplers vasculaires étaient: les gangrènes (38.08%), les plaies (31.97%), les ulcères (31.97%). L’échodoppler était normal chez 14 patients (12.4%) et a noté chez 99 patients (87.61%) des anomalies réparties en lésions artérielles 71.2% (74 patients) et en lésions veineuses 22.1% (25 patients). Les artériopathies étaient compensées chez 19% des patients et sévères chez 42% des patients. Les sténoses représentaient 31.7% des lésions totales et 44.6% des lésions artérielles. Les occlusions artérielles représentaient 18.3% des lésions totales et 25.7% des lésions artérielles. Les lésions veineuses étaient des insuffisances veineuses chroniques. Leurs étiologies étaient variqueuses (44%), post-phlébitiques (25%) et mixte (36%).

## Conclusion

Les artériopathies, les insuffisances veineuses chroniques des membres inférieurs sont des mécanismes vasculaires responsables des lésions ouvertes du pied diabétique type 2.

# Abstract: Athletes africains de haut niveau. Particularites electrocardiographique et echocardiographiques: analyse selon les criteres de la caf

**Authors:** EM Sarr, LL Diene, M Dioum, D Bindia, SJ Manga, A Diallo, M Leye, O Dieye, K Regnault, IB Diop

**Affiliations:** Clinique cardiologique du CHU Fann, Dakar, Senegal; Clinique cardiologique du CHU Fann, Dakar, Senegal; Clinique cardiologique du CHU Fann, Dakar, Senegal; Clinique cardiologique du CHU Fann, Dakar, Senegal; Clinique cardiologique du CHU Fann, Dakar, Senegal; Clinique cardiologique du CHU Fann, Dakar, Senegal; Clinique cardiologique du CHU Fann, Dakar, Senegal; Clinique cardiologique du CHU Fann, Dakar, Senegal; Clinique cardiologique du CHU Fann, Dakar, Senegal; Clinique cardiologique du CHU Fann, Dakar, Senegal

## Introduction

L’activité sportive de haut niveau peut induire des modifications électriques et échcardiographiques connues sous la dénomination générique de ‘ coeur d’athlète’. Le screening systématique permet la détection de ces modifications physiologiques qui doivent être différentiées des pathologies.

## Methodes

Il s’agit d’une étude descriptive allant de Septembre 2014 à Mai 2015, d’un groupe de sportifs africains de haut niveau, de nationalités différentes et de disciplines sportives variées. Tous ces athlètes avaient bénéficié d’un examen clinique complété par la réalisation d’un électrocardiogramme et d’une échocardiographie Doppler. L’échocardiographie était réalisée selon les critères de la Confédération Africaine de Football (CAF) avec indexation des paramètres à la surface corporelle.

## Resultats

Notre étude portait sur une population de 68 sportifs. L’âge variait de 16 à 29 ans (moyenne de 19.3 ± 10.1 ans). L’index de masse corporelle moyen était de 21.8. La durée moyenne d’entrainement par semaine était de 12.3 heures. Le football était la discipline la plus pratiquée suivi du sprint et de la course de fond. Les anomalies électrocardiographiques étaient les suivantes: dix athlètes (14.7%) avaient des troubles de la repolarisation, six athlètes (8.9%) avaient une hypertrophie ventriculaire gauche. A l’échocardiographie Doppler on retrouvait une augmentation de la masse ventriculaire indexée chez six athlètes (8.9%), une dilatation modérée de l’oreillette gauche (1.4%), une dilatation modérée de l’oreillette droite (1.4%) et une dilatation moyenne des deux oreillettes (1.4%).Une cardiomyopathie dilatée hypokinétique avec altération moyenne de la fonction systolique globale du ventricule gauche était retrouvée chez un athlète.

## Conclusion

Chez le sportif très entraîné l’électrocardiogramme couplé à l’échocardiographie apporte des données essentielles qui permettent de distinguer les modifications physiologiques des cas pathologiques conduisant à des mesures préventives adaptées.

# Abstract: Apport et specificites du speckle tracking (strain) dans le diagnostic et la prise en charge des cardiopathies ischemiques et des valvulopathies evoluees: experience du Sénégal

**Authors:** EM Sarr, O Dieye, K Regnault, M Dioum, D Bindia, SJ Manga, LL Diene, A Diallo, M Leye, IB Diop

**Affiliations:** Clinique cardiologique du CHU Fann, Dakar, Senegal; Clinique cardiologique du CHU Fann, Dakar, Senegal; Clinique cardiologique du CHU Fann, Dakar, Senegal; Clinique cardiologique du CHU Fann, Dakar, Senegal; Clinique cardiologique du CHU Fann, Dakar, Senegal; Clinique cardiologique du CHU Fann, Dakar, Senegal; Clinique cardiologique du CHU Fann, Dakar, Senegal; Clinique cardiologique du CHU Fann, Dakar, Senegal; Clinique cardiologique du CHU Fann, Dakar, Senegal; Clinique cardiologique du CHU Fann, Dakar, Senegal

## Introduction

Le speckle tracking encore appelé strain est une imagerie de déformation cardiaque qui permet une étude précise et fiable de l’activité myocardique. Le strain longitudinal permet une étude topographique précise et une quantification de l’ischémie. Il permet aussi une évaluation précise de la réserve contractile dans les dysfonctions systoliques sévères du ventricule gauche secondaires aux valvulopathies évoluées.

## Méthodes

Il s’agit d’une étude prospective allant de Novembre 2014 à Juillet 2015, de patients ayant bénéficié d’une échocardiographie de repos couplé à un strain longitudinal. Dans le cadre de la recherche de viabilité myocardique et de la réserve contractile, le strain était réalisé au pic de l’échocardiographie de stress à la dobutamine. Les indications étaient dominées par la recherche d’ischémie myocardique, de viabilité myocardique et de réserve contractile. On observait trois groupes de patients: les cardiopathies ischémiques, les cardiopathies hypertrophiques et les valvulopathies. Tous les patients avaient bénéficié d’une échocardiographie bidimensionnelle et d’un strain longitudinal.

## Résultats

Notre étude portait sur une population de dix-sept patients. L’âge moyen de nos patients était de 58.7 ± 11.6 ans. Le strain longitudinal était normal chez cinq patients (29.4%) et pathologique dans 70.6% des cas. La viabilité myocardique était retrouvée chez trois patients (17.6%).Trois patients (17.6%) avaient présenté une bonne réserve contractile du ventricule gauche avec une variation de 4% du strain global entre l’effort et le repos. La recherche de réserve contractile négative pour 30% des patients.

## Conclusion

L’imagerie de strain appliquée à l’échocardiographie de stress permet une approche topographique et quantitative plus précise des lésions myocardiques. Ces explorations plus accessibles et moins onéreuses que les explorations classiques, comme les isotopes, ont un impact considérable sur la fiabilité des indications de revascularisation et des indications opératoires.

# Abstract: Apport de l’echocardiographie de stress dans la prise en charge des cardiopathies ischemiques et des valvulopathies evoluees: experience de la clinique cardiologique du chu fann

**Authors:** EM Sarr, O Dieye, K Regnault, M Dioum, D Bindia, SJ Manga, LL Diene, A Diallo, M Leye, IB Diop

**Affiliations:** Clinique cardiologique du CHU Fann, Dakar, Senegal; Clinique cardiologique du CHU Fann, Dakar, Senegal; Clinique cardiologique du CHU Fann, Dakar, Senegal; Clinique cardiologique du CHU Fann, Dakar, Senegal; Clinique cardiologique du CHU Fann, Dakar, Senegal; Clinique cardiologique du CHU Fann, Dakar, Senegal; Clinique cardiologique du CHU Fann, Dakar, Senegal; Clinique cardiologique du CHU Fann, Dakar, Senegal; Clinique cardiologique du CHU Fann, Dakar, Senegal; Clinique cardiologique du CHU Fann, Dakar, Senegal

## Introduction

L’échocardiographie de stress est une technique d’investigation de la maladie coronaire et des valvulopathies. Elle représente actuellement une véritable entité au sein et une véritable alternative aux techniques isotopiques. Son principe est de provoquer par un stress myocardique une ischémie responsable d’une anomalie de la contraction régionale du ventricule gauche détectable par échographie cardiaque bidimensionnelle.

## Methodes

Il s’agit d’une étude prospective allant de Novembre 2014 à Juillet 2015, de patients ayant bénéficié d’une échocardiographie de stress à la dobutamine.

Les indications étaient dominées par la recherche d’ischémie myocardique, de viabilité myocardique et de réserve contractile. On observait trois groupes de patients : les cardiopathies ischémiques, les cardiopathies hypertrophiques et les valvulopathies. Tous les patients avaient bénéficié d’une échocardiographie bidimensionnelle et d’une échocardiographie de stress.

## Resultats

Notre étude portait sur une population de dix-sept patients. L’âge moyen de nos patients était de 58.7 ± 11.6 ans. Une ischémie myocardique était déclenchée chez deux patients (11.7%). La viabilité myocardique était retrouvée chez trois patients (17.6%).Trois patients (17.6%) avaient présenté une bonne réserve contractile du ventricule gauche. L’échocardiographie de stress était négative pour 30% des patients.

## Conclusion

L’échocardiographie de stress est devenue actuellement une exploration fonctionnelle à part entière. L’échocardiographie sous dobutamine est la modalité préférentielle pour la détection d’une viabilité myocardique avant revascularisation. C’est une exploration plus accessible et moins onéreuse que les isotopes et qui a un impact considérable sur la fiabilité des indications de revascularisation et des indications opératoires.

# Abstract: Dissection aortique sur volumineux anevrysme de l’aorte ascendante compliquant un syndrome de marfan

**Authors:** Simon Antoine Sarr, Malick Bodian, Geraud Akpo, Mouhamadou Bamba Ndiaye, Kana Babaka, Malick Ndiaye, Mame Madjiguéne Ka, Tening Diouf, Youssou Diouf, Faad Chaara, Meryem Lali, Adama Kane, Maboury Diao, Moustapha Sarr, Serigne Abdou Ba

**Affiliations:** Cardiologie CHU Aristide Le Dantec, Dakar, Senegal; Cardiologie CHU Aristide Le Dantec, Dakar, Senegal; Radiologie CHU Aristide Le Dantec de Dakar; Cardiologie CHU Aristide Le Dantec, Dakar, Senegal; Cardiologie CHU Aristide Le Dantec, Dakar, Senegal; Cardiologie CHU Aristide Le Dantec, Dakar, Senegal; Cardiologie CHU Aristide Le Dantec, Dakar, Senegal; Cardiologie CHU Aristide Le Dantec, Dakar, Senegal; Cardiologie CHU Aristide Le Dantec, Dakar, Senegal; Clinique Marrakech, Morocco; Clinique Marrakech, Morocco; Cardiologie CHU Aristide Le Dantec, Dakar, Senegal; Cardiologie CHU Aristide Le Dantec, Dakar, Senegal; Cardiologie CHU Aristide Le Dantec, Dakar, Senegal; Cardiologie CHU Aristide Le Dantec, Dakar, Senegal

## Introduction

Le syndrome de Marfan est une affection rare qui atteint le tissu de soutien. Les complications cardio-vasculaires notamment la dissection aortique en font toute la gravité. Elles justifient un monitorage des dimensions de la racine de l’aorte. Nous rapportons le cas de monsieur AD qui a présenté une dissection aortique sur maladie de Marfan.

## Observation

Monsieur AD est un patient de 29 ans, reçu en consultation pour des palpitations épisodiques isolées. L’interrogatoire ne retrouvait pas de douleur ni d’autre symptôme. Elle relevait, par contre, trois cas de mort subite dans la famille dont deux dans un contexte de douleur thoracique aiguë. L’examen notait un morphotype de évocateur d’un syndrome de Marfan, un souffle diastolique latéro-sternal gauche d’intensité 3/6 avec un souffle systolique d’accompagnement. L’électrocardiogramme inscrivait une hypertrophie ventriculaire gauche diastolique. La radiographie thoracique montrait un élargissement important du médiastin. L’échocardiographie Doppler mettait en évidence une dissection de l’aorte s’étendant jusqu’en aval de l’origine des troncs supra-aortiques. Il y avait un anévrysme volumineux de l’aorte initiale qui atteignait 82 mm de diamètre maximal, une fuite aortique sévère, un ventricule gauche dilaté. L’angioscanner thoracique confirmait la dissection aortique de type A de Stanford avec un anévrysme fusiforme de la portion ascendante dont le diamètre transverse maximal était mesuré à 80 mm. Il y avait, par ailleurs, des lésions emphysémateuses sous pleurales apicales bilatérales. Une intervention de Bentall avait été faite avec mise en place d’un tube valvé et réimplantation des ostia coronaires. L’évolution était favorable.

## Conclusion

La dissection aortique est une complication redoutable du syndrome de Marfan. La chirurgie, faite en curatif ou mieux en préventif, permet d’améliorer le pronostic de ces patients.

# Abstract: Coeur triatrial gauche: a propos de deux cas

**Authors:** Simon Antoine Sarr, Malick, Ravaovy Hariniaina Bodian, Hariniaina Ravaovy, Mouhamadou Bamba Ndiaye, Malick Ndiaye, Mame Madjiguéne Ka, Tening Diouf, Youssou Diouf, Adama Kane, Maboury Diao, Moustapha Sarr, Serigne Abdou Ba

**Affiliations:** Cardiologie CHU Aristide Le Dantec, Dakar, Senegal; Cardiologie CHU Aristide Le Dantec, Dakar, Senegal; Cardiologie CHU Aristide Le Dantec, Dakar, Senegal; Cardiologie CHU Aristide Le Dantec, Dakar, Senegal; Cardiologie CHU Aristide Le Dantec, Dakar, Senegal; Cardiologie CHU Aristide Le Dantec, Dakar, Senegal; Cardiologie CHU Aristide Le Dantec, Dakar, Senegal; Cardiologie CHU Aristide Le Dantec, Dakar, Senegal; Cardiologie CHU Aristide Le Dantec, Dakar, Senegal; Cardiologie CHU Aristide Le Dantec, Dakar, Senegal; Cardiologie CHU Aristide Le Dantec, Dakar, Senegal; Cardiologie CHU Aristide Le Dantec, Dakar, Senegal

## Introduction

Le coeur triatrial est une malformation congénitale rare dans laquelle l’oreillette est subdivisée en deux chambres distinctes par une membrane fibro-musculaire. Classiquement à gauche, la symptomatologie rejoint celle de la sténose mitrale s’il est dans la forme symptomatique, c’est-à-dire les types 1 et 2 selon la classification de Loeffler. Mais il reste asymptomatique dans la majeure partie des cas.

## Observation

Nous rapportons le cas de deux patients, une femme et un homme âgés tous deux de 38 ans. Ils sont venus consulter pour une dyspnée d’effort d’aggravation progressive associée à des palpitations chez la première. L’examen clinique retrouvait chez cette dernière des bruits du coeur irréguliers, un roulement diastolique au foyer mitral, un éclat du B2 pulmonaire. Chez le second, il notait un roulement diastolique discret et un éclat du second bruit. L’électrocardiogramme inscrivait une arythmie complète par fibrillation auriculaire et une hypertrophie ventriculaire droite chez l’un, une hypertrophie auriculaire gauche chez le second. L’échocardiogramme concluait chez la première à un coeur triatrial associé à une sténose mitrale rhumatismale serrée. Chez le second, il concluait à un coeur triatrial gauche avec une membrane calcifiée sténosante confirmé à la tomodensitométrie. Il y avait dans les deux cas une hypertension artérielle pulmonaire importante.

## Conclusion

Le coeur triatrial est une affection rare pouvant coexister avec une valvulopathie rhumatismale.

# Abstract: Complications cardio-vasculaires du syndrome de marfan: resultats d’une enquete familiale

**Authors:** Simon Antoine Sarr, Malick Bodian, Mouhamadou Bamba Ndiaye, Malick Ndiaye, Mame Madjiguéne Ka, Tening Diouf, Youssou Diouf, Adama Kane, Maboury Diao, Moustapha Sarr, Serigne Abdou Ba

**Affiliations:** Cardiologie CHU Aristide Le Dantec, Dakar, Senegal; Cardiologie CHU Aristide Le Dantec, Dakar, Senegal; Cardiologie CHU Aristide Le Dantec, Dakar, Senegal; Cardiologie CHU Aristide Le Dantec, Dakar, Senegal; Cardiologie CHU Aristide Le Dantec, Dakar, Senegal; Cardiologie CHU Aristide Le Dantec, Dakar, Senegal; Cardiologie CHU Aristide Le Dantec, Dakar, Senegal; Cardiologie CHU Aristide Le Dantec, Dakar, Senegal; Cardiologie CHU Aristide Le Dantec, Dakar, Senegal; Cardiologie CHU Aristide Le Dantec, Dakar, Senegal; Cardiologie CHU Aristide Le Dantec, Dakar, Senegal

## Introduction

Le syndrome de Marfan est une affection rare qui atteint le tissu de soutien. C’est une maladie à transmission autosomique dominante qui résulte en règle générale d’une mutation du gène de la fibrilline de type 1. Son pronostic fonctionnel dépend des atteintes ophtalmiques et rhumatologiques. Par contre le pronostic vital est lié aux atteintes cardio-vasculaires notamment aortiques. Nous rapportons les résultats d’une enquête familiale ayant fait suite à la découverte d’un cas de dissection aortique sur syndrome de Marfan.

## Méthodes

Tous les membres de la famille était convoqués. L’évaluation était clinique, électrocardiographique et échocardiographique. Un angioscanner était fait en cas d’anomalies aortiques retrouvées à l’échocardiographie.

## Résultats

Monsieur AD est un patient de 29 ans porteur d’un syndrome de Marfan compliqué d’une dissection aortique avec un anévrysme très important de l’aorte initiale (82 mm) et une fuite aortique sévère. Il appartient à une des trois fratries issues de la même mère, mais de trois pères différents. L’interrogatoire retrouvait 3 cas de mort subite dont deux dans un contexte de douleur thoracique brutale. Quatre sujets avaient été évalués. Ils présentaient tous un syndrome de Marfan. Des douleurs thoraciques anciennes étaient rapportées dans un cas. L’examen clinique retrouvait un souffle systolique dans trois cas, un souffle diastolique dans un cas. L’électrocardiogramme inscrivait une hypertrophie ventriculaire gauche dans deux cas. L’échocardiographie Doppler retrouvait une ballonisation de la grande valve mitrale avec une fuite modérée dans trois cas, une dissection de l’aorte initiale qui était anévrysmale associée à une fuite aortique modérée dans un cas. Elle retrouvait aussi un cas de dilatation modérée et isolée de l’aorte initiale. L’angioscanner confirmait les anomalies aortiques dans les deux cas notamment la dissection type A de Stanford dont le diamètre transverse était mesuré à 60 mm.

## Conclusion

Cette enquête familiale passe en revue les complications cardio-vasculaires au cours du syndrome de Marfan. Elles peuvent être asymptomatiques d’où l’intérêt du dépistage familiale.

# Abstract: Hta et grossesse au niger: aspects epidemiologiques et complications foetomater-nelles

**Authors:** I Toure, A Mahamane, M Aichatou, O Bonkano, F Zakari

**Affiliations:** Service de médécine cardiologie, CHU Lamorde Niamey, Niger; Service de médécine cardiologie, CHU Lamorde Niamey, Niger; Service de médécine cardiologie, CHU Lamorde Niamey, Niger; Service de médécine cardiologie, CHU Lamorde Niamey, Niger; Service de médécine cardiologie, CHU Lamorde Niamey, Niger

## Introduction

Les désordres hypertensifs de la grossesse (HTAG) représentent une pathologie fréquente et dangereuse car constituent la 3éme cause de mortalité maternelle et la première cause de mortalité périnatale; ils représentent en Afrique le 1er groupe nosologique de la pathologie cardiovasculaire gravidique et sont responsables des complications sévères qui touchent non seulement la mère mais aussi le foetus. Le but de notre travail est d’étudier les aspects épidémiologiques et cliniques de HTAG, de ses différents types en fonction du nombre et de la qualité des consultations prénatales (CPN) afin de formuler les recommandations pour son diagnostic, sa prise en charge et surtout la prévention de ses complications.

## Méthodes

1° méthodologie: Nous avons entrepris une 1ère étude prospective en salle d’accouchement d’ avril 2011 à novembre 2013 à propos de 540 cas de parturientes toutes hypertendues (sur 6 120 accouchées récentes soit 8.9%), âgées de 13 à 44 ans avec un âge moyen de 24.57 ans dont 70% ont moins de 30 ans parmi lesquelles on retrouve 85.18% de femmes mariées,14.82% de celibataires, 84.63% de femmes au foyer.

## Résultats

On note que 65% (351) n’ont pas fait de consultation prénatale (CPN0), 80 (14.81%) CPN1, 61 (11.28%) CPN2, 27 (5%) CPN3, et seulement 21 (3.88%) CPN4. Parmi les 332 primipares 260 ont CPN0 et seulement 12 ont au moins CPN 4. Au terme de première partie on note: 256 cas (47.40%) d’éclampsie, 36 cas (6.6%) d’HRP. On note une prévalence saisonnière des poussées hypertensives à 39.9% pendant la saison des pluies (juin, juillet, aout).

## Méthodes

2° méthodologie: Nous avons mené une 2ème étude prospective, descriptive et analytique sur une période de11 mois allant du 1er décembre 2013 au 30 Octobre 2014 qui a concerné 1 480 parturientes, ayant toutes eu au moins CPN4 et nous les avons suivies jusqu’à l’accouchement. Nous avons eu 175 cas d’HTA associée à la grossesse sur les 1 480 parturientes soit un taux de 11.82%. Parmi les 1 305 normo-tendues restantes, nous avons apparié 175 témoins sur les critères d’âge, âge gestationnel et gestité. La collecte s’est faite sur des fiches préétablies.

## Résultats

Au terme de notre étude nous avons trouvé les résul résultats suivants: l’HTAG, la pré-éclampsie ont été les types d’HTA les plus rencontrés avec respectivement 53.14 et 32.57%.

Les complications, qu’elles soient maternelles ou foetales, sont l’apanage des formes sévères de l’hypertension: elles sont moins fréquentes dans ce 2ème lot que chez celles du 1er lot n’ayant pas eu de CPN systématiques et régulières.

## Conclusion

notre étude révèle que les parturientes hypertendues n’ayant pas eu de CPN ont plus de complications que celles qui en ont eu. L’importance de la qualité, du nombre et de la régularité des CPN bien conduites, est capitale pour améliorer la prise en charge des parturientes hypertendues et réduire la mortalité et la morbidité foeto-maternelles ainsi que pour prévenir les complications.

# Abstract: Les cardiopathies congenitales au niger aspects epidemiologiques et cliniques a propos de 478 cas

**Authors:** I Toure, A Mahamane, M Aichatou, O Bonkano, F Zakari

**Affiliations:** Service de médécine cardiologie, CHU Lamorde Niamey, Niger; Service de médécine interne, Facilte de science et de la Sante, Niger; Service de médécine interne, Facilte de science et de la Sante, Niger; Service de médécine interne, Facilte de science et de la Sante, Niger; Service de médécine interne, Facilte de science et de la Sante, Niger

## Introduction

Les cardiopathies congénitales constituent une des pathologies cardiaques les plus fréquentes de l’enfant.

## Méthodes

Nous avons entrepris une étude clinique et échographique de janvier 2002 à juin 2014, soit treize ans et demi, ce qui nous a permis de colliger 521 cas soit 22.77% de l’ensemble des échographies réalisées dans notre service pendant la même période.

## Résultats

Parmi ces cas, 521 cas 478 soit 20.89% des cas étaient exploitables.

Ainsi, les principales cardiopathies congénitales étaient la CIV 51.88% suivi de la CIA 19.03%, les CAV 9.2%, des tétralogies de Fallot 8.36% ; sur l’ensemble de ces cardiopathies, on note une prédominance masculine de 51.8% contre 48.42 % de cas de sexe féminin. L’âge moyen était de 18 mois avec des extrêmes de 3 jours à 17 ans, mais le 2/3 de nos patients ont moins de 1 an.

## Conclusion

Les auteurs insistent sur le dépistage précoce et la prise en charge adéquate de ces cardiopathies dont certaines de bons pronostics une fois opérées peuvent guérir définitivement. Mais malheureusement le Niger à l’instar de nombreux pays africains ne possède pas de centre de chirurgie cardiaque ce qui pose beaucoup de problèmes quant à la prise en charge de ces jeunes patients.

# Abstract: Prevalence des maladies cardiovasculaires chez la femme en milieu tropical africain au Niger

**Authors:** I Toure, A Mahamane, M Aichatou, O Bonkano, F Zakari

**Affiliations:** Service de médécine cardiologie, CHU Lamorde Niamey, Niger; Service de médécine cardiologie, CHU Lamorde Niamey, Niger; Service de médécine cardiologie, CHU Lamorde Niamey, Niger; Service de médécine cardiologie, CHU Lamorde Niamey, Niger; Service de médécine cardiologie, CHU Lamorde Niamey, Niger

## Méthodes

Durant la période du 1er janvier 2012 au 31 décembre 2014: 4 264 patients toutes pathologies confondues ont été hospitalisés dans notre service. A l’aide des critères établis, nous avions identifié 1 677 patients sans distinction de sexe ni d’âge admis pour MCV.

## Resultats

Au terme de notre étude, 1 677 patients étaient hospitalisés dont 815 femmes et 862 hommes. La prévalence des MCV étaient de 34.4%. Le sexe ratio était de 1.05 en faveur des hommes. L’âge moyen était de 57.7 ans. Chez les femmes, la tranche d’âge de plus de 60 ans était la plus touchée avec 56.06%. L’aphasie et l’hémiplégie était de loin le premier motif de consultation chez les femmes avec 21,6% suivi de l’HTA 15%. L’AVC, l’IC et l’HTA étaient les pathologies les plus rencontrées chez les femmes avec respectivement 28.9, 24.5 et 21.6%. Les autres pathologies fréquentes étaient la cardiomyopathie du post partum (7.7%). L’AVC était la pathologie la plus meurtrière chez la femme (22.6%) suivie de l’insuffisance cardiaque 17.8%, l’HTA 15.7% et le syndrome coronarien aigu 9.5%.

## Conclusion

La pathologie cardio vasculaire de la femme africaine est une réalité au même titre que celle de l’homme, malgré le contexte de sous-développement un accent particulier doit être mis sur la prise en charge cardiovasculaire des femmes en Afrique.
